# Genome-wide identification and classification of MIKC-type MADS-box genes in Streptophyte lineages and expression analyses to reveal their role in seed germination of orchid

**DOI:** 10.1186/s12870-019-1836-5

**Published:** 2019-05-28

**Authors:** Chunmei He, Can Si, Jaime A. Teixeira da Silva, Mingzhi Li, Jun Duan

**Affiliations:** 1 Key Laboratory of South China Agricultural Plant Molecular Analysis and Gene Improvement, South China Botanical Garden, Chinese Academy of Sciences, Guangzhou, 510650 China; 2 University of the Chinese Academy of Sciences, Beijing, 100049 China; 3 Independent, Miki-Cho, Japan; 4 Genepioneer Biotechnologies Co. Ltd, Nanjing, 210014 China

**Keywords:** Flower formation, Phylogenetic analysis, Classification, Gene family expansion

## Abstract

**Background:**

MADS-box genes play crucial roles in plant floral organ formation and plant reproductive development. However, there is still no information on genome-wide identification and classification of MADS-box genes in some representative plant species. A comprehensive investigation of MIKC-type genes in the orchid *Dendrobium officinale* is still lacking.

**Results:**

Here we conducted a genome-wide analysis of MADS-box proteins from 29 species. In total, 1689 MADS-box proteins were identified. Two types of MADS-box genes, termed type I and II, were found in land plants, but not in liverwort. The SQUA, DEF/GLO, AG and SEP subfamilies existed in all the tested flowering plants, while SQUA was absent in the gymnosperm *Ginkgo biloba*, and no genes of the four subfamilies were found in a charophyte, liverwort, mosses, or lycophyte. This strongly corroborates the notion that clades of floral organ identity genes led to the evolution of flower development in flowering plants. Nine subfamilies of MIKC^C^ genes were present in two orchids, *D. officinale* and *Phalaenopsis equestris*, while the TM8, FLC, AGL15 and AGL12 subfamilies may be lost. In addition, the four clades of floral organ identity genes in both orchids displayed a conservative and divergent expression pattern. Only three MIKC-type genes were induced by cold stress in *D. officinale* while 15 MIKC-type genes showed different levels of expression during seed germination.

**Conclusions:**

MIKC-type genes were identified from streptophyte lineages, revealing new insights into their evolution and development relationships. Our results show a novel role of MIKC-type genes in seed germination and provide a useful clue for future research on seed germination in orchids.

**Electronic supplementary material:**

The online version of this article (10.1186/s12870-019-1836-5) contains supplementary material, which is available to authorized users.

## Background

The regulation of gene expression is a complex control mechanism that is coordinated by a number of mechanisms, including by transcription factors (TFs). TFs specifically recognize *cis*-regulatory regions of target genes, then regulate their expression in a way that leads to a wide range of physiological and biochemical processes. They also modulate developmental processes in plants [1]. MADS-box TFs, an ancient family of TFs found in both green algae charophyceans and land plants, play an essential role in the evolution of flower architecture [2, 3]. MADS-box genes encode proteins that share a highly conservative DNA-binding domain, the MADS domain, which recognizes similar 10-bp A/T-rich DNA sequences, the CArG-box [4].

Numerous MADS-box proteins have been identified from green algae, moss, gymnosperms and angiosperms [5]. The number of MADS-box genes varies greatly from species to species in plant lineages, suggesting that they are involved in the regulation of clade-specific functions. In the charophyceans [3], one MADS-box gene in *Marchantia polymorpha* [6], 26 in *Physcomitrella patens* [7] and 19 in *Selaginella moellendorffii* [8] have been reported. In angiosperms, dozens or even more than 100 MADS-box genes have been identified in dicots, including *Arabidopsis thaliana* [9], *Brassica rapa* (Chinese cabbage) [10], and *Vitis vinifera* [11]. In monocots, dozens of MADS-box genes have been found in *Zea mays* (75) [12], *Sorghum bicolor* (65) [12], *Brachypodium distachyon* (57) [13] and *Oryza sativa* (75) [14].

In plants, MADS-box genes can be divided into two distinct groups, namely type I and type II lineages: type I MADS-box proteins have no keratin-like (K) domain and only have the MADS (M) domain, whereas type II proteins also possess an intervening (I) domain, a K domain, and a C-terminal region followed by an M domain [15]. The type II MADS-box proteins are also known as MIKC-type MADS-box proteins in plants, comprising MIKC^C^ and MIKC* proteins [15, 16]. The MIKC^C^ proteins can be further subdivided into at least 13 distinct subfamilies (AG, AGL6, AGL12, AGL15, AGL17, BS, DEF/GLO, FLC, SEP, SQUA, SVP, TM3/SOC1 and TM8) on the basis of phylogenetic analysis [15].

The function of type I MADS-box genes is unclear. However, type II MADS genes are well studied and play a crucial role in the control of flower development. Investigations of type II MADS genes have led to the establishment of a well know model for the development of floral organs, the ABCDE model. A + E class genes establish the identity of sepals, A + B + E class genes establish the identity of petals, B + C + E class genes establish the identity of stamens, and D + E class genes establish the identity of carpels. ABCDE homeotic genes have been comprehensively studied in angiosperms, particularly in *Antirrhinum majus* and *A. thaliana* [17]. In Arabidopsis, *AP1* is an A class gene, *AP3* and *PI* are B class genes, *AG* is a C class gene, *STK* and *SHP1*/*2* are D class genes, and *SEP1*–*4* are E class genes [18]. The expression of ABCDE class genes is specific to different floral organs. For example, AP3 and PI homologs are expressed in petals and stamens, AG homologs are expressed in stamens and carpels, while SEP homologs are expressed in all floral organs in angiosperms, even in basal angiosperms [18–21]. MADS-box genes serve an important function by linking evolution and development, and should aid in understanding the mechanisms underlying flower formation while providing a foundation for analyzing variations in plant floral architecture. Therefore, it is important to investigate how MADS-box genes have evolved in plants.

To obtain a comprehensive view of MADS-box genes in plants, in this study we conducted a genome-wide identification of MADS-box genes from a broad range of plants across six lineages, including chlorophytes, a charophyte, a liverwort, mosses, a lycophyte, a gymnosperm and representatives of several angiosperm plant lineages whose genomes were available. The representative species, which belong to six lineages of plants (chlorophytes, charophyte, liverwort, mosses, lycophyte, gymnosperm) were selected to analyze as much of their genomes as was available. The species from the angiosperm lineage were selected because of their importance in human life. Genomic sequencing technology and bioinformatics tools have become faster and more efficient, and thus increasingly more genomic sequences of plants are currently available. Therefore, it is necessary to reanalyze MADS-box genes in important as well as a wide range of plant species, as has been done in this study, to identify this gene family in other plant species to reveal its evolution.

Orchids belong to the Orchidaceae and have unique floral patterning, particularly floral structures and organ identity. The labellum (the ‘lip’) and gynostemium (the ‘column’, a fused structure consisting of stamens and pistils) reflect unique development of orchid flowers. Several studies have isolated and analyzed of ABCE genes in orchids [22]. For example, the ABCE function genes in *Dendrobium* [23–25], B function genes (AP3 and PI lineages) in *Oncidium* [26], B and E function genes in *Cymbidium* [27], and B (AP3 lineages) in *Phalaenopsis* [28] were identified by RT-PCR and characterized. *Dendrobium officinale* and *Phalaenopsis equestris* are two important orchids with ornamental value. *Dendrobium* and *Phalaenopsis* diverged roughly 40 million years ago (Ma) [29]. The sizes of both *P. equestris* and *D*. *officinale* genomes are similar and exceed 1.1 gigabytes (Gb). The size of the *P. equestris* genome is 1.16 Gb, which was assessed by a whole-genome shotgun sequence and assembly strategy [30]. The size of the *D*. *officinale* genome is 1.11 Gb and was assessed by a K-mer analysis [31]. In this study, the ABCE genes of two orchids were identified using whole genomes, and phylogenetic and comparative expression analyses were performed. The expression patterns of MIKC genes in *D. officinale* under cold stress and during seed germination were also analyzed. These results may provide important information to understand the conservation and divergence of these genes’ functions, providing clues to explore the function of this gene family.

## Results

### Identification of MADS-box genes in multiple plant species

**Fig. 1:**
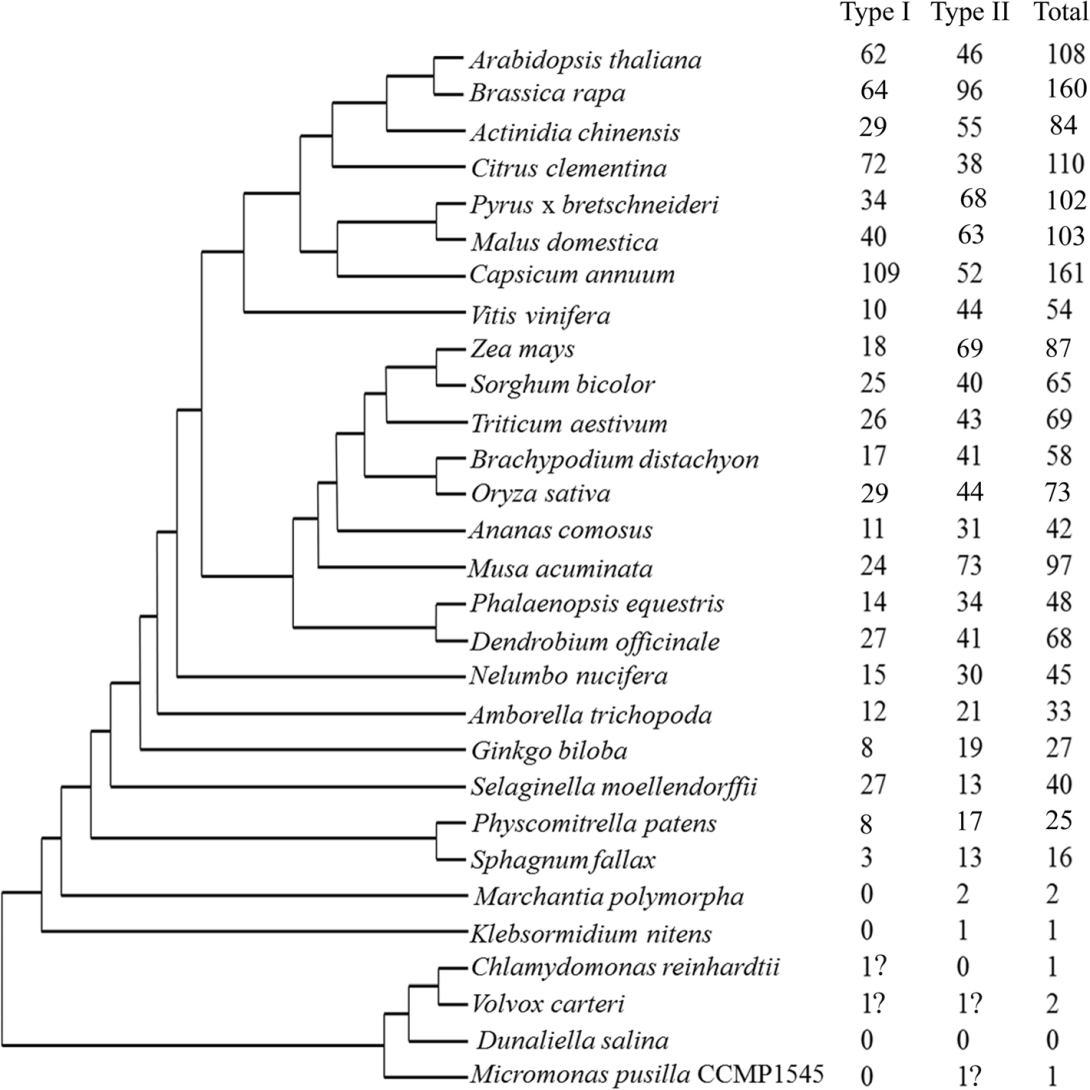
The corresponding number of type I, type II and total MADS-box genes for each plant species identified in this study. The tree topology reflects a consensus of plant phylogenies [32]. The ‘?’ indicates that the genes contained an M domain, but shared low identity with other MADS-box proteins in higher plants

In total, 1689 MADS-box genes, including 684 type I and 1001 type II MADS-box genes, as well as four MADS-box genes from chlorophytes, were identified from entire genome sequences of 29 plant species (Fig. 1; Additional file 1: Table S1). Lower plant (chlorophyte) lineages had no or few MADS-box genes in their genomes. For example, no MADS-box gene was found in *Dunaliella salina*, only one MADS-box gene was observed in *Micromonas pusilla* and in *Chlamydomonas reinhardtii*, and there were just two MADS-box genes in *Volvox carteri* (Fig. 1). All four MADS-box genes from chlorophyte lineages only contain the M domain, and have no I, K, or C domains (Additional file 2: Figure S1), which agrees with findings in other green algae belonging to the chlorophyte lineage [5]. The basal streptophyte, *K*. *nitens* has only one MADS-box gene, which is a type II MADS-box TF that contains the M, I, K, and C domains (Additional file 3: Figure S2). The type I genes were detected in the genomes of all mosses, the lycophyte, the gymnosperm and angiosperms, but not in the charophyte and liverwort (Fig. 1). However, type II genes were widely present among all streptophyte lineages (Fig. 1). In addition, the number of MADS-box genes in angiosperms were numerous, ranging from 33 (*A. trichopoda*) to 169 (*M. domestica*), but only one or two in algae (Fig. 1).

### Phylogenetic analysis, classification of type II (MIKC-type) genes, and analysis of the ABCDE genes in Streptophytes

**Fig. 2:**
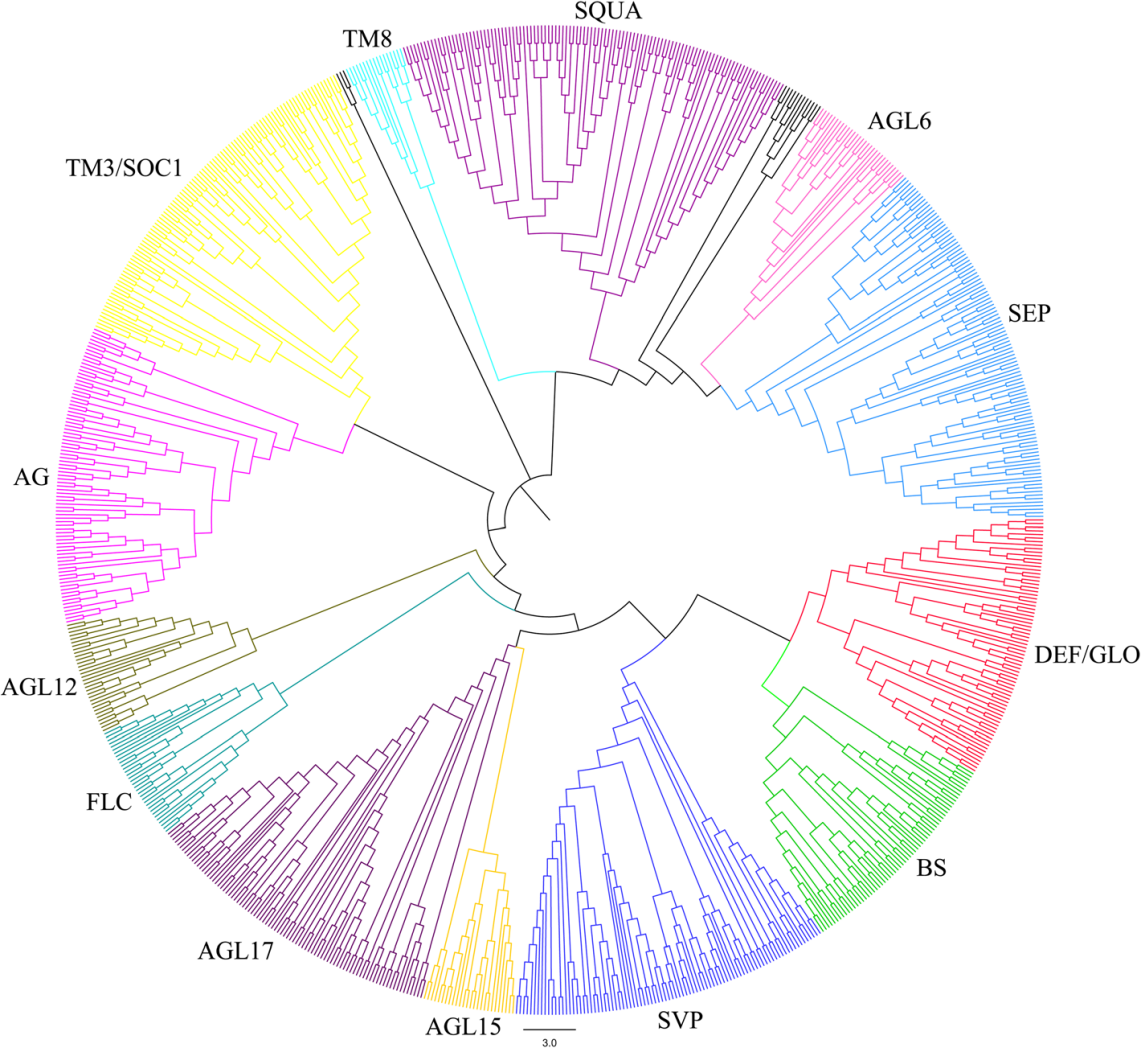
Classification of the total of 865 MIKC^C^ proteins based on phylogenetic analysis. The MIKC^C^ proteins were divided into 13 subfamilies: AG, AGL6, AGL12, AGL15, AGL17, BS, DEF/GLO, FLC, SEP, SQUA, SVP, TM3/SOC1 and TM8. The phylogenetic tree was constructed by MEGA 7 [33] with the NJ method based on alignments by MAFFT 7 [34]. The phylogenetic tree containing bootstrap values can be observed in Additional file 6: Figure S4

To understand the classification and evolution of plant MIKC-type genes, we conducted phylogenetic analyses with all 1001 MIKC-type genes found in this study from streptophyte lineages. Based on phylogenetic reconstructions of MIKC-type members from alignments of full-length MIKC-type protein sequences, they were classified into the MIKC* and MIKC^C^ groups (Additional file 5: Figure S3). The MIKC^C^ group was further divided into 13 major subfamilies, namely AGL6, AGL12, AGL15, AGL17, SQUA, DEF/GLO, AG, SEP, TM3/SOC1, BS, SVP, FLC and TM8 (Fig. 2; Additional file 6: Figure S4), in agreement with previous findings [15]. Only a single MADS-box gene (GAQ89767.1) was found in the charophyte *K*. *nitens*, which falls into the SVP clade, with weak bootstrap support (34%) by phylogenetic analysis (Additional file 7: Figure S5). Moreover, only one MIKC^C^ gene in the liverwort falls into the SVP subfamily (Additional file 8: Figure S6). This indicates that the ancestral homologs of the SVP gene evolved before the divergence of the charophyte algae lineages from land plant lineages and suggests that the ancestral charophyte emerged with one or only a few MIKC^C^ genes, and that MIKC proteins of all modern land plants descended and radiated from these predecessors by gene duplications, as was suggested by Tanabe et al. [3].

**Fig. 3:**
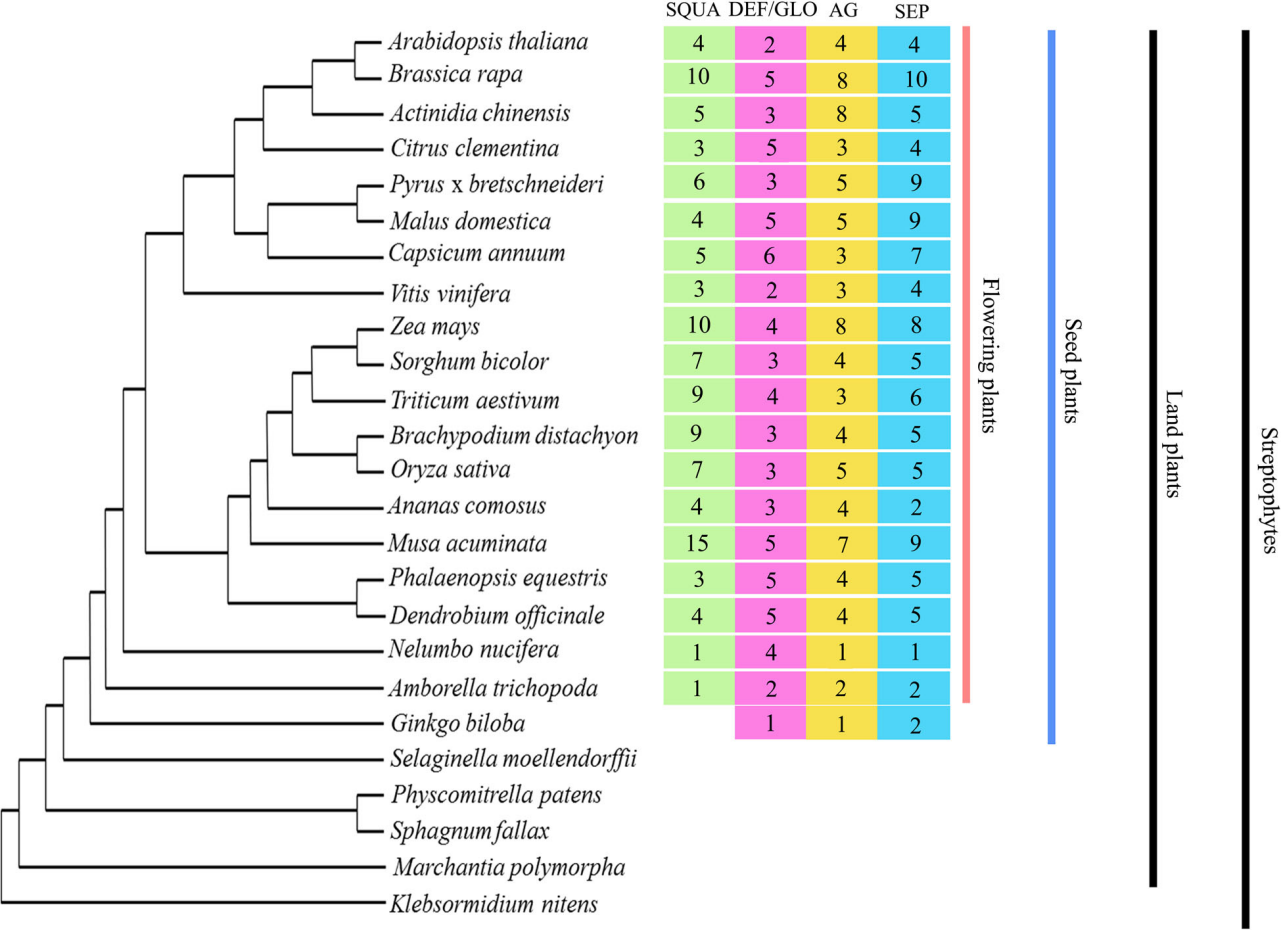
The corresponding number of SQUA, DEF/GLO, AG and SEP subfamily MADS-box genes for each streptophyta lineage identified in this study. The tree topology reflects a consensus of streptophyta phylogenies [32]

Among the 13 subfamilies, SQUA (A class), DEF/GLO (B class), AG (C/D class) and SEP (E class) subfamily genes are well known for their critical roles in flower development [35]. The evolution of the flower itself is suggested to be connected to the evolution of corresponding lineages. As expected, the plant taxonomic groups from charophytes, through to bryophytes and lycophytes, have no SQUA, DEF/GLO, AG and SEP subfamily genes, which are present in all flowering plants, while the gymnosperm *Ginkgo*, which has no flowers, only contains DEF/GLO, AG and SEP subfamily genes (Fig. 3).

### Phylogenetic analysis of MIKC-type genes in orchids

**Fig. 4:**
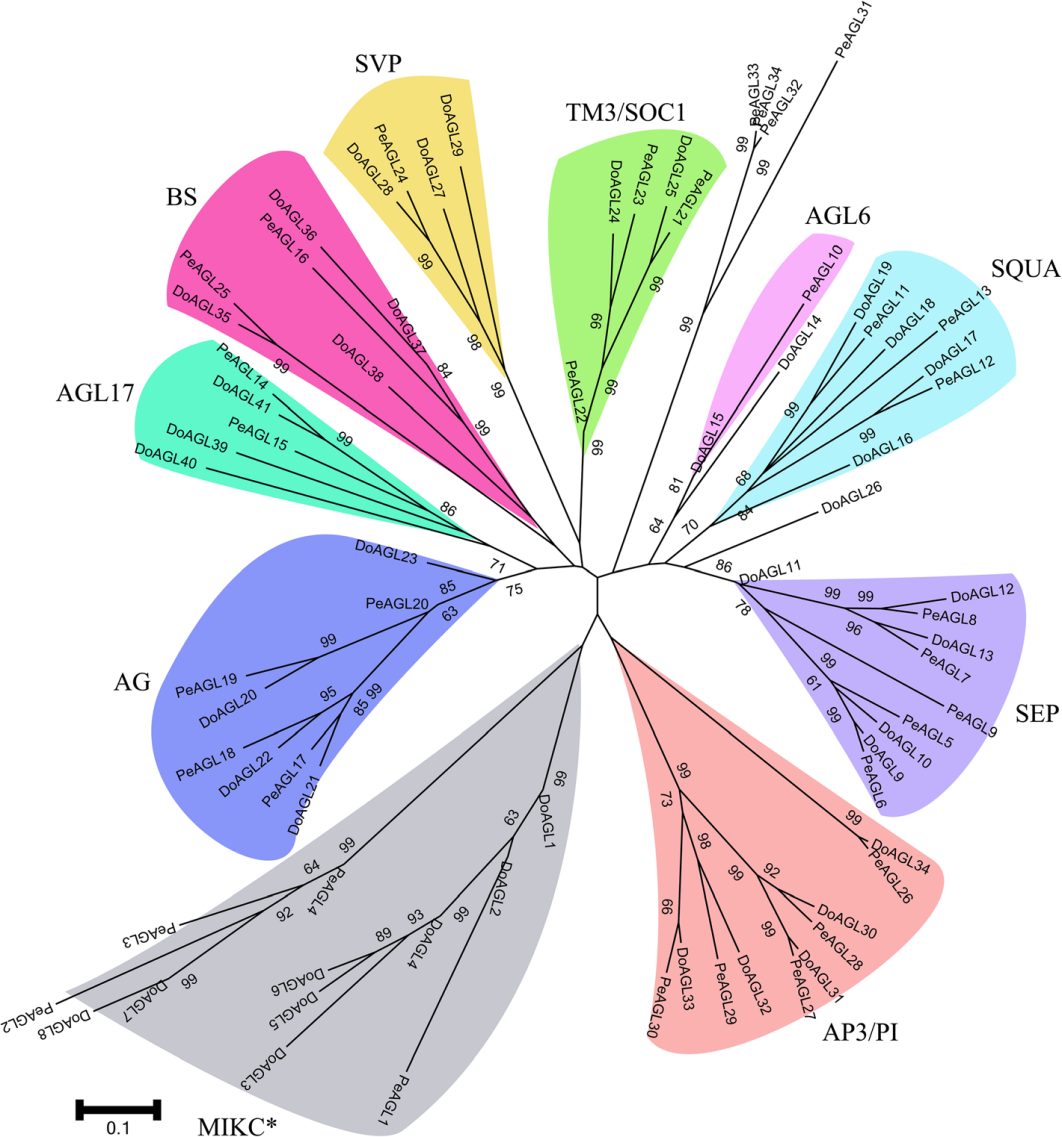
Phylogenetic analyses of MADS-box genes from two orchids, *Dendrobium officinale* and *Phalaenopsis equestris*. The AGL12, AGL15 and FLC subfamilies were lost from the two orchids. The phylogenetic tree was constructed using MEGA 7 [33] based on the alignment of MADS-box proteins by MAFFT 7 [34] with the NJ method. Numbers besides branches represent bootstrap support values from 1000 replications

Analysis of MIKC-type genes in orchids would help to understand the mechanisms of flower formation and provide a foundation for analyzing the diversification of flower structure. A total of 41 and 34 MIKC-type genes were identified in *D. officinale* and *P. equestris*, and could be divided into MIKC* and MIKC^C^ groups. There were 33 and 30 MIKC^C^ genes from *D. officinale* and *P. equestris*, respectively. Phylogenetic analysis shows that the MIKC^C^ genes in both orchids could be divided into nine major subfamilies, SQUA, DEF/GLO, AG, BS, SEP, AGL6, AGL17, TM3/SOC1 and SVP (Fig. 4). The number of genes in each subfamily of both orchids is similar. For example, the DEF/GLO, AG, SEP and AGL6 subfamilies contained the same number of genes (5, 4, 5 and 1, respectively) in both orchids. The TM8, FLC, AGL12 and AGL15 genes were not found in *D. officinale* or *P. equestris*. Other monocots such as rice and *B. distachyon* had no FLC members or AG15 subfamily members but contained AGL12 subfamily genes [13, 14].

### Characterization of ABCE genes in two orchids

Genetic and molecular studies in core eudicots and monocots, as well as basal angiosperms, support a strong correlation between the expression pattern and function of ABCE class genes [17, 20, 36–40]. It is well known that ABCE function genes play a key role in the identity floral organs, including sepals, petals, and stamens, which in orchids differ considerably from other plants. By comparing and analyzing the expression of ABCE genes in two important orchid genera would help to understand the divergence of their gene function.

**Fig. 5:**
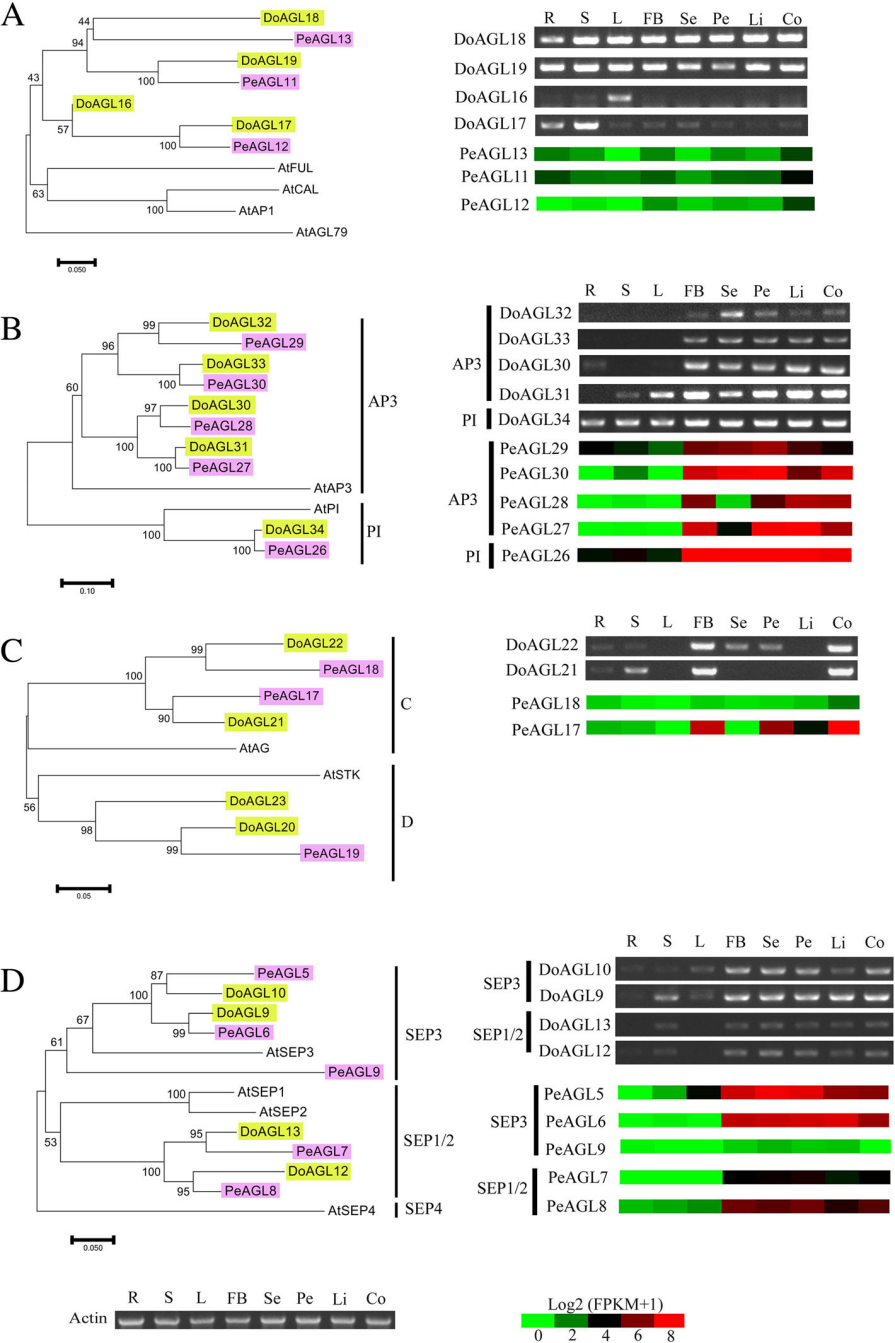
Phylogenetic relationships and expression pattern of SQUA (**a**), DEF/GLO (**b**), AG (**c**) and SEP (**d**) subfamily proteins from *Dendrobium officinale* (Do), *Phalaenopsis equestris* (Pe) and *Arabidopsis thaliana* (At). The phylogenetic tree was constructed using MEGA 7 [33] with the NJ method and 1000 bootstrap replications based on the alignment of MADS-box proteins by MAFFT 7 [34]. R, roots; S, stems; L, leaves; FB, flower buds; Se, sepals; Pe, petals; Li, lips; Co, columns

A phylogenetic reconstruction shows that the *SQUA* genes could be divided into dicot and monocot clades (Fig. 5A). All orchid *SQUA* genes did not display flower-specific expression patterns, and in some cases (*PeAGL11*–*13*, *DoAGL16*, *DoAGL17* and *DoAGL19*), they even had relatively lower expression in sepals (Fig. 5A). The *SQUA* genes (*PeAGL11*–*13*) in *P. equestris* had a relatively low level of expression in all the organs and relatively strong expression in the column (Fig. 5A). *DoAGL18* and *DoAGL19* were detected in all organs and were strongly expressed in stems and leaves (Fig. 5A). *DoAGL16* was only detected in leaves and *DoAGL17* was strongly expressed in roots and stems (Fig. 5A).

The B class genes are required for the establishment of petal and stamen identity. AP3 and PI homologues were recognized as two subclades of B-function genes. Only one PI homologue gene each was found in *D. officinale* and *P. equestris*, while at least four AP3 homologues were present in the two orchids (Fig. 5B). The PI homologues *DoAGL34* and *PeAGL26* were expressed in all four floral organs (sepals, petals, lips and the column) as well as in vegetative organs (roots, stems and leaves) with a similar expression pattern (Fig. 5B). Similarly, the PI homologue *OMADS3* from *Oncidium* Gower Ramsey was expressed in all four floral organs as well as in vegetative tissue (leaves) [26]. The AP3 subclade genes were specific to flowers and displayed similar expression patterns in both orchids. This reveals a possible positive role for AP3 genes in the regulation of sepal, petal, lip and column formation in orchids.

*DoAGL21* and *DoAGL22* from *D. officinale*, while *PeAGL17* and *PeAGL18* were identified as AG-lineage genes from *P. equestris* (Fig. 5C). *DoAGL21* was expressed only in the column and floral buds but *DoAGL22* and *PeAGL17* were expressed in petals and predominantly in the column (Fig. 5C). Consistent with the requirement of AG lineages for normal stamen development [41], AG was expressed in the stamens in both orchids. We suggest that the C function genes are highly conserved among orchids and play an essential role in the regulation of stamen formation.

In this study, the SEP subfamily could be divided into two clades in orchids, SEP1/2 and SEP3 (Fig. 5D). SEP genes *DoAGL9*–*13* from *D. officinale* were detected in roots, stems, and leaves of the reproductive stage and in flowers, with strong expression in floral buds, sepals, petals, lips and the column (Fig. 5D). *DoAGL10* was detected in floral buds, sepals, petals, lips and the column, although it showed relatively lower expression in lips than in sepals, petals, and the column (Fig. 5D). The four SEP subfamily genes (*PeAGL5–8*) from *P. equestris* were expressed in all floral organs, with relatively higher expression in flower buds, sepals and petals than in the lips and stamen (Fig. 5D). The other two *SEP* genes (*DoAGL9* and *DoAGL10*) belonging to the SEP1/2 clade and with a similar expression pattern, were detected in stems and floral organs (Fig. 5D). This result indicates that *DoAGL9* and *DoAGL10* possibly oversee similar biological functions.

### Expression analysis of *D. officinale* MIKC-type genes under cold stress

**Fig. 6:**
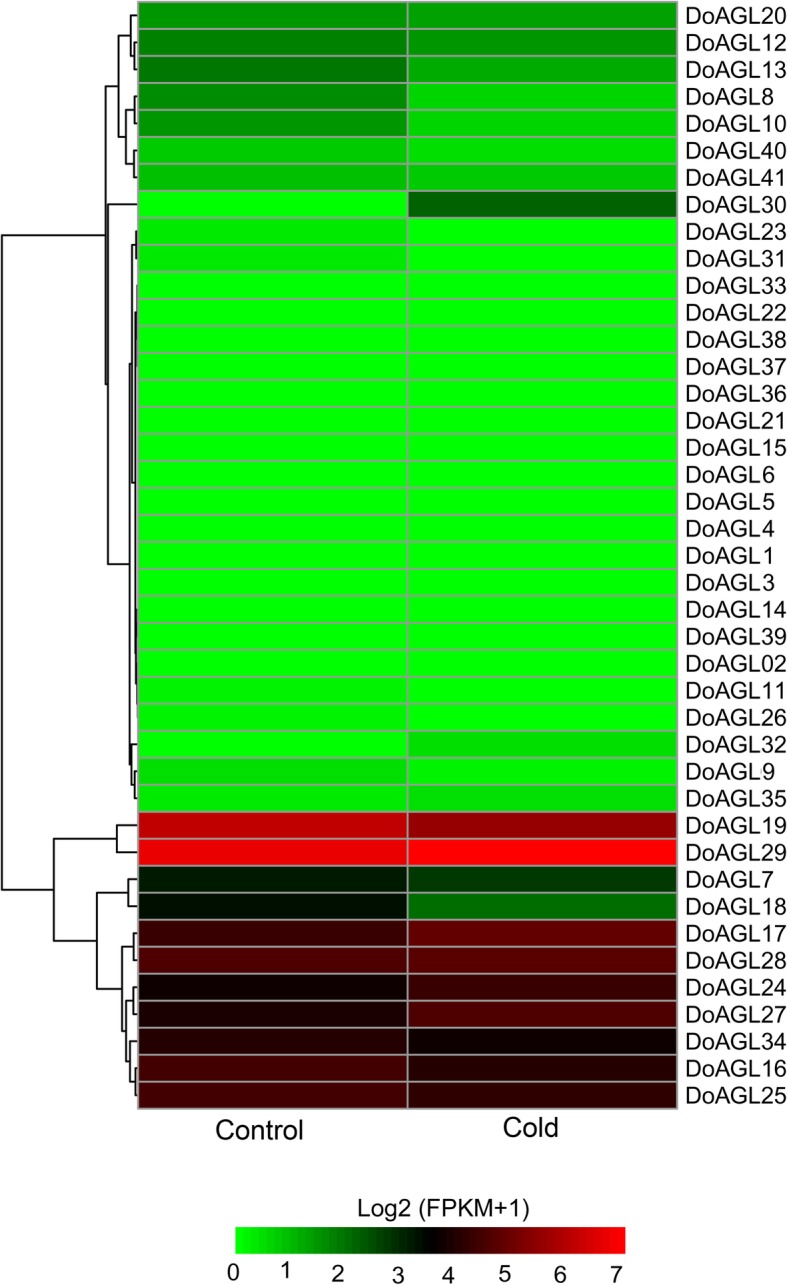
Heat map showing expression pattern of MIKC genes in leaves of *D. officinale* under cold (4 °C) stress

Some clades of MADS-box genes can be induced by low temperatures in tomato (*Solanum lycopersicum*) [42] and rice [14]. This spurred us to characterize the expression patterns of the MIKC-type genes in *D. officinale*. To analyze the expression of *DoAGL*s under cold stress, transcriptome sequencing data of *D. officinale* leaves were downloaded from the SRA database and used to calculated gene expression levels. Only three MIKC-type genes from the SUQA, SOC1 and DEF/GLO clades showed a cold-responsive expression pattern (Fig. 6), *DoAGL18* in the SUQA subfamily was down-regulated 0.358-fold, while its homologs *TM4* in tomato [42], and *LcMADS1* and *LcMADS2* in sheepgrass (*Leymus chinensis*) [43] exhibited an opposite expression pattern, being up-regulated by low temperatures. Two genes, *DoAGL24* in the SOC1 subfamily and *DoAGL27* in the DEF/GLO subfamily, were up-regulated 1.51- and 1.60-fold, respectively more than the control. This result is consistent with DEF/GLO lineages *LcMADS5* from sheepgrass [44] and *TM6* from tomato [42] which were up-regulated in response to cold stress. In *Brassica rapa*, five *SOC1* homologss (*BrMADS36*, − *38*, − *39*, − *40* and − *44*) were induced by cold stress [45]. This result suggests that some MIKC-type genes may play a role in the response of *D. officinale* to low temperature stress while the genes inducible by cold stress are diverse across plant species. The expression of eight MIKC-type genes (*DoAGL07*, − *16*, − *17*, − *19*, − *25*, − *28*, − *29* and *DoAGL34*) was not different with the control under cold stress (Additional file 9: Table S3). Finally, the remaining 30 genes displayed very low expression or were not detected by RNA-seq (Additional file 9: Table S3).

### Transcript analysis of *D. officinale* MIKC genes during seed germination

**Fig. 7:**
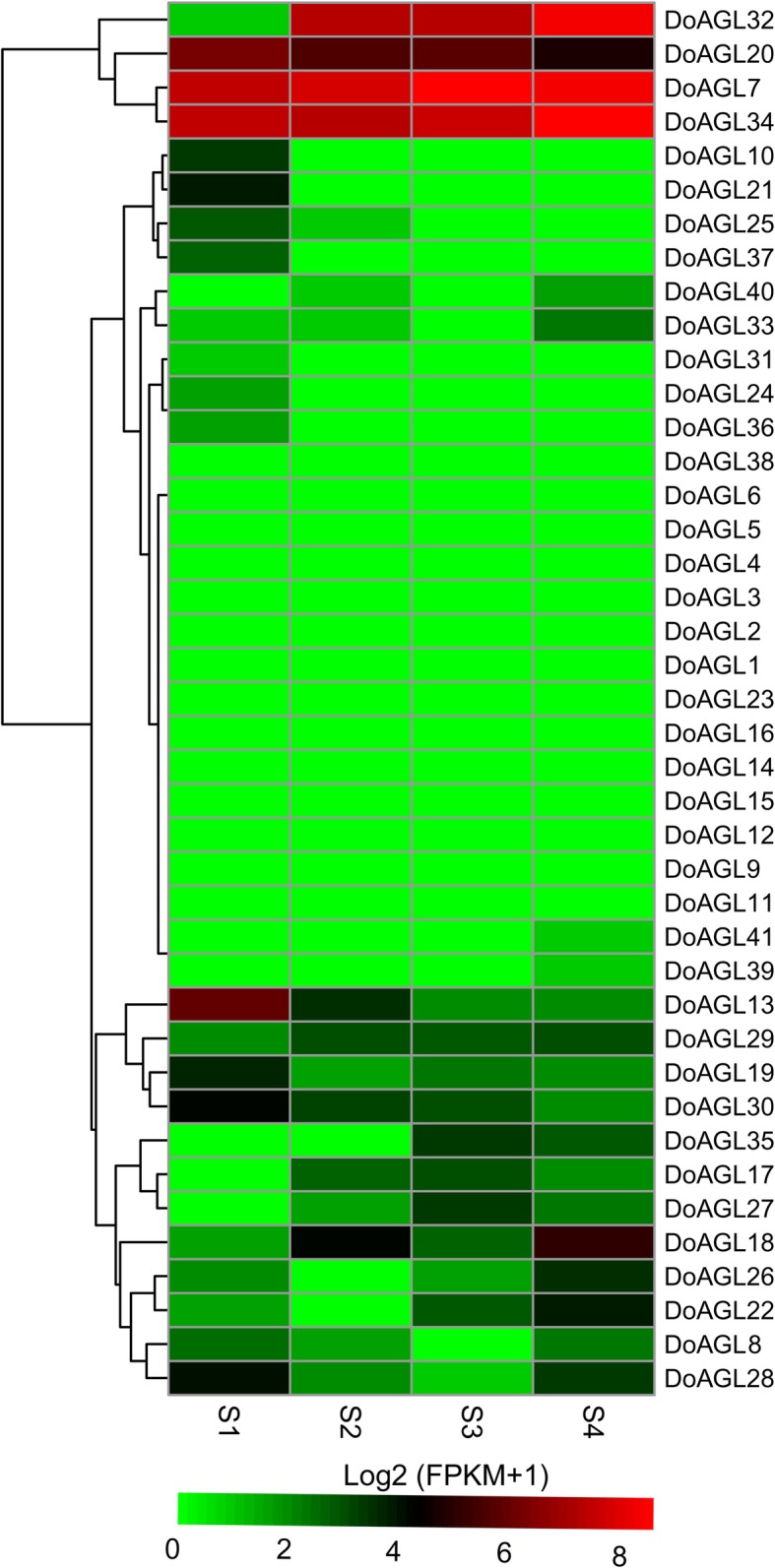
Overview of expression pattern of MIKC genes during the seed germination in *D. officinale*. For more detail of seed germination stages, see the materials and methods section

While the involvement of MIKC genes in the regulation of floral organ development has been well established, their involvement in seed germination is largely unknown. Only two MIKC genes *FLC*/*AGL25* and *AGL2*1in *A. thaliana* were shown to be involved in seed germination by influencing the ABA catabolic pathway and regulating ABA signaling [46, 47]. However, no FLC subfamily genes have been found in orchids, such as *Apostasia odorata* [48], *P. equestris* and *D. officinale* (Fig. 4). This indicates that the manner in which MIKC genes regulate seed germination in orchids may differ from *A. thaliana*. Based on this, the expression of MIKC genes in *D. officinale* during four seed germination stages were analyzed. Six MIKC genes (*DoAGL10*, − *13*, − *19*, − *21*, − *25*, *30* and − *37*) were down-regulated while four MIKC genes (*DoAGL17*, − *18* and − *32*) were up-regulated in S2-S4 (Fig. 7; Additional file 10: Table S4). The SVP subfamily gene *DoAGL27* was not detected in S1, but was detected in S2–4, with highest expression in S3 (Fig. 7; Additional file 10: Table S4). S4 is the stage in which leaves first emerge and a seedling is established. Two SQUA subfamily genes *DoAGL17* and *DoAGL18* showed a higher level of expression at S2-S4, while *DoAGL19* expression was down-regulated in S2-S4 (Fig. 7; Additional file 10: Table S4). This suggests diverse functions of SQUA subfamily genes in *D. officinale*. One DEF/GLO clade gene *DoAGL32* had a low level of expression (FPKM = 1) in S1, but showed a high level of expression in S2-S4 (FPKM_S2_ = 154; FPKM_S3_ = 168; FPKM_S4_ = 325), suggesting that *DoAGL32* may play an important role in seed germination. In this study, a MIKC* gene *DoAGL7* showed more than a 1.5-fold change in S3 and S4 than S1 (Fig. 7), suggesting that it may play a role during seed germination.

## Discussion

### Identification and classification of the MADS-box genes in plants

MADS-box genes have been reidentified in several plant species in this study (details are listed in Additional file 4: Table S2). A total of 108 MADS-box genes were identified from *A. thaliana* and 160 from Chinese cabbage (*B. rapa*), agreeing with the number of MADS-box genes in previous reports [9, 10]. Arora et al. [14] reported a genome-wide identification and annotation of rice MADS-box genes using the TIGR pseudomolecules release 4 (released on January 12, 2006) [49]. In our study, a total of 73 MADS-box genes were found in rice using the current genome assembly (MSU Release 7) [50]. Compared with the previous annotation of MADS-box genes in rice, four genes (LOC_Os02g01355, LOC_Os02g01365, LOC_Os06g01890 and LOC_Os11g12360) not previously annotated as MADS-box genes, were identified. LOC_Os02g01355 and LOC_Os02g01365, which shared the same amino acids and are located on the same chromosome, were regarded as redundant duplicate genes, so LOC_Os02g01355 was removed. Moreover, two MADS-box genes (LOC_Os12g31010 and LOC_Os08g20460) present in the previous study were absent from the latest rice peptide file and were thus excluded from our study. In addition, two genes LOC_Os01g68420 and LOC_Os02g01360 that were previously regarded as MADS-box genes, but that had low identity (less than 38%) with other MADS-box genes and have no M domain or K domain after BLAST analysis in the NCBI database, were excluded from this study. Our result provides more accurate and precise information about the MADS-box family in rice that will help to understand the role of this gene family in reproductive development. In maize, 75 MADS-box genes were found in a previous study [12] while 87 were found in our study, expanding the list by 12.

The MADS-box genes identified from plants in this study, except for the chlorophytes and charophytes, could be divided into two distinct types, type I (SRF-like) and type II (MEF2-like), as in animals and fungi [51]. The type II genes were isolated from three charophycean green algae [3]. It was previously proposed that two main lineages (type I and type II) of MADS-box genes already existed before the divergence of plants and animals/fungi [52].

### The diversification of MIKC-type genes in Streptophyte lineages

The TM8 subfamily was present in the gymnosperm *G. biloba*, basal angiosperms *A. trichopoda* and *Nelumbo nucifera*, and some eudicots *Citrus clementina*, *Malus* x *domestica*, *Pyrus* x *bretschneideri* and *V. vinifera*, but absent in eudicots *A. thaliana* and Chinese cabbage (Additional file 1: Table S1), consistent with previous findings in *A. thaliana* [9], Chinese cabbage [10], *V. vinifera* [53], *A. trichopoda* [54] and *G. biloba* [55]. In addition, the monocot *Zea mays* had one exclusive gene, TM8 (GRMZM2G137289_P01; Additional file 1: Table S1). This suggests that the TM8 subfamily may have been lost during the evolution of some monocots and some eudicots. The MIKC* type genes were found in all streptophyte lineages excluding charophyte algae (Additional file 1: Table S1). The liverwort *M. polymorpha*, regarded as the earliest diverging land plant [6], has only a single MIKC* gene. AGL15 subfamily genes were present in bryophytes, lycophytes, a gymnosperm, basal angiosperms and eudicots, but were absent in monocots (Additional file 1: Table S1). This is consistent with a report in which the AGL15 clade genes were found in gymnosperms [56, 57] and eudicots [9], but were absent in monocots [58]. The AGL15 subfamily genes may have been lost during the evolution of monocot lineages. The DEF/GLO genes were found in other gymnosperms such as the conifer *Pinus radiata* [59] and a gnetale *Gnetum gnemon* [60]. SQUA subfamily genes were not found in *Ginkgo* in this study, and they were also not found in previous studies [56, 61]. The SQUA subfamily genes were also absent in two other gymnosperms, *Pinus taeda* and *Pinus sylvestris* [56]. We suggest that the A-function genes were present after the divergence of angiosperms and gymnosperms and that B-, C- and E-function genes arose before the divergence of angiosperms and gymnosperms.

### The functional divergence and conservation of MIKC^C^ genes in orchids

Both AGL12 and FLC control the flowering transition and act as an enhancer and repressor, respectively in *A. thaliana* flowering time. XAL1 (AGL12) plays an important role in flowering transition, and the late-flowering phenotypes of the *xal1* mutants are able to grow under long days [62]. FLC plays a key role in the initiation of flowering in *A. thaliana*, i.e., *flc* mutations have early flowering phenotypes [63]. The homologues of cereals (wheat and barley) *VRN1*, *FT/VRN3*, and *A. thaliana AGL19*, acted as key players in vernalization pathways, while those found in *Dendrobium nobile* were regulated by vernalization [64]. The AGL12 and FLC subfamily genes were absent in these two orchids *D. officinale* and *P. equestris*, indicating that the regulation of genes during the flowering transition in orchids is different to *A. thaliana*. More analyses are needed to confirm that AGL12 and FLC genes have been lost in the genomes of orchids and to determine the conservation and evolutionary importance of these genes.

In dicots such as *A. thaliana* and *A. majus*, the A class genes, which are required for the development of sepals, show highest expression in sepals but their expression is absent in roots, stems and leaves [38, 65]. However, the SQUA subfamily genes in monocots could be detected in vegetative tissues, the leaves. For example, a rice SQUA-like gene *OsMADS18* is widely expressed in roots, leaves, inflorescences, and developing kernels [66]. We suggest, from our study, that SQUA-like genes underwent functional divergence in monocots and dicots, and even in orchids. The C-lineage was well demonstrated in stamen development in plants, displaying exclusive expression in stamens and carpels, such as in *A. thaliana* [67], *Nicotiana tabacum* [68] and *Lilium longiflorum* [69]. Only a single C-function gene was found in *A. thaliana* [67] and *A. majus* [70], which was essential for the specification of reproductive organs. However, at least two AG-lineages were found in orchids and were expressed predominantly in the column. One AG-lineage gene *CeMADS1* from *Cymbidium ensifolium* was expressed exclusively in columns while another, *CeMADS2*, was detected in all floral organs with strong expression in columns, similar to two *D. officinale* AG-lineage genes [71]. Genetic and molecular studies have demonstrated that the SEP subfamily genes (*SEP1*/*2*/*3*/*4*) are required for the specification of identity of all four whorls of floral organs [17]. The flowers of *A. thaliana sep1 sep2 sep3* triple mutant produced flowers without any petals, stamens and carpels, all of which were replaced by sepals, demonstrating that the SEP genes are required for the specification of petals, stamens, and carpels in Arabidopsis [17]. The *SEP* (*PAP2*) gene regulates spikelet meristem identity and development in rice [44]. The function E orthologous genes from *D. officinale* and *P. equestris* and other orchids such as *Oncidium* Gower Ramsey [72] and *Dendrobium crumenatum* [24] were detected in all floral organs and played a conserved role in floral organs development.

### The MIKC-type genes might play a role in seed germination in *D. officinale*

Some MIKC genes from *D*. *offcinale* showed a different expression during the seed germination, including SVP and SQUA subfamily genes, as well as the MIKC* gene. The SVP subfamily gene *PkMADS1* from *Paulownia kawakamii* displayed an important role in shoot morphogenesis [73], suggesting that the SVP gene in *D. officinale* may have a similar function with other SVP clade genes and may play a role in vegetative shoot development. The function of the MIKC* gene is not well defined. Expression analysis provided clues that MIKC* genes play a role in gametophytic generation in *S*. *moellendorffii*, *Funaria hygrometrica* and *Ceratopteris richardii* [74, 75], as well as pollen development in *A. thaliana* [76]. Liu et al. [77] provided evidence that the MIKC* gene in rice was involved in pollen maturation. However, a MIKC* gene showed a up-regulaed expression during seed germination and might play a role this important process in our study.

## Conclusion

Genome-wide identification of 29 plant species allowed for the identification of a total of 1689 MADS-box genes. These MADS-box genes were divided into type I and type II (MIKC-type) clades. MIKC^C^-type genes, belonging to the MIKC-type clade, were divided into at least 13 subfamilies on the basis of their phylogenetic relationships. The SQUA (A class), AP/PI (B class), AG (C/D class) and SEP (E class) gene subfamilies were analyzed among a charophyte, a liverwort, mosses, a lycophyte, a gymnosperm and angiosperms, providing novel information that enriches our understanding of floral organ development and gene evolution. The expression patterns of ABCE genes in two orchids, as well as the expression pattern of cold stress and seed germination stage in *D. officinale*, were analyzed. This information will fortify our understanding of the conservation and divergence of the function of these genes, and allow floral ontogeny, stress response and seed germination in orchids to be further explained.

## Methods

### Plant materials and RNA isolation

*D. officinale* plants used in this study were grown in South China Botanical Garden, Chinese Academy of Sciences, Guangzhou, China. Gene expression analysis was performed on *D. officinale* tissues: roots, stems, leaves, flower buds, lips, petals, sepals and columns. The roots, stems, leaves and floral organs were collected from 10 pots of *D. officinale* in the reproductive stage, then frozen in liquid nitrogen for 5 min and frozen at − 80 °C for RNA isolation as quickly as possible. Total RNA was extracted from various organs with the RNA extraction kit, Column Plant RNAout2.0 (Tiandz, Inc., Beijing, China), according to the operating manual while contaminating DNA was removed by the RNase-Free DNase I Kit (TaKaRa, Dalian, China).

### Identification of MADS-box genes

All the latest peptide files of the 29 species were downloaded from the online database to generate a local protein database (details of the versions and download websites are listed in Additional file 11: Table S5). The SRF-type TF domain model (PF00319) was obtained from the Pfam database (http://pfam.xfam.org/) and was used to build a hidden Markov model (HMM) file by using the HMMER3 software package under default parameters (http://hmmer.janelia.org/). MADS-box genes were identified through the HMM search program by using the HMM file searched against the local protein databases by HMMER3. The identified sequences were confirmed to be MADS-box proteins by annotating as a MADS-box protein either in the Uniprot database (https://www.uniprot.org/) or in the NCBI database. To ensure the accuracy of the results, the chromosomal localizations of all candidate MADS-box genes were checked and redundant sequences with the same chromosome location were removed.

### Multiple sequence alignments

MAFFT version 7 software [34] was used to align multiple sequence of MADS-box proteins with default parameters to generate a multiple sequence alignment file in FASTA format. ClustalX 2.1 software [78] was used to align sequences and recheck the above results.

### Classification of MADS-box genes by phylogenetic analysis

Phylogenetic and molecular evolutionary analyses of the MADS-box proteins were conducted using MEGA version 7 [33] based on the alignment of MADS-box proteins. The NJ method was used to construct phylogenetic trees in this study. To determine statistical reliability, we reconducted a bootstrap NJ tree by ClustalX 2.1 software [78] to recheck the classification of the MADS-box genes. MADS-box genes were classified according to their phylogenetic relationships with the corresponding Arabidopsis MADS-box genes.

### Gene expression analysis

Total RNA reversion was carried out with the GoScript™ Reverse Transcription System (Promega, Madison, WI, USA) according to its protocols. Transcript levels were determined by semi-quantitative PCR using a Taq PCR Master Mix Kit (DreamTaq™ Green PCR Master Mix kit, TaKaRa, Dalian, China). One microliter of cDNA sample (about 400 ng/μL) from the RT reaction was used for PCR, consisting of 95 °C for 2 min, followed by 40 cycles of denaturation at 98 °C (10 s), annealing at 58 °C (30 s), and extension at 72 °C (60 s). Five microliters of PCR product in each reaction was analyzed by electrophoresis on 1% agarose gels. The primers of MADS-box genes were designed using Primer3.0 software (http://bioinfo.ut.ee/primer3-0.4.0/). To make the primers more specific, the sequences excluding highly conserved M domain sequences were used to the design of primers as much as possible. The specific primers of the MADS-box genes are listed in Additional file 12: Table S6. *D. officinale actin* (NCBI accession No. JX294908) was used as the internal control gene.

For expression analysis of SQUA, DEF/GLO, AG and SEP subfamily genes from *P. equestris*, raw sequencing reads of *P. equestris* samples (roots, stems, leaves, flower buds, sepals, petals, lips and columns) were obtained from the NCBI SRA (accession numbers SRR2080194, SRR2080204, SRR2080202, SRR2080200, SRR3602300, SRR3602299, SRR3602277, and SRR3600816) provided by Niu et al. [79]. Low quality reads were excluded from the raw reeds to generate clean reads, which were used to analyze gene expression. All clean reads were mapped with the nucleotide sequences of SQUA, DEF/GLO, AG and SEP subfamily genes using TopHat version 2.0.8 [80]. Gene expression level was calculated by the FPKM method using HTSeq [81].

For gene expression analysis under cold stress, the transcriptome sequencing data of *D*. *offficinale* leaves under cold stress (4 °C) and control (20 °C) (SRA, accession numbers SRR3210613, SRR3210621, SRR3210626, SRR3210630, SRR3210635 and SRR3210636) were downloaded from SRA, as provided by Wu et al. [82]. The raw reeds were cleaned and used to calculate the level of expression under cold stress as described above. Genes with a mean FPKM > 5 in control or treated leaves were used to calculate fold change (mean of FPKM treated leaves / mean of FPKM control leaves). Genes with a ≥ 1.5-fold change were regarded as up-regulated genes, and those with a ≤ 0.66-fold change were defined as down-regulated genes.

During seed germination, raw sequence reads of four stages of *D. officinale* seed germination (S1, SRR1951787; S2, SRR1951788; S3, SRR1951789; S4, SRR1951790) were downloaded to analyze gene expression using the gene expression methods indicated above. SRA data was provided in Chen et al. [83]. Genes with FPKM > 5 in any one of the stages were regarded as valid genes and were used to calculate fold change (FPKM_S2–4_ / FPKM_S1_). Genes with a ≥ 1.5-fold change or with a ≤ 0.66-fold change were defined as genes with different expression. S1 is the stage in which seeds do not germinate and before sowing on half-strength Murashige and Skoog (MS) [84] culture medium; in S2, the embryo enlarges and the testa ruptures; in S3, a protocorm forms; in S4, a seedling is established with the emergence of a first leaf.

## Additional files

Additional file 1: **Table S1.** Information of MADS-box genes in the 29 tested species. (DOCX 153 kb)

Additional file 2: **Figure S1.** BLASTP graphic overview of four MADS-box proteins from chlorophytes based on the National Center for Biotechnology Information (NCBI) database. (A) MADS-box protein (Cre18.g749550.t1.1) from *Chlamydomonas reinhardtii*. (B) and (C) MADS-box proteins (Vocar.0002 s0667.1.p and Vocar.0014 s0224.1.p) from *Volvox carteri*. (D) MADS-box protein (21861) from *Micromonas pusilla* CCMP1545. (DOCX 155 kb)

Additional file 3: **Figure S2.** Alignment of the amino acid sequences of only one MADS-box gene (GAQ89767.1) from *Klebsormidium nitens* and AtSVP (AT2G22540.1) and AtAGL24 (AT4G24540.1) of the SVP subfamily, as well as AtAGL65 (AT1G18750.1) and AtAGL66 (AT1G77980.1) of MIKC* genes from *Arabidopsis thaliana*. The GAQ89767.1 protein contains M-, I, K- and C domains, which are indicated by red, yellow, green and blue boxes, respectively. (DOCX 1228 kb)

Additional file 4: **Table S2.** Information of MADS-box genes in *Arabidopsis thaliana*, *Oryza sativa*, *Brassica rapa*, *Brachypodium distachyon*, *Zea mays*, *Sorghum bicolor*, *Phalaenopsis equestris*, *Dendrobium officinale*, *Physcomitrella patens*, and *Selaginella moellendorffii. (DOCX 17 kb)*

Additional file 5 **Figure S3.** Classification of the total of 983 MIKC-type proteins based on a phylogenetic analysis. MIKC-type proteins can be divided into two main groups MIKC* and MIKCC. The phylogenetic tree was constructed by MEGA 7 with the NJ method based on MAFFT 7-based alignments. (DOCX 767 kb)

Additional file 6: **Figure S4.** Classification of the total 865 MIKCC proteins based on phylogenetic analysis. The phylogenetic tree was conducted using MEGA 7 based on the alignment of MADS-box proteins by MAFFT 7 with the Neighbor-Joining method. Numbers besides branches represent bootstrap support values from 1000 replications. Values lower than 40% are hidden. (DOCX 2621 kb)

Additional file 7: **Figure S5.** Phylogenetic analyses of GAQ89767.1 from *Klebsormidium nitens* and MADS-box proteins from *Arabidopsis thaliana* (At). The phylogenetic tree was conducted using MEGA 7 based on the alignment of MADS-box proteins by MAFFT 7 with the NJ method. Numbers besides branches represent bootstrap support values from 1000 replications. (DOCX 428 kb)

Additional file 8: **Figure S6.** Phylogenetic analyses of the only two MADS-box proteins (Mapoly0011s0161.1.p and Mapoly0174s0011.1.p) from *Marchantia polymorpha* and MADS-box proteins from *Arabidopsis thaliana* (At). Mapoly0011s0161.1.p falls into the SVP subfamily (29% similarity). Mapoly0174s0011.1.p falls into the MIKC* group. The phylogenetic tree was conducted using MEGA 7 based on the alignment of MADS-box proteins by MAFFT 7 with the NJ method. Numbers besides branches represent bootstrap support values from 1000 replications. (DOCX 413 kb)

Additional file 9: **Table S3.** The mean of FPKM value and fold change of MIKC gene under control and cold stress in *D. officinale* leaf. (DOCX 17 kb)

Additional file 10: **Table S4.** FPKM value of the MIKC gene in *Dendrobium officinale* during four seed germination stages. (DOCX 16 kb)

Additional file 11: **Table S5.** The versin of genomic data and download websites. (DOCX 16 kb)

Additional file 12: **Table S6.** The primers used for Semi-quantitative RT-PCR. (DOCX 14 kb)

## Data Availability

All data generated or analysed during this study are included in this published article and its supplementary information files. The datasets used and analysed during the current study are available from the corresponding author on reasonable request.

## Abbreviations

AG: AGAMOUS
FLC: FLOWERING LOCUS C
FPKM: Fragments per kilobase of exon per million fragments mapped
HMM: Hidden Markov Model
NCBI: The National Center for Biotechnology Information
NJ: Neighbor-Joining
RNA-seq: RNA-sequencing
SQUA: SQUAMOSA
STK: SEEDSTICK
TF: Transcription factor
SRA: Sequence read archive
SEP1/2/3/4: SEPALLATA1/2/3/4;
SHP1/2: SHATTERPROOF1/2
AP1/3: APETALA1/3
