# Supplementary material for: Genome-wide identification and classification of MIKC-type MADS-box genes in Streptophyte lineages and expression analyses to reveal their role in seed germination of orchid
Source: BMC Plant Biol. 2019 May 28;19:223. doi: 10.1186/s12870-019-1836-5 (PMC6540398; doi:10.1186/s12870-019-1836-5)
Supplement: Supplementary file 1 — Table S1. Information of MADS-box genes in the 29 tested species. (DOCX 153 kb) [file 12870_2019_1836_MOESM1_ESM.docx]

**Table S1 Information of MADS-box genes in the 29 tested species.**

| **Group** | **Subfamily** | **Locus name** | **Species** | **Clade** |
| --- | --- | --- | --- | --- |
| Type II (MIKC) | AG | evm_27.model.AmTr_v1.0_scaffold00021.296 | *Amborella trichopoda* | basal angiosperm |
| Type II (MIKC) | AG | evm_27.model.AmTr_v1.0_scaffold00071.203 | *Amborella trichopoda* | basal angiosperm |
| Type II (MIKC) | AG | NNU_26658-RA | *Nelumbo nucifera* | basal angiosperm |
| Type II (MIKC) | AG | Achn182651 | *Actinidia chinensis* | eudicot |
| Type II (MIKC) | AG | Achn367721 | *Actinidia chinensis* | eudicot |
| Type II (MIKC) | AG | Achn243501 | *Actinidia chinensis* | eudicot |
| Type II (MIKC) | AG | Achn121201 | *Actinidia chinensis* | eudicot |
| Type II (MIKC) | AG | Achn214461 | *Actinidia chinensis* | eudicot |
| Type II (MIKC) | AG | Achn089771 | *Actinidia chinensis* | eudicot |
| Type II (MIKC) | AG | AtAG | *Arabidopsis thaliana* | eudicot |
| Type II (MIKC) | AG | AtSHP | *Arabidopsis thaliana* | eudicot |
| Type II (MIKC) | AG | AtSHP2 | *Arabidopsis thaliana* | eudicot |
| Type II (MIKC) | AG | AtSTK | *Arabidopsis thaliana* | eudicot |
| Type II (MIKC) | AG | Bra012564 | *Brassica rapa* | eudicot |
| Type II (MIKC) | AG | Bra013364 | *Brassica rapa* | eudicot |
| Type II (MIKC) | AG | Bra003356 | *Brassica rapa* | eudicot |
| Type II (MIKC) | AG | Bra014552 | *Brassica rapa* | eudicot |
| Type II (MIKC) | AG | Bra007419 | *Brassica rapa* | eudicot |
| Type II (MIKC) | AG | Bra004716 | *Brassica rapa* | eudicot |
| Type II (MIKC) | AG | Bra000696 | *Brassica rapa* | eudicot |
| Type II (MIKC) | AG | Bra037895 | *Brassica rapa* | eudicot |
| Type II (MIKC) | AG | CA.PGAv.1.6.scaffold411.1 | *Capsicum annuum* | eudicot |
| Type II (MIKC) | AG | CA.PGAv.1.6.scaffold585.57 | *Capsicum annuum* | eudicot |
| Type II (MIKC) | AG | CA.PGAv.1.6.scaffold100.9 | *Capsicum annuum* | eudicot |
| Type II (MIKC) | AG | Cg2g043950.1 | *Citrus grandis* | eudicot |
| Type II (MIKC) | AG | Cg7g012730.1 | *Citrus grandis* | eudicot |
| Type II (MIKC) | AG | Cg5g025130.1 | *Citrus grandis* | eudicot |
| Type II (MIKC) | AG | XP_008371528.1 | *Malus domestica* | eudicot |
| Type II (MIKC) | AG | XP_008383548.1 | *Malus domestica* | eudicot |
| Type II (MIKC) | AG | XP_017179301.1 | *Malus domestica* | eudicot |
| Type II (MIKC) | AG | XP_017189539.1 | *Malus domestica* | eudicot |
| Type II (MIKC) | AG | NP_001280931.1 | *Malus domestica* | eudicot |
| Type II (MIKC) | AG | XP_018502014.1 | *Pyrus x bretschneideri* | eudicot |
| Type II (MIKC) | AG | XP_018503931.1 | *Pyrus x bretschneideri* | eudicot |
| Type II (MIKC) | AG | XP_018503023.1 | *Pyrus x bretschneideri* | eudicot |
| Type II (MIKC) | AG | XP_018507924.1 | *Pyrus x bretschneideri* | eudicot |
| Type II (MIKC) | AG | XP_018504265.1 | *Pyrus x bretschneideri* | eudicot |
| Type II (MIKC) | AG | GSVIVT01021303001 | *Vitis vinifera* | eudicot |
| Type II (MIKC) | AG | GSVIVT01000802001 | *Vitis vinifera* | eudicot |
| Type II (MIKC) | AG | GSVIVT01025945001 | *Vitis vinifera* | eudicot |
| Type II (MIKC) | AG | Gb_16301 | *Ginkgo biloba* | gymnosperm |
| Type II (MIKC) | AG | Aco009993.1 | *Ananas comosus* | monocot |
| Type II (MIKC) | AG | Aco007999.1 | *Ananas comosus* | monocot |
| Type II (MIKC) | AG | Aco004785.1 | *Ananas comosus* | monocot |
| Type II (MIKC) | AG | Aco011341.1 | *Ananas comosus* | monocot |
| Type II (MIKC) | AG | Bradi2g06330.1 | *Brachypodium distachyon* | monocot |
| Type II (MIKC) | AG | Bradi2g25090.1 | *Brachypodium distachyon* | monocot |
| Type II (MIKC) | AG | Bradi2g32910.1 | *Brachypodium distachyon* | monocot |
| Type II (MIKC) | AG | Bradi4g40350.1 | *Brachypodium distachyon* | monocot |
| Type II (MIKC) | AG | Dof017895 ( DoAGL22 ) | *Dendrobium officinale* | monocot |
| Type II (MIKC) | AG | Dof017353 ( DoAGL21 ) | *Dendrobium officinale* | monocot |
| Type II (MIKC) | AG | Dof003078 ( DoAGL20 ) | *Dendrobium officinale* | monocot |
| Type II (MIKC) | AG | Dof012374 ( DoAGL23 ) | *Dendrobium officinale* | monocot |
| Type II (MIKC) | AG | Ma05_p06360.1 | *Musa acuminata* | monocot |
| Type II (MIKC) | AG | Ma10_p21430.1 | *Musa acuminata* | monocot |
| Type II (MIKC) | AG | Ma06_p16260.1 | *Musa acuminata* | monocot |
| Type II (MIKC) | AG | Ma10_p14260.1 | *Musa acuminata* | monocot |
| Type II (MIKC) | AG | Ma05_p18560.1 | *Musa acuminata* | monocot |
| Type II (MIKC) | AG | Ma10_p11270.1 | *Musa acuminata* | monocot |
| Type II (MIKC) | AG | Ma07_p25120.1 | *Musa acuminata* | monocot |
| Type II (MIKC) | AG | LOC_Os01g10504.1 | *Oryza sativa* | monocot |
| Type II (MIKC) | AG | LOC_Os01g66290.1 | *Oryza sativa* | monocot |
| Type II (MIKC) | AG | LOC_Os05g11380.1 | *Oryza sativa* | monocot |
| Type II (MIKC) | AG | LOC_Os05g11414.1 | *Oryza sativa* | monocot |
| Type II (MIKC) | AG | LOC_Os12g10540.1 | *Oryza sativa* | monocot |
| Type II (MIKC) | AG | LOC110029636 ( PeAGL18 ) | *Phalaenopsis equestris* | monocot |
| Type II (MIKC) | AG | LOC110021270 ( PeAGL17 ) | *Phalaenopsis equestris* | monocot |
| Type II (MIKC) | AG | LOC110021571 ( PeAGL19 ) | *Phalaenopsis equestris* | monocot |
| Type II (MIKC) | AG | LOC110026071 ( PeAGL20 ) | *Phalaenopsis equestris* | monocot |
| Type II (MIKC) | AG | Sb03g002525.1 | *Sorghum bicolor* | monocot |
| Type II (MIKC) | AG | Sb03g042080.1 | *Sorghum bicolor* | monocot |
| Type II (MIKC) | AG | Sb09g006360.1 | *Sorghum bicolor* | monocot |
| Type II (MIKC) | AG | Sb08g006460.1 | *Sorghum bicolor* | monocot |
| Type II (MIKC) | AG | TRIUR3_34584 | *Triticum aestivum* | monocot |
| Type II (MIKC) | AG | TRIUR3_24724 | *Triticum aestivum* | monocot |
| Type II (MIKC) | AG | TRIUR3_28215 | *Triticum aestivum* | monocot |
| Type II (MIKC) | AG | GRMZM2G359952_P01 | *Zea mays* | monocot |
| Type II (MIKC) | AG | GRMZM2G018589_P01 | *Zea mays* | monocot |
| Type II (MIKC) | AG | GRMZM2G052890_P01 | *Zea mays* | monocot |
| Type II (MIKC) | AG | GRMZM2G074691_P01 | *Zea mays* | monocot |
| Type II (MIKC) | AG | GRMZM2G160687_P01 | *Zea mays* | monocot |
| Type II (MIKC) | AG | GRMZM2G010669_P01 | *Zea mays* | monocot |
| Type II (MIKC) | AG | GRMZM2G336111_P01 | *Zea mays* | monocot |
| Type II (MIKC) | AG | GRMZM2G531231_P01 | *Zea mays* | monocot |
| Type II (MIKC) | AGL12 | evm_27.model.AmTr_v1.0_scaffold00071.216 | *Amborella trichopoda* | basal angiosperm |
| Type II (MIKC) | AGL12 | NNU_08544-RA | *Nelumbo nucifera* | basal angiosperm |
| Type II (MIKC) | AGL12 | AtAGL12 | *Arabidopsis thaliana* | eudicot |
| Type II (MIKC) | AGL12 | Bra003919 | *Brassica rapa* | eudicot |
| Type II (MIKC) | AGL12 | Bra007972 | *Brassica rapa* | eudicot |
| Type II (MIKC) | AGL12 | Bra016128 | *Brassica rapa* | eudicot |
| Type II (MIKC) | AGL12 | CA.PGAv.1.6.scaffold102.2 | *Capsicum annuum* | eudicot |
| Type II (MIKC) | AGL12 | Cg5g025050.1 | *Citrus grandis* | eudicot |
| Type II (MIKC) | AGL12 | XP_017181182.1 | *Malus domestica* | eudicot |
| Type II (MIKC) | AGL12 | XP_008383584.1 | *Malus domestica* | eudicot |
| Type II (MIKC) | AGL12 | XP_008378783.1 | *Malus domestica* | eudicot |
| Type II (MIKC) | AGL12 | XP_009379081.1 | *Pyrus x bretschneideri* | eudicot |
| Type II (MIKC) | AGL12 | XP_009362369.1 | *Pyrus x bretschneideri* | eudicot |
| Type II (MIKC) | AGL12 | GSVIVT01025916001 | *Vitis vinifera* | eudicot |
| Type II (MIKC) | AGL12 | Aco007995.1 | *Ananas comosus* | monocot |
| Type II (MIKC) | AGL12 | Aco018015.1 | *Ananas comosus* | monocot |
| Type II (MIKC) | AGL12 | Bradi4g40940.1 | *Brachypodium distachyon* | monocot |
| Type II (MIKC) | AGL12 | Bradi4g40357.1 | *Brachypodium distachyon* | monocot |
| Type II (MIKC) | AGL12 | Bradi5g11270.1 | *Brachypodium distachyon* | monocot |
| Type II (MIKC) | AGL12 | Bradi3g13570.1 | *Brachypodium distachyon* | monocot |
| Type II (MIKC) | AGL12 | Ma07_p03020.1 | *Musa acuminata* | monocot |
| Type II (MIKC) | AGL12 | Ma11_p17800.1 | *Musa acuminata* | monocot |
| Type II (MIKC) | AGL12 | LOC_Os12g10520.1 | *Oryza sativa* | monocot |
| Type II (MIKC) | AGL12 | LOC_Os08g02070.1 | *Oryza sativa* | monocot |
| Type II (MIKC) | AGL12 | Sb08g006430.1 | *Sorghum bicolor* | monocot |
| Type II (MIKC) | AGL12 | Sb06g017660.1 | *Sorghum bicolor* | monocot |
| Type II (MIKC) | AGL12 | Sb07g001250.1 | *Sorghum bicolor* | monocot |
| Type II (MIKC) | AGL12 | TRIUR3_02252 | *Triticum aestivum* | monocot |
| Type II (MIKC) | AGL12 | TRIUR3_13653 | *Triticum aestivum* | monocot |
| Type II (MIKC) | AGL12 | GRMZM2G105387_P01 | *Zea mays* | monocot |
| Type II (MIKC) | AGL12 | GRMZM2G069370_P01 | *Zea mays* | monocot |
| Type II (MIKC) | AGL12 | GRMZM2G117961_P01 | *Zea mays* | monocot |
| Type II (MIKC) | AGL15 | evm_27.model.AmTr_v1.0_scaffold00053.185 | *Amborella trichopoda* | basal angiosperm |
| Type II (MIKC) | AGL15 | NNU_22830-RA | *Nelumbo nucifera* | basal angiosperm |
| Type II (MIKC) | AGL15 | Sphfalx0133s0037.1.p | *Sphagnum fallax* | bryophyte |
| Type II (MIKC) | AGL15 | Achn112721 | *Actinidia chinensis* | eudicot |
| Type II (MIKC) | AGL15 | AtAGL15 | *Arabidopsis thaliana* | eudicot |
| Type II (MIKC) | AGL15 | AtAGL18 | *Arabidopsis thaliana* | eudicot |
| Type II (MIKC) | AGL15 | Bra005545 | *Brassica rapa* | eudicot |
| Type II (MIKC) | AGL15 | Bra040348 | *Brassica rapa* | eudicot |
| Type II (MIKC) | AGL15 | Bra003278 | *Brassica rapa* | eudicot |
| Type II (MIKC) | AGL15 | Bra019018 | *Brassica rapa* | eudicot |
| Type II (MIKC) | AGL15 | Bra007324 | *Brassica rapa* | eudicot |
| Type II (MIKC) | AGL15 | Bra014628 | *Brassica rapa* | eudicot |
| Type II (MIKC) | AGL15 | Bra006214 | *Brassica rapa* | eudicot |
| Type II (MIKC) | AGL15 | Bra008802 | *Brassica rapa* | eudicot |
| Type II (MIKC) | AGL15 | CA.PGAv.1.6.scaffold1665.3 | *Capsicum annuum* | eudicot |
| Type II (MIKC) | AGL15 | Cg9g020190.1 | *Citrus grandis* | eudicot |
| Type II (MIKC) | AGL15 | Cg7g022840.1 | *Citrus grandis* | eudicot |
| Type II (MIKC) | AGL15 | XP_008351603.1 | *Malus domestica* | eudicot |
| Type II (MIKC) | AGL15 | XP_008392265.1 | *Malus domestica* | eudicot |
| Type II (MIKC) | AGL15 | XP_008346945.1 | *Malus domestica* | eudicot |
| Type II (MIKC) | AGL15 | XP_008349472.1 | *Malus domestica* | eudicot |
| Type II (MIKC) | AGL15 | XP_009363197.1 | *Pyrus x bretschneideri* | eudicot |
| Type II (MIKC) | AGL15 | XP_009335208.1 | *Pyrus x bretschneideri* | eudicot |
| Type II (MIKC) | AGL15 | XP_009368055.1 | *Pyrus x bretschneideri* | eudicot |
| Type II (MIKC) | AGL15 | XP_009354365.1 | *Pyrus x bretschneideri* | eudicot |
| Type II (MIKC) | AGL15 | GSVIVT01033253001 | *Vitis vinifera* | eudicot |
| Type II (MIKC) | AGL15 | GSVIVT01001437001 | *Vitis vinifera* | eudicot |
| Type II (MIKC) | AGL15 | Gb_03807 | *Ginkgo biloba* | gymnosperm |
| Type II (MIKC) | AGL15 | EFJ10734.1 | *Selaginella moellendorffii* | lycophyte |
| Type II (MIKC) | AGL15 | EFJ07529.1 | *Selaginella moellendorffii* | lycophyte |
| Type II (MIKC) | AGL17 | evm_27.model.AmTr_v1.0_scaffold00046.134 | *Amborella trichopoda* | basal angiosperm |
| Type II (MIKC) | AGL17 | NNU_05222-RA | *Nelumbo nucifera* | basal angiosperm |
| Type II (MIKC) | AGL17 | NNU_21145-RA | *Nelumbo nucifera* | basal angiosperm |
| Type II (MIKC) | AGL17 | Achn222091 | *Actinidia chinensis* | eudicot |
| Type II (MIKC) | AGL17 | Achn334061 | *Actinidia chinensis* | eudicot |
| Type II (MIKC) | AGL17 | Achn041761 | *Actinidia chinensis* | eudicot |
| Type II (MIKC) | AGL17 | Achn088351 | *Actinidia chinensis* | eudicot |
| Type II (MIKC) | AGL17 | Achn268161 | *Actinidia chinensis* | eudicot |
| Type II (MIKC) | AGL17 | Achn147701 | *Actinidia chinensis* | eudicot |
| Type II (MIKC) | AGL17 | AtAGL16 | *Arabidopsis thaliana* | eudicot |
| Type II (MIKC) | AGL17 | AtAGL44 | *Arabidopsis thaliana* | eudicot |
| Type II (MIKC) | AGL17 | AtAGL17 | *Arabidopsis thaliana* | eudicot |
| Type II (MIKC) | AGL17 | AtAGL21 | *Arabidopsis thaliana* | eudicot |
| Type II (MIKC) | AGL17 | Bra011509 | *Brassica rapa* | eudicot |
| Type II (MIKC) | AGL17 | Bra017638 | *Brassica rapa* | eudicot |
| Type II (MIKC) | AGL17 | Bra039921 | *Brassica rapa* | eudicot |
| Type II (MIKC) | AGL17 | Bra030222 | *Brassica rapa* | eudicot |
| Type II (MIKC) | AGL17 | Bra010623 | *Brassica rapa* | eudicot |
| Type II (MIKC) | AGL17 | Bra011797 | *Brassica rapa* | eudicot |
| Type II (MIKC) | AGL17 | CA.PGAv.1.6.scaffold1104.14 | *Capsicum annuum* | eudicot |
| Type II (MIKC) | AGL17 | CA.PGAv.1.6.scaffold1061.37 | *Capsicum annuum* | eudicot |
| Type II (MIKC) | AGL17 | Cg8g001950.1 | *Citrus grandis* | eudicot |
| Type II (MIKC) | AGL17 | Cg1g007570.1 | *Citrus grandis* | eudicot |
| Type II (MIKC) | AGL17 | Cg1g007580.1 | *Citrus grandis* | eudicot |
| Type II (MIKC) | AGL17 | XP_017183668.1 | *Malus domestica* | eudicot |
| Type II (MIKC) | AGL17 | XP_008373224.1 | *Malus domestica* | eudicot |
| Type II (MIKC) | AGL17 | XP_008390636.1 | *Malus domestica* | eudicot |
| Type II (MIKC) | AGL17 | XP_017183859.1 | *Malus domestica* | eudicot |
| Type II (MIKC) | AGL17 | XP_017178179.1 | *Malus domestica* | eudicot |
| Type II (MIKC) | AGL17 | XP_009366357.1 | *Pyrus x bretschneideri* | eudicot |
| Type II (MIKC) | AGL17 | XP_009378894.1 | *Pyrus x bretschneideri* | eudicot |
| Type II (MIKC) | AGL17 | XP_018505179.1 | *Pyrus x bretschneideri* | eudicot |
| Type II (MIKC) | AGL17 | XP_018500137.1 | *Pyrus x bretschneideri* | eudicot |
| Type II (MIKC) | AGL17 | XP_018505141.1 | *Pyrus x bretschneideri* | eudicot |
| Type II (MIKC) | AGL17 | XP_018507914.1 | *Pyrus x bretschneideri* | eudicot |
| Type II (MIKC) | AGL17 | XP_018505527.1 | *Pyrus x bretschneideri* | eudicot |
| Type II (MIKC) | AGL17 | XP_009345856.1 | *Pyrus x bretschneideri* | eudicot |
| Type II (MIKC) | AGL17 | XP_018504789.1 | *Pyrus x bretschneideri* | eudicot |
| Type II (MIKC) | AGL17 | XP_018506243.1 | *Pyrus x bretschneideri* | eudicot |
| Type II (MIKC) | AGL17 | GSVIVT01038474001 | *Vitis vinifera* | eudicot |
| Type II (MIKC) | AGL17 | GSVIVT01003861001 | *Vitis vinifera* | eudicot |
| Type II (MIKC) | AGL17 | GSVIVT01003864001 | *Vitis vinifera* | eudicot |
| Type II (MIKC) | AGL17 | GSVIVT01009219001 | *Vitis vinifera* | eudicot |
| Type II (MIKC) | AGL17 | Gb_03068 | *Ginkgo biloba* | gymnosperm |
| Type II (MIKC) | AGL17 | Aco019842.1 | *Ananas comosus* | monocot |
| Type II (MIKC) | AGL17 | Aco001069.1 | *Ananas comosus* | monocot |
| Type II (MIKC) | AGL17 | Aco014671.1 | *Ananas comosus* | monocot |
| Type II (MIKC) | AGL17 | Aco003018.1 | *Ananas comosus* | monocot |
| Type II (MIKC) | AGL17 | Bradi3g46920.1 | *Brachypodium distachyon* | monocot |
| Type II (MIKC) | AGL17 | Bradi5g12440.1 | *Brachypodium distachyon* | monocot |
| Type II (MIKC) | AGL17 | Bradi3g57017.1 | *Brachypodium distachyon* | monocot |
| Type II (MIKC) | AGL17 | Dof018003 ( DoAGL41 ) | *Dendrobium officinale* | monocot |
| Type II (MIKC) | AGL17 | Dof002136 ( DoAGL39 ) | *Dendrobium officinale* | monocot |
| Type II (MIKC) | AGL17 | Dof002134 ( DoAGL40 ) | *Dendrobium officinale* | monocot |
| Type II (MIKC) | AGL17 | Ma03_p07260.1 | *Musa acuminata* | monocot |
| Type II (MIKC) | AGL17 | Ma09_p14960.1 | *Musa acuminata* | monocot |
| Type II (MIKC) | AGL17 | Ma05_p26810.1 | *Musa acuminata* | monocot |
| Type II (MIKC) | AGL17 | Ma07_p12420.1 | *Musa acuminata* | monocot |
| Type II (MIKC) | AGL17 | Ma03_p21090.1 | *Musa acuminata* | monocot |
| Type II (MIKC) | AGL17 | Ma10_p29960.1 | *Musa acuminata* | monocot |
| Type II (MIKC) | AGL17 | LOC_Os04g23910.1 | *Oryza sativa* | monocot |
| Type II (MIKC) | AGL17 | LOC_Os06g23950.1 | *Oryza sativa* | monocot |
| Type II (MIKC) | AGL17 | LOC_Os04g38770.1 | *Oryza sativa* | monocot |
| Type II (MIKC) | AGL17 | LOC_Os02g36924.1 | *Oryza sativa* | monocot |
| Type II (MIKC) | AGL17 | LOC_Os02g49840.1 | *Oryza sativa* | monocot |
| Type II (MIKC) | AGL17 | LOC_Os08g33488.1 | *Oryza sativa* | monocot |
| Type II (MIKC) | AGL17 | LOC110019037 ( PeAGL14 ) | *Phalaenopsis equestris* | monocot |
| Type II (MIKC) | AGL17 | LOC110027541 ( PeAGL15 ) | *Phalaenopsis equestris* | monocot |
| Type II (MIKC) | AGL17 | Sb07g021110.1 | *Sorghum bicolor* | monocot |
| Type II (MIKC) | AGL17 | Sb06g019040.1 | *Sorghum bicolor* | monocot |
| Type II (MIKC) | AGL17 | Sb04g024010.1 | *Sorghum bicolor* | monocot |
| Type II (MIKC) | AGL17 | Sb04g028960.1 | *Sorghum bicolor* | monocot |
| Type II (MIKC) | AGL17 | TRIUR3_09108 | *Triticum aestivum* | monocot |
| Type II (MIKC) | AGL17 | TRIUR3_17292 | *Triticum aestivum* | monocot |
| Type II (MIKC) | AGL17 | TRIUR3_09267 | *Triticum aestivum* | monocot |
| Type II (MIKC) | AGL17 | TRIUR3_09266 | *Triticum aestivum* | monocot |
| Type II (MIKC) | AGL17 | TRIUR3_17291 | *Triticum aestivum* | monocot |
| Type II (MIKC) | AGL17 | TRIUR3_19891 | *Triticum aestivum* | monocot |
| Type II (MIKC) | AGL17 | TRIUR3_08704 | *Triticum aestivum* | monocot |
| Type II (MIKC) | AGL17 | AC212823.4_FGP003 | *Zea mays* | monocot |
| Type II (MIKC) | AGL17 | GRMZM2G302905_P01 | *Zea mays* | monocot |
| Type II (MIKC) | AGL17 | GRMZM2G052045_P01 | *Zea mays* | monocot |
| Type II (MIKC) | AGL17 | GRMZM2G316366_P01 | *Zea mays* | monocot |
| Type II (MIKC) | AGL17 | GRMZM2G032905_P01 | *Zea mays* | monocot |
| Type II (MIKC) | AGL17 | GRMZM2G055782_P01 | *Zea mays* | monocot |
| Type II (MIKC) | AGL17 | GRMZM2G044408_P01 | *Zea mays* | monocot |
| Type II (MIKC) | AGL6 | evm_27.model.AmTr_v1.0_scaffold00001.413 | *Amborella trichopoda* | basal angiosperm |
| Type II (MIKC) | AGL6 | NNU_26581-RA | *Nelumbo nucifera* | basal angiosperm |
| Type II (MIKC) | AGL6 | AtAGL13 | *Arabidopsis thaliana* | eudicot |
| Type II (MIKC) | AGL6 | AtAGL6 | *Arabidopsis thaliana* | eudicot |
| Type II (MIKC) | AGL6 | Bra014454 | *Brassica rapa* | eudicot |
| Type II (MIKC) | AGL6 | Bra000392 | *Brassica rapa* | eudicot |
| Type II (MIKC) | AGL6 | Bra004927 | *Brassica rapa* | eudicot |
| Type II (MIKC) | AGL6 | CA.PGAv.1.6.scaffold1561.4 | *Capsicum annuum* | eudicot |
| Type II (MIKC) | AGL6 | Cg5g042960.1 | *Citrus grandis* | eudicot |
| Type II (MIKC) | AGL6 | NP_001280892.1 | *Malus domestica* | eudicot |
| Type II (MIKC) | AGL6 | XP_009375842.1 | *Pyrus x bretschneideri* | eudicot |
| Type II (MIKC) | AGL6 | GSVIVT01018450001 | *Vitis vinifera* | eudicot |
| Type II (MIKC) | AGL6 | Aco015487.1 | *Ananas comosus* | monocot |
| Type II (MIKC) | AGL6 | Bradi3g51800.1 | *Brachypodium distachyon* | monocot |
| Type II (MIKC) | AGL6 | Dof014821 ( DoAGL15 ) | *Dendrobium officinale* | monocot |
| Type II (MIKC) | AGL6 | Ma04_p36630.1 | *Musa acuminata* | monocot |
| Type II (MIKC) | AGL6 | Ma02_p12050.1 | *Musa acuminata* | monocot |
| Type II (MIKC) | AGL6 | Ma04_p20900.1 | *Musa acuminata* | monocot |
| Type II (MIKC) | AGL6 | Ma01_p18390.1 | *Musa acuminata* | monocot |
| Type II (MIKC) | AGL6 | Ma02_p06320.1 | *Musa acuminata* | monocot |
| Type II (MIKC) | AGL6 | LOC_Os02g45770.1 | *Oryza sativa* | monocot |
| Type II (MIKC) | AGL6 | LOC_Os04g49150.1 | *Oryza sativa* | monocot |
| Type II (MIKC) | AGL6 | Sb04g031750.1 | *Sorghum bicolor* | monocot |
| Type II (MIKC) | AGL6 | Sb06g026300.1 | *Sorghum bicolor* | monocot |
| Type II (MIKC) | AGL6 | TRIUR3_22246 | *Triticum aestivum* | monocot |
| Type II (MIKC) | AGL6 | GRMZM2G003514_P01 | *Zea mays* | monocot |
| Type II (MIKC) | AGL6 | GRMZM2G160565_P01 | *Zea mays* | monocot |
| Type II (MIKC) | AGL6 | LOC110023965 ( PeAGL10 ) | *Phalaenopsis equestris* | monocot |
| Type II (MIKC) | BS | evm_27.model.AmTr_v1.0_scaffold00002.466 | *Amborella trichopoda* | basal angiosperm |
| Type II (MIKC) | BS | evm_27.model.AmTr_v1.0_scaffold00001.461 | *Amborella trichopoda* | basal angiosperm |
| Type II (MIKC) | BS | NNU_06346-RA | *Nelumbo nucifera* | basal angiosperm |
| Type II (MIKC) | BS | NNU_14417-RA | *Nelumbo nucifera* | basal angiosperm |
| Type II (MIKC) | BS | Achn199931 | *Actinidia chinensis* | eudicot |
| Type II (MIKC) | BS | Achn263141 | *Actinidia chinensis* | eudicot |
| Type II (MIKC) | BS | AtGOA | *Arabidopsis thaliana* | eudicot |
| Type II (MIKC) | BS | AtAGL32 | *Arabidopsis thaliana* | eudicot |
| Type II (MIKC) | BS | Bra026507 | *Brassica rapa* | eudicot |
| Type II (MIKC) | BS | Bra013028 | *Brassica rapa* | eudicot |
| Type II (MIKC) | BS | Bra029365 | *Brassica rapa* | eudicot |
| Type II (MIKC) | BS | Cg7g013940.1 | *Citrus grandis* | eudicot |
| Type II (MIKC) | BS | Cg2g037530.1 | *Citrus grandis* | eudicot |
| Type II (MIKC) | BS | XP_017180647.1 | *Malus domestica* | eudicot |
| Type II (MIKC) | BS | XP_009353975.1 | *Pyrus x bretschneideri* | eudicot |
| Type II (MIKC) | BS | GSVIVT01026207001 | *Vitis vinifera* | eudicot |
| Type II (MIKC) | BS | GSVIVT01012110001 | *Vitis vinifera* | eudicot |
| Type II (MIKC) | BS | GSVIVT01027577001 | *Vitis vinifera* | eudicot |
| Type II (MIKC) | BS | Aco006017.1 | *Ananas comosus* | monocot |
| Type II (MIKC) | BS | Aco008359.1 | *Ananas comosus* | monocot |
| Type II (MIKC) | BS | Bradi1g32210.1 | *Brachypodium distachyon* | monocot |
| Type II (MIKC) | BS | Bradi2g48690.1 | *Brachypodium distachyon* | monocot |
| Type II (MIKC) | BS | Bradi5g21700.1 | *Brachypodium distachyon* | monocot |
| Type II (MIKC) | BS | Bradi3g05260.1 | *Brachypodium distachyon* | monocot |
| Type II (MIKC) | BS | Dof018107 ( DoAGL35 ) | *Dendrobium officinale* | monocot |
| Type II (MIKC) | BS | Dof023400 ( DoAGL38 ) | *Dendrobium officinale* | monocot |
| Type II (MIKC) | BS | Dof006092 ( DoAGL36 ) | *Dendrobium officinale* | monocot |
| Type II (MIKC) | BS | Dof006090 ( DoAGL37 ) | *Dendrobium officinale* | monocot |
| Type II (MIKC) | BS | Ma09_p05610.1 | *Musa acuminata* | monocot |
| Type II (MIKC) | BS | Ma02_p04560.1 | *Musa acuminata* | monocot |
| Type II (MIKC) | BS | Ma03_p24850.1 | *Musa acuminata* | monocot |
| Type II (MIKC) | BS | LOC_Os06g45650.1 | *Oryza sativa* | monocot |
| Type II (MIKC) | BS | LOC_Os01g52680.1 | *Oryza sativa* | monocot |
| Type II (MIKC) | BS | LOC_Os04g52410.1 | *Oryza sativa* | monocot |
| Type II (MIKC) | BS | LOC_Os02g07430.1 | *Oryza sativa* | monocot |
| Type II (MIKC) | BS | LOC110036579 ( PeAGL25 ) | *Phalaenopsis equestris* | monocot |
| Type II (MIKC) | BS | LOC110028723 ( PeAGL16 ) | *Phalaenopsis equestris* | monocot |
| Type II (MIKC) | BS | Sb10g026690.1 | *Sorghum bicolor* | monocot |
| Type II (MIKC) | BS | Sb03g033380.1 | *Sorghum bicolor* | monocot |
| Type II (MIKC) | BS | Sb06g028420.1 | *Sorghum bicolor* | monocot |
| Type II (MIKC) | BS | Sb04g004736.1 | *Sorghum bicolor* | monocot |
| Type II (MIKC) | BS | TRIUR3_03730 | *Triticum aestivum* | monocot |
| Type II (MIKC) | BS | TRIUR3_18402 | *Triticum aestivum* | monocot |
| Type II (MIKC) | BS | TRIUR3_26896 | *Triticum aestivum* | monocot |
| Type II (MIKC) | BS | GRMZM2G161666_P01 | *Zea mays* | monocot |
| Type II (MIKC) | BS | GRMZM2G038878_P01 | *Zea mays* | monocot |
| Type II (MIKC) | BS | GRMZM2G001139_P01 | *Zea mays* | monocot |
| Type II (MIKC) | BS | GRMZM2G079727_P01 | *Zea mays* | monocot |
| Type II (MIKC) | BS | GRMZM2G137387_P01 | *Zea mays* | monocot |
| Type II (MIKC) | BS | AC233912.1_FGP001 | *Zea mays* | monocot |
| Type II (MIKC) | BS | GRMZM2G128953_P01 | *Zea mays* | monocot |
| Type II (MIKC) | BS | GRMZM2G148220_P01 | *Zea mays* | monocot |
| Type II (MIKC) | BS | AC233785.2_FGP002 | *Zea mays* | monocot |
| Type II (MIKC) | BS | GRMZM2G005155_P01 | *Zea mays* | monocot |
| Type II (MIKC) | BS | GRMZM2G070151_P02 | *Zea mays* | monocot |
| Type II (MIKC) | BS | GRMZM2G135018_P01 | *Zea mays* | monocot |
| Type II (MIKC) | BS | GRMZM2G095415_P01 | *Zea mays* | monocot |
| Type II (MIKC) | BS | GRMZM2G446426_P01 | *Zea mays* | monocot |
| Type II (MIKC) | BS | GRMZM2G049841_P01 | *Zea mays* | monocot |
| Type II (MIKC) | BS | GRMZM2G130382_P01 | *Zea mays* | monocot |
| Type II (MIKC) | DEF/GLO | evm_27.model.AmTr_v1.0_scaffold00066.97 | *Amborella trichopoda* | basal angiosperm |
| Type II (MIKC) | DEF/GLO | evm_27.model.AmTr_v1.0_scaffold00001.225 | *Amborella trichopoda* | basal angiosperm |
| Type II (MIKC) | DEF/GLO | evm_27.model.AmTr_v1.0_scaffold00089.36 | *Amborella trichopoda* | basal angiosperm |
| Type II (MIKC) | DEF/GLO | NNU_23351-RA | *Nelumbo nucifera* | basal angiosperm |
| Type II (MIKC) | DEF/GLO | NNU_15351-RA | *Nelumbo nucifera* | basal angiosperm |
| Type II (MIKC) | DEF/GLO | NNU_08090-RA | *Nelumbo nucifera* | basal angiosperm |
| Type II (MIKC) | DEF/GLO | NNU_02674-RA | *Nelumbo nucifera* | basal angiosperm |
| Type II (MIKC) | DEF/GLO | Achn049281 | *Actinidia chinensis* | eudicot |
| Type II (MIKC) | DEF/GLO | Achn135681 | *Actinidia chinensis* | eudicot |
| Type II (MIKC) | DEF/GLO | Achn201411 | *Actinidia chinensis* | eudicot |
| Type II (MIKC) | DEF/GLO | AtAP3 | *Arabidopsis thaliana* | eudicot |
| Type II (MIKC) | DEF/GLO | AtPI | *Arabidopsis thaliana* | eudicot |
| Type II (MIKC) | DEF/GLO | Bra007067 | *Brassica rapa* | eudicot |
| Type II (MIKC) | DEF/GLO | Bra014822 | *Brassica rapa* | eudicot |
| Type II (MIKC) | DEF/GLO | Bra006549 | *Brassica rapa* | eudicot |
| Type II (MIKC) | DEF/GLO | Bra020093 | *Brassica rapa* | eudicot |
| Type II (MIKC) | DEF/GLO | Bra002285 | *Brassica rapa* | eudicot |
| Type II (MIKC) | DEF/GLO | CA.PGAv.1.6.scaffold851.17 | *Capsicum annuum* | eudicot |
| Type II (MIKC) | DEF/GLO | CA.PGAv.1.6.scaffold358.12 | *Capsicum annuum* | eudicot |
| Type II (MIKC) | DEF/GLO | CA.PGAv.1.6.scaffold3954.1 | *Capsicum annuum* | eudicot |
| Type II (MIKC) | DEF/GLO | CA.PGAv.1.6.scaffold4963.1 | *Capsicum annuum* | eudicot |
| Type II (MIKC) | DEF/GLO | CA.PGAv.1.6.scaffold348.10 | *Capsicum annuum* | eudicot |
| Type II (MIKC) | DEF/GLO | CA.PGAv.1.6.scaffold531.15 | *Capsicum annuum* | eudicot |
| Type II (MIKC) | DEF/GLO | Cg3g023510.1 | *Citrus grandis* | eudicot |
| Type II (MIKC) | DEF/GLO | Cg4g018800.1 | *Citrus grandis* | eudicot |
| Type II (MIKC) | DEF/GLO | Cg1g005070.1 | *Citrus grandis* | eudicot |
| Type II (MIKC) | DEF/GLO | Cg3g017080.1 | *Citrus grandis* | eudicot |
| Type II (MIKC) | DEF/GLO | Cg3g017100.1 | *Citrus grandis* | eudicot |
| Type II (MIKC) | DEF/GLO | XP_008367257.1 | *Malus domestica* | eudicot |
| Type II (MIKC) | DEF/GLO | XP_008344258.1 | *Malus domestica* | eudicot |
| Type II (MIKC) | DEF/GLO | XP_008346633.1 | *Malus domestica* | eudicot |
| Type II (MIKC) | DEF/GLO | XP_008364381.1 | *Malus domestica* | eudicot |
| Type II (MIKC) | DEF/GLO | NP_001280926.1 | *Malus domestica* | eudicot |
| Type II (MIKC) | DEF/GLO | XP_009346296.1 | *Pyrus x bretschneideri* | eudicot |
| Type II (MIKC) | DEF/GLO | XP_009378223.1 | *Pyrus x bretschneideri* | eudicot |
| Type II (MIKC) | DEF/GLO | XP_009358735.1 | *Pyrus x bretschneideri* | eudicot |
| Type II (MIKC) | DEF/GLO | GSVIVT01009815001 | *Vitis vinifera* | eudicot |
| Type II (MIKC) | DEF/GLO | GSVIVT01008806001 | *Vitis vinifera* | eudicot |
| Type II (MIKC) | DEF/GLO | Gb_15398 | *Ginkgo biloba* | gymnosperm |
| Type II (MIKC) | DEF/GLO | Aco017589.1 | *Ananas comosus* | monocot |
| Type II (MIKC) | DEF/GLO | Aco025594.1 | *Ananas comosus* | monocot |
| Type II (MIKC) | DEF/GLO | Aco019365.1 | *Ananas comosus* | monocot |
| Type II (MIKC) | DEF/GLO | Bradi1g35000.1 | *Brachypodium distachyon* | monocot |
| Type II (MIKC) | DEF/GLO | Bradi2g24940.2 | *Brachypodium distachyon* | monocot |
| Type II (MIKC) | DEF/GLO | Bradi2g57000.1 | *Brachypodium distachyon* | monocot |
| Type II (MIKC) | DEF/GLO | Dof005941 ( DoAGL30 ) | *Dendrobium officinale* | monocot |
| Type II (MIKC) | DEF/GLO | Dof012472 ( DoAGL31 ) | *Dendrobium officinale* | monocot |
| Type II (MIKC) | DEF/GLO | Dof018192 ( DoAGL32 ) | *Dendrobium officinale* | monocot |
| Type II (MIKC) | DEF/GLO | Dof019113 ( DoAGL33 ) | *Dendrobium officinale* | monocot |
| Type II (MIKC) | DEF/GLO | Dof000690 ( DoAGL34 ) | *Dendrobium officinale* | monocot |
| Type II (MIKC) | DEF/GLO | Ma09_p21260.1 | *Musa acuminata* | monocot |
| Type II (MIKC) | DEF/GLO | Ma01_p11930.1 | *Musa acuminata* | monocot |
| Type II (MIKC) | DEF/GLO | Ma09_p21270.1 | *Musa acuminata* | monocot |
| Type II (MIKC) | DEF/GLO | Ma05_p01150.1 | *Musa acuminata* | monocot |
| Type II (MIKC) | DEF/GLO | Ma06_p20000.1 | *Musa acuminata* | monocot |
| Type II (MIKC) | DEF/GLO | LOC_Os06g49840.1 | *Oryza sativa* | monocot |
| Type II (MIKC) | DEF/GLO | LOC_Os05g34940.1 | *Oryza sativa* | monocot |
| Type II (MIKC) | DEF/GLO | LOC_Os01g66030.1 | *Oryza sativa* | monocot |
| Type II (MIKC) | DEF/GLO | LOC110018630 ( PeAGL27 ) | *Phalaenopsis equestris* | monocot |
| Type II (MIKC) | DEF/GLO | LOC110024739 ( PeAGL29 ) | *Phalaenopsis equestris* | monocot |
| Type II (MIKC) | DEF/GLO | LOC110028792 ( PeAGL30 ) | *Phalaenopsis equestris* | monocot |
| Type II (MIKC) | DEF/GLO | LOC110019973 ( PeAGL26 ) | *Phalaenopsis equestris* | monocot |
| Type II (MIKC) | DEF/GLO | LOC110030201 ( PeAGL28 ) | *Phalaenopsis equestris* | monocot |
| Type II (MIKC) | DEF/GLO | Sb10g029810.1 | *Sorghum bicolor* | monocot |
| Type II (MIKC) | DEF/GLO | Sb09g020770.1 | *Sorghum bicolor* | monocot |
| Type II (MIKC) | DEF/GLO | Sb03g041860.1 | *Sorghum bicolor* | monocot |
| Type II (MIKC) | DEF/GLO | TRIUR3_29719 | *Triticum aestivum* | monocot |
| Type II (MIKC) | DEF/GLO | TRIUR3_33318 | *Triticum aestivum* | monocot |
| Type II (MIKC) | DEF/GLO | TRIUR3_15782 | *Triticum aestivum* | monocot |
| Type II (MIKC) | DEF/GLO | TRIUR3_25027 | *Triticum aestivum* | monocot |
| Type II (MIKC) | DEF/GLO | GRMZM2G139073_P01 | *Zea mays* | monocot |
| Type II (MIKC) | DEF/GLO | GRMZM2G152862_P01 | *Zea mays* | monocot |
| Type II (MIKC) | DEF/GLO | GRMZM2G110153_P01 | *Zea mays* | monocot |
| Type II (MIKC) | DEF/GLO | GRMZM5G805387_P01 | *Zea mays* | monocot |
| Type II (MIKC) | FLC | Achn235371 | *Actinidia chinensis* | eudicot |
| Type II (MIKC) | FLC | Achn324471 | *Actinidia chinensis* | eudicot |
| Type II (MIKC) | FLC | Achn196281 | *Actinidia chinensis* | eudicot |
| Type II (MIKC) | FLC | AtMAF2 | *Arabidopsis thaliana* | eudicot |
| Type II (MIKC) | FLC | AtMAF3 | *Arabidopsis thaliana* | eudicot |
| Type II (MIKC) | FLC | AtMAF4 | *Arabidopsis thaliana* | eudicot |
| Type II (MIKC) | FLC | AtMAF5 | *Arabidopsis thaliana* | eudicot |
| Type II (MIKC) | FLC | AtMAF1 | *Arabidopsis thaliana* | eudicot |
| Type II (MIKC) | FLC | AtFLC | *Arabidopsis thaliana* | eudicot |
| Type II (MIKC) | FLC | Bra031888 | *Brassica rapa* | eudicot |
| Type II (MIKC) | FLC | Bra024350 | *Brassica rapa* | eudicot |
| Type II (MIKC) | FLC | Bra031886 | *Brassica rapa* | eudicot |
| Type II (MIKC) | FLC | Bra024351 | *Brassica rapa* | eudicot |
| Type II (MIKC) | FLC | Bra031884 | *Brassica rapa* | eudicot |
| Type II (MIKC) | FLC | Bra022771 | *Brassica rapa* | eudicot |
| Type II (MIKC) | FLC | Bra009055 | *Brassica rapa* | eudicot |
| Type II (MIKC) | FLC | Bra006051 | *Brassica rapa* | eudicot |
| Type II (MIKC) | FLC | Bra028599 | *Brassica rapa* | eudicot |
| Type II (MIKC) | FLC | CA.PGAv.1.6.scaffold13.15 | *Capsicum annuum* | eudicot |
| Type II (MIKC) | FLC | CA.PGAv.1.6.scaffold683.11 | *Capsicum annuum* | eudicot |
| Type II (MIKC) | FLC | CA.PGAv.1.6.scaffold1122.4 | *Capsicum annuum* | eudicot |
| Type II (MIKC) | FLC | CA.PGAv.1.6.scaffold683.5 | *Capsicum annuum* | eudicot |
| Type II (MIKC) | FLC | CA.PGAv.1.6.scaffold1122.3 | *Capsicum annuum* | eudicot |
| Type II (MIKC) | FLC | CA.PGAv.1.6.scaffold683.4 | *Capsicum annuum* | eudicot |
| Type II (MIKC) | FLC | CA.PGAv.1.6.scaffold683.2 | *Capsicum annuum* | eudicot |
| Type II (MIKC) | FLC | CA.PGAv.1.6.scaffold683.7 | *Capsicum annuum* | eudicot |
| Type II (MIKC) | FLC | Cg7g016790.1 | *Citrus grandis* | eudicot |
| Type II (MIKC) | FLC | XP_018500410.1 | *Pyrus x bretschneideri* | eudicot |
| Type II (MIKC) | FLC | GSVIVT01033067001 | *Vitis vinifera* | eudicot |
| Type II (MIKC) | SEP | evm_27.model.AmTr_v1.0_scaffold00013.53 | *Amborella trichopoda* | basal angiosperm |
| Type II (MIKC) | SEP | evm_27.model.AmTr_v1.0_scaffold00047.121 | *Amborella trichopoda* | basal angiosperm |
| Type II (MIKC) | SEP | NNU_07468-RA | *Nelumbo nucifera* | basal angiosperm |
| Type II (MIKC) | SEP | Achn322871 | *Actinidia chinensis* | eudicot |
| Type II (MIKC) | SEP | Achn301521 | *Actinidia chinensis* | eudicot |
| Type II (MIKC) | SEP | Achn196271 | *Actinidia chinensis* | eudicot |
| Type II (MIKC) | SEP | Achn350701 | *Actinidia chinensis* | eudicot |
| Type II (MIKC) | SEP | Achn252981 | *Actinidia chinensis* | eudicot |
| Type II (MIKC) | SEP | AtSEP4 | *Arabidopsis thaliana* | eudicot |
| Type II (MIKC) | SEP | AtSEP2 | *Arabidopsis thaliana* | eudicot |
| Type II (MIKC) | SEP | AtSEP1 | *Arabidopsis thaliana* | eudicot |
| Type II (MIKC) | SEP | AtSEP3 | *Arabidopsis thaliana* | eudicot |
| Type II (MIKC) | SEP | Bra017376 | *Brassica rapa* | eudicot |
| Type II (MIKC) | SEP | Bra026543 | *Brassica rapa* | eudicot |
| Type II (MIKC) | SEP | Bra025126 | *Brassica rapa* | eudicot |
| Type II (MIKC) | SEP | Bra021470 | *Brassica rapa* | eudicot |
| Type II (MIKC) | SEP | Bra039170 | *Brassica rapa* | eudicot |
| Type II (MIKC) | SEP | Bra008674 | *Brassica rapa* | eudicot |
| Type II (MIKC) | SEP | Bra006322 | *Brassica rapa* | eudicot |
| Type II (MIKC) | SEP | Bra030032 | *Brassica rapa* | eudicot |
| Type II (MIKC) | SEP | Bra010955 | *Brassica rapa* | eudicot |
| Type II (MIKC) | SEP | Bra032814 | *Brassica rapa* | eudicot |
| Type II (MIKC) | SEP | CA.PGAv.1.6.scaffold1448.3 | *Capsicum annuum* | eudicot |
| Type II (MIKC) | SEP | CA.PGAv.1.6.scaffold497.74 | *Capsicum annuum* | eudicot |
| Type II (MIKC) | SEP | CA.PGAv.1.6.scaffold1592.4 | *Capsicum annuum* | eudicot |
| Type II (MIKC) | SEP | CA.PGAv.1.6.scaffold263.6 | *Capsicum annuum* | eudicot |
| Type II (MIKC) | SEP | CA.PGAv.1.6.scaffold1021.30 | *Capsicum annuum* | eudicot |
| Type II (MIKC) | SEP | CA.PGAv.1.6.scaffold1091.1 | *Capsicum annuum* | eudicot |
| Type II (MIKC) | SEP | CA.PGAv.1.6.scaffold13.5 | *Capsicum annuum* | eudicot |
| Type II (MIKC) | SEP | Cg7g016020.1 | *Citrus grandis* | eudicot |
| Type II (MIKC) | SEP | Cg6g020630.1 | *Citrus grandis* | eudicot |
| Type II (MIKC) | SEP | Cg5g011730.1 | *Citrus grandis* | eudicot |
| Type II (MIKC) | SEP | Cg7g016780.1 | *Citrus grandis* | eudicot |
| Type II (MIKC) | SEP | XP_008350888.2 | *Malus domestica* | eudicot |
| Type II (MIKC) | SEP | XP_017183980.1 | *Malus domestica* | eudicot |
| Type II (MIKC) | SEP | XP_008360083.1 | *Malus domestica* | eudicot |
| Type II (MIKC) | SEP | XP_008341807.1 | *Malus domestica* | eudicot |
| Type II (MIKC) | SEP | XP_008365428.1 | *Malus domestica* | eudicot |
| Type II (MIKC) | SEP | XP_008357795.1 | *Malus domestica* | eudicot |
| Type II (MIKC) | SEP | NP_001281016.1 | *Malus domestica* | eudicot |
| Type II (MIKC) | SEP | XP_017188112.1 | *Malus domestica* | eudicot |
| Type II (MIKC) | SEP | NP_001280756.1 | *Malus domestica* | eudicot |
| Type II (MIKC) | SEP | XP_018502992.1 | *Pyrus x bretschneideri* | eudicot |
| Type II (MIKC) | SEP | XP_009373783.1 | *Pyrus x bretschneideri* | eudicot |
| Type II (MIKC) | SEP | XP_009333662.1 | *Pyrus x bretschneideri* | eudicot |
| Type II (MIKC) | SEP | XP_009378252.1 | *Pyrus x bretschneideri* | eudicot |
| Type II (MIKC) | SEP | XP_009337956.1 | *Pyrus x bretschneideri* | eudicot |
| Type II (MIKC) | SEP | XP_018500628.1 | *Pyrus x bretschneideri* | eudicot |
| Type II (MIKC) | SEP | XP_018500632.1 | *Pyrus x bretschneideri* | eudicot |
| Type II (MIKC) | SEP | XP_009372256.1 | *Pyrus x bretschneideri* | eudicot |
| Type II (MIKC) | SEP | NP_001289232.1 | *Pyrus x bretschneideri* | eudicot |
| Type II (MIKC) | SEP | GSVIVT01036551001 | *Vitis vinifera* | eudicot |
| Type II (MIKC) | SEP | GSVIVT01012249001 | *Vitis vinifera* | eudicot |
| Type II (MIKC) | SEP | GSVIVT01008139001 | *Vitis vinifera* | eudicot |
| Type II (MIKC) | SEP | GSVIVT01010521001 | *Vitis vinifera* | eudicot |
| Type II (MIKC) | SEP | Gb_36364 | *Ginkgo biloba* | gymnosperm |
| Type II (MIKC) | SEP | Gb_41549 | *Ginkgo biloba* | gymnosperm |
| Type II (MIKC) | SEP | Aco017563.1 | *Ananas comosus* | monocot |
| Type II (MIKC) | SEP | Aco015105.1 | *Ananas comosus* | monocot |
| Type II (MIKC) | SEP | Bradi1g08326.1 | *Brachypodium distachyon* | monocot |
| Type II (MIKC) | SEP | Bradi1g48520.1 | *Brachypodium distachyon* | monocot |
| Type II (MIKC) | SEP | Bradi1g69890.1 | *Brachypodium distachyon* | monocot |
| Type II (MIKC) | SEP | Bradi3g41260.1 | *Brachypodium distachyon* | monocot |
| Type II (MIKC) | SEP | Bradi4g34680.1 | *Brachypodium distachyon* | monocot |
| Type II (MIKC) | SEP | Dof021261 ( DoAGL13 ) | *Dendrobium officinale* | monocot |
| Type II (MIKC) | SEP | Dof003023 ( DoAGL12 ) | *Dendrobium officinale* | monocot |
| Type II (MIKC) | SEP | Dof016731 ( DoAGL11 ) | *Dendrobium officinale* | monocot |
| Type II (MIKC) | SEP | Dof016730 ( DoAGL9 ) | *Dendrobium officinale* | monocot |
| Type II (MIKC) | SEP | Dof018065 ( DoAGL10 ) | *Dendrobium officinale* | monocot |
| Type II (MIKC) | SEP | Ma02_p08180.1 | *Musa acuminata* | monocot |
| Type II (MIKC) | SEP | Ma08_p28680.1 | *Musa acuminata* | monocot |
| Type II (MIKC) | SEP | Ma03_p17290.1 | *Musa acuminata* | monocot |
| Type II (MIKC) | SEP | Ma07_p28230.1 | *Musa acuminata* | monocot |
| Type II (MIKC) | SEP | Ma04_p14430.1 | *Musa acuminata* | monocot |
| Type II (MIKC) | SEP | Ma09_p01340.1 | *Musa acuminata* | monocot |
| Type II (MIKC) | SEP | Ma06_p07660.1 | *Musa acuminata* | monocot |
| Type II (MIKC) | SEP | Ma09_p29900.1 | *Musa acuminata* | monocot |
| Type II (MIKC) | SEP | Ma11_p17960.1 | *Musa acuminata* | monocot |
| Type II (MIKC) | SEP | LOC_Os03g54170.1 | *Oryza sativa* | monocot |
| Type II (MIKC) | SEP | LOC_Os06g06750.1 | *Oryza sativa* | monocot |
| Type II (MIKC) | SEP | LOC_Os03g11614.1 | *Oryza sativa* | monocot |
| Type II (MIKC) | SEP | LOC_Os08g41950.1 | *Oryza sativa* | monocot |
| Type II (MIKC) | SEP | LOC_Os09g32948.1 | *Oryza sativa* | monocot |
| Type II (MIKC) | SEP | LOC110019564 ( PeAGL7 ) | *Phalaenopsis equestris* | monocot |
| Type II (MIKC) | SEP | LOC110031960 ( PeAGL8 ) | *Phalaenopsis equestris* | monocot |
| Type II (MIKC) | SEP | LOC110018815 ( PeAGL9 ) | *Phalaenopsis equestris* | monocot |
| Type II (MIKC) | SEP | LOC110039099 ( PeAGL6 ) | *Phalaenopsis equestris* | monocot |
| Type II (MIKC) | SEP | LOC110033309 ( PeAGL5 ) | *Phalaenopsis equestris* | monocot |
| Type II (MIKC) | SEP | Sb01g007780.1 | *Sorghum bicolor* | monocot |
| Type II (MIKC) | SEP | Sb10g004390.1 | *Sorghum bicolor* | monocot |
| Type II (MIKC) | SEP | Sb01g042840.1 | *Sorghum bicolor* | monocot |
| Type II (MIKC) | SEP | Sb07g026200.1 | *Sorghum bicolor* | monocot |
| Type II (MIKC) | SEP | Sb02g029310.1 | *Sorghum bicolor* | monocot |
| Type II (MIKC) | SEP | TRIUR3_28280 | *Triticum aestivum* | monocot |
| Type II (MIKC) | SEP | TRIUR3_12423 | *Triticum aestivum* | monocot |
| Type II (MIKC) | SEP | TRIUR3_24374 | *Triticum aestivum* | monocot |
| Type II (MIKC) | SEP | TRIUR3_30491 | *Triticum aestivum* | monocot |
| Type II (MIKC) | SEP | TRIUR3_18949 | *Triticum aestivum* | monocot |
| Type II (MIKC) | SEP | TRIUR3_28216 | *Triticum aestivum* | monocot |
| Type II (MIKC) | SEP | GRMZM2G071620_P01 | *Zea mays* | monocot |
| Type II (MIKC) | SEP | GRMZM2G087095_P01 | *Zea mays* | monocot |
| Type II (MIKC) | SEP | GRMZM2G102161_P01 | *Zea mays* | monocot |
| Type II (MIKC) | SEP | GRMZM2G099522_P01 | *Zea mays* | monocot |
| Type II (MIKC) | SEP | AC195587.4_FGP001 | *Zea mays* | monocot |
| Type II (MIKC) | SEP | GRMZM2G159397_P01 | *Zea mays* | monocot |
| Type II (MIKC) | SEP | GRMZM2G097059_P01 | *Zea mays* | monocot |
| Type II (MIKC) | SEP | GRMZM2G129034_P01 | *Zea mays* | monocot |
| Type II (MIKC) | SQUA | evm_27.model.AmTr_v1.0_scaffold00047.105 | *Amborella trichopoda* | basal angiosperm |
| Type II (MIKC) | SQUA | NNU_07467-RA | *Nelumbo nucifera* | basal angiosperm |
| Type II (MIKC) | SQUA | Achn247791 | *Actinidia chinensis* | eudicot |
| Type II (MIKC) | SQUA | Achn245331 | *Actinidia chinensis* | eudicot |
| Type II (MIKC) | SQUA | Achn143731 | *Actinidia chinensis* | eudicot |
| Type II (MIKC) | SQUA | Achn356291 | *Actinidia chinensis* | eudicot |
| Type II (MIKC) | SQUA | Achn293721 | *Actinidia chinensis* | eudicot |
| Type II (MIKC) | SQUA | AtAGL8 | *Arabidopsis thaliana* | eudicot |
| Type II (MIKC) | SQUA | AtCAL | *Arabidopsis thaliana* | eudicot |
| Type II (MIKC) | SQUA | AtAP1 | *Arabidopsis thaliana* | eudicot |
| Type II (MIKC) | SQUA | AtAGL79 | *Arabidopsis thaliana* | eudicot |
| Type II (MIKC) | SQUA | Bra035952 | *Brassica rapa* | eudicot |
| Type II (MIKC) | SQUA | Bra012997 | *Brassica rapa* | eudicot |
| Type II (MIKC) | SQUA | Bra029347 | *Brassica rapa* | eudicot |
| Type II (MIKC) | SQUA | Bra011021 | *Brassica rapa* | eudicot |
| Type II (MIKC) | SQUA | Bra004361 | *Brassica rapa* | eudicot |
| Type II (MIKC) | SQUA | Bra004007 | *Brassica rapa* | eudicot |
| Type II (MIKC) | SQUA | Bra038326 | *Brassica rapa* | eudicot |
| Type II (MIKC) | SQUA | Bra036201 | *Brassica rapa* | eudicot |
| Type II (MIKC) | SQUA | Bra020742 | *Brassica rapa* | eudicot |
| Type II (MIKC) | SQUA | Bra025411 | *Brassica rapa* | eudicot |
| Type II (MIKC) | SQUA | CA.PGAv.1.6.scaffold1592.5 | *Capsicum annuum* | eudicot |
| Type II (MIKC) | SQUA | CA.PGAv.1.6.scaffold587.54 | *Capsicum annuum* | eudicot |
| Type II (MIKC) | SQUA | CA.PGAv.1.6.scaffold497.73 | *Capsicum annuum* | eudicot |
| Type II (MIKC) | SQUA | CA.PGAv.1.6.scaffold93.50 | *Capsicum annuum* | eudicot |
| Type II (MIKC) | SQUA | CA.PGAv.1.6.scaffold7478.1 | *Capsicum annuum* | eudicot |
| Type II (MIKC) | SQUA | Cg5g011740.1 | *Citrus grandis* | eudicot |
| Type II (MIKC) | SQUA | Cg6g020650.1 | *Citrus grandis* | eudicot |
| Type II (MIKC) | SQUA | Cg7g016010.1 | *Citrus grandis* | eudicot |
| Type II (MIKC) | SQUA | XP_008374664.1 | *Malus domestica* | eudicot |
| Type II (MIKC) | SQUA | XP_008393257.1 | *Malus domestica* | eudicot |
| Type II (MIKC) | SQUA | NP_001280769.1 | *Malus domestica* | eudicot |
| Type II (MIKC) | SQUA | NP_001280866.1 | *Malus domestica* | eudicot |
| Type II (MIKC) | SQUA | XP_018500640.1 | *Pyrus x bretschneideri* | eudicot |
| Type II (MIKC) | SQUA | XP_018498762.1 | *Pyrus x bretschneideri* | eudicot |
| Type II (MIKC) | SQUA | XP_009378257.1 | *Pyrus x bretschneideri* | eudicot |
| Type II (MIKC) | SQUA | XP_009373778.1 | *Pyrus x bretschneideri* | eudicot |
| Type II (MIKC) | SQUA | XP_018497885.1 | *Pyrus x bretschneideri* | eudicot |
| Type II (MIKC) | SQUA | XP_009356440.1 | *Pyrus x bretschneideri* | eudicot |
| Type II (MIKC) | SQUA | GSVIVT01008140001 | *Vitis vinifera* | eudicot |
| Type II (MIKC) | SQUA | GSVIVT01036549001 | *Vitis vinifera* | eudicot |
| Type II (MIKC) | SQUA | GSVIVT01012250001 | *Vitis vinifera* | eudicot |
| Type II (MIKC) | SQUA | Aco012428.1 | *Ananas comosus* | monocot |
| Type II (MIKC) | SQUA | Aco004839.1 | *Ananas comosus* | monocot |
| Type II (MIKC) | SQUA | Aco019039.1 | *Ananas comosus* | monocot |
| Type II (MIKC) | SQUA | Aco015104.1 | *Ananas comosus* | monocot |
| Type II (MIKC) | SQUA | Bradi1g08340.1 | *Brachypodium distachyon* | monocot |
| Type II (MIKC) | SQUA | Bradi1g59250.1 | *Brachypodium distachyon* | monocot |
| Type II (MIKC) | SQUA | Bradi1g21980.2 | *Brachypodium distachyon* | monocot |
| Type II (MIKC) | SQUA | Bradi4g06867.1 | *Brachypodium distachyon* | monocot |
| Type II (MIKC) | SQUA | Bradi5g08027.1 | *Brachypodium distachyon* | monocot |
| Type II (MIKC) | SQUA | Bradi3g41297.1 | *Brachypodium distachyon* | monocot |
| Type II (MIKC) | SQUA | Bradi4g30090.1 | *Brachypodium distachyon* | monocot |
| Type II (MIKC) | SQUA | Bradi2g59187.1 | *Brachypodium distachyon* | monocot |
| Type II (MIKC) | SQUA | Bradi2g59120.2 | *Brachypodium distachyon* | monocot |
| Type II (MIKC) | SQUA | Dof003026 ( DoAGL18 ) | *Dendrobium officinale* | monocot |
| Type II (MIKC) | SQUA | Dof022092 ( DoAGL19 ) | *Dendrobium officinale* | monocot |
| Type II (MIKC) | SQUA | Dof002059 ( DoAGL17 ) | *Dendrobium officinale* | monocot |
| Type II (MIKC) | SQUA | Dof017703 ( DoAGL16 ) | *Dendrobium officinale* | monocot |
| Type II (MIKC) | SQUA | Ma03_p26480.1 | *Musa acuminata* | monocot |
| Type II (MIKC) | SQUA | Ma08_p04270.1 | *Musa acuminata* | monocot |
| Type II (MIKC) | SQUA | Ma03_p31640.1 | *Musa acuminata* | monocot |
| Type II (MIKC) | SQUA | Ma04_p30020.1 | *Musa acuminata* | monocot |
| Type II (MIKC) | SQUA | Ma07_p00440.1 | *Musa acuminata* | monocot |
| Type II (MIKC) | SQUA | Ma02_p15490.1 | *Musa acuminata* | monocot |
| Type II (MIKC) | SQUA | Ma06_p01760.1 | *Musa acuminata* | monocot |
| Type II (MIKC) | SQUA | Ma03_p02150.1 | *Musa acuminata* | monocot |
| Type II (MIKC) | SQUA | Ma10_p18550.1 | *Musa acuminata* | monocot |
| Type II (MIKC) | SQUA | Ma08_p28790.1 | *Musa acuminata* | monocot |
| Type II (MIKC) | SQUA | Ma08_p08940.1 | *Musa acuminata* | monocot |
| Type II (MIKC) | SQUA | Ma11_p14180.1 | *Musa acuminata* | monocot |
| Type II (MIKC) | SQUA | Ma11_p14170.1 | *Musa acuminata* | monocot |
| Type II (MIKC) | SQUA | Ma08_p08930.1 | *Musa acuminata* | monocot |
| Type II (MIKC) | SQUA | Ma02_p02200.1 | *Musa acuminata* | monocot |
| Type II (MIKC) | SQUA | LOC_Os03g54160.1 | *Oryza sativa* | monocot |
| Type II (MIKC) | SQUA | LOC_Os07g01820.1 | *Oryza sativa* | monocot |
| Type II (MIKC) | SQUA | LOC_Os07g41370.1 | *Oryza sativa* | monocot |
| Type II (MIKC) | SQUA | LOC_Os12g31748.1 | *Oryza sativa* | monocot |
| Type II (MIKC) | SQUA | LOC_Os04g31804.1 | *Oryza sativa* | monocot |
| Type II (MIKC) | SQUA | LOC_Os08g41960.1 | *Oryza sativa* | monocot |
| Type II (MIKC) | SQUA | LOC_Os01g69850.1 | *Oryza sativa* | monocot |
| Type II (MIKC) | SQUA | LOC110031959 ( PeAGL13 ) | *Phalaenopsis equestris* | monocot |
| Type II (MIKC) | SQUA | LOC110019558 ( PeAGL11 ) | *Phalaenopsis equestris* | monocot |
| Type II (MIKC) | SQUA | LOC110033613 ( PeAGL12 ) | *Phalaenopsis equestris* | monocot |
| Type II (MIKC) | SQUA | Sb01g007790.1 | *Sorghum bicolor* | monocot |
| Type II (MIKC) | SQUA | Sb02g001090.1 | *Sorghum bicolor* | monocot |
| Type II (MIKC) | SQUA | Sb02g038780.1 | *Sorghum bicolor* | monocot |
| Type II (MIKC) | SQUA | Sb08g015555.1 | *Sorghum bicolor* | monocot |
| Type II (MIKC) | SQUA | Sb07g026180.1 | *Sorghum bicolor* | monocot |
| Type II (MIKC) | SQUA | Sb03g006210.1 | *Sorghum bicolor* | monocot |
| Type II (MIKC) | SQUA | Sb03g044170.1 | *Sorghum bicolor* | monocot |
| Type II (MIKC) | SQUA | TRIUR3_21876 | *Triticum aestivum* | monocot |
| Type II (MIKC) | SQUA | TRIUR3_20925 | *Triticum aestivum* | monocot |
| Type II (MIKC) | SQUA | TRIUR3_21732 | *Triticum aestivum* | monocot |
| Type II (MIKC) | SQUA | TRIUR3_30073 | *Triticum aestivum* | monocot |
| Type II (MIKC) | SQUA | TRIUR3_20985 | *Triticum aestivum* | monocot |
| Type II (MIKC) | SQUA | TRIUR3_27077 | *Triticum aestivum* | monocot |
| Type II (MIKC) | SQUA | TRIUR3_30872 | *Triticum aestivum* | monocot |
| Type II (MIKC) | SQUA | TRIUR3_19056 | *Triticum aestivum* | monocot |
| Type II (MIKC) | SQUA | TRIUR3_28473 | *Triticum aestivum* | monocot |
| Type II (MIKC) | SQUA | GRMZM2G553379_P01 | *Zea mays* | monocot |
| Type II (MIKC) | SQUA | GRMZM2G032339_P02 | *Zea mays* | monocot |
| Type II (MIKC) | SQUA | GRMZM2G072582_P02 | *Zea mays* | monocot |
| Type II (MIKC) | SQUA | GRMZM2G148693_P01 | *Zea mays* | monocot |
| Type II (MIKC) | SQUA | GRMZM2G147716_P01 | *Zea mays* | monocot |
| Type II (MIKC) | SQUA | GRMZM2G137510_P01 | *Zea mays* | monocot |
| Type II (MIKC) | SQUA | GRMZM2G098986_P01 | *Zea mays* | monocot |
| Type II (MIKC) | SQUA | GRMZM2G357984_P01 | *Zea mays* | monocot |
| Type II (MIKC) | SQUA | GRMZM2G320549_P01 | *Zea mays* | monocot |
| Type II (MIKC) | SQUA | GRMZM2G171650_P01 | *Zea mays* | monocot |
| Type II (MIKC) | SVP | evm_27.model.AmTr_v1.0_scaffold00127.17 | *Amborella trichopoda* | basal angiosperm |
| Type II (MIKC) | SVP | NNU_12844-RA | *Nelumbo nucifera* | basal angiosperm |
| Type II (MIKC) | SVP | NNU_05169-RA | *Nelumbo nucifera* | basal angiosperm |
| Type II (MIKC) | SVP | Mapoly0011s0161.1.p | *Marchantia polymorpha* | bryophyte |
| Type II (MIKC) | SVP | EDQ58545.1 | *Physcomitrella patens* | bryophyte |
| Type II (MIKC) | SVP | EDQ77973.1 | *Physcomitrella patens* | bryophyte |
| Type II (MIKC) | SVP | EDQ61137.1 | *Physcomitrella patens* | bryophyte |
| Type II (MIKC) | SVP | EDQ55286.1 | *Physcomitrella patens* | bryophyte |
| Type II (MIKC) | SVP | EDQ65424.1 | *Physcomitrella patens* | bryophyte |
| Type II (MIKC) | SVP | EDQ72735.1 | *Physcomitrella patens* | bryophyte |
| Type II (MIKC) | SVP | Sphfalx0119s0043.1.p | *Sphagnum fallax* | bryophyte |
| Type II (MIKC) | SVP | Sphfalx0014s0200.1.p | *Sphagnum fallax* | bryophyte |
| Type II (MIKC) | SVP | Sphfalx0101s0028.1.p | *Sphagnum fallax* | bryophyte |
| Type II (MIKC) | SVP | Sphfalx0209s0007.1.p | *Sphagnum fallax* | bryophyte |
| Type II (MIKC) | SVP | GAQ89767.1 | *Klebsormidium nitens* | charophyte |
| Type II (MIKC) | SVP | Achn041671 | *Actinidia chinensis* | eudicot |
| Type II (MIKC) | SVP | Achn087751 | *Actinidia chinensis* | eudicot |
| Type II (MIKC) | SVP | Achn340121 | *Actinidia chinensis* | eudicot |
| Type II (MIKC) | SVP | Achn215131 | *Actinidia chinensis* | eudicot |
| Type II (MIKC) | SVP | Achn340131 | *Actinidia chinensis* | eudicot |
| Type II (MIKC) | SVP | Achn224361 | *Actinidia chinensis* | eudicot |
| Type II (MIKC) | SVP | Achn332401 | *Actinidia chinensis* | eudicot |
| Type II (MIKC) | SVP | Achn171711 | *Actinidia chinensis* | eudicot |
| Type II (MIKC) | SVP | AtAGL24 | *Arabidopsis thaliana* | eudicot |
| Type II (MIKC) | SVP | AtSVP | *Arabidopsis thaliana* | eudicot |
| Type II (MIKC) | SVP | Bra013812 | *Brassica rapa* | eudicot |
| Type II (MIKC) | SVP | Bra019221 | *Brassica rapa* | eudicot |
| Type II (MIKC) | SVP | Bra038511 | *Brassica rapa* | eudicot |
| Type II (MIKC) | SVP | Bra030228 | *Brassica rapa* | eudicot |
| Type II (MIKC) | SVP | CA.PGAv.1.6.scaffold716.7 | *Capsicum annuum* | eudicot |
| Type II (MIKC) | SVP | CA.PGAv.1.6.scaffold523.13 | *Capsicum annuum* | eudicot |
| Type II (MIKC) | SVP | CA.PGAv.1.6.scaffold1344.3 | *Capsicum annuum* | eudicot |
| Type II (MIKC) | SVP | CA.PGAv.1.6.scaffold475.33 | *Capsicum annuum* | eudicot |
| Type II (MIKC) | SVP | Cg1g019310.1 | *Citrus grandis* | eudicot |
| Type II (MIKC) | SVP | Cg1g007910.1 | *Citrus grandis* | eudicot |
| Type II (MIKC) | SVP | XP_017186028.1 | *Malus domestica* | eudicot |
| Type II (MIKC) | SVP | XP_008378582.1 | *Malus domestica* | eudicot |
| Type II (MIKC) | SVP | XP_017179220.1 | *Malus domestica* | eudicot |
| Type II (MIKC) | SVP | XP_008353306.1 | *Malus domestica* | eudicot |
| Type II (MIKC) | SVP | XP_008341075.1 | *Malus domestica* | eudicot |
| Type II (MIKC) | SVP | XP_017181158.1 | *Malus domestica* | eudicot |
| Type II (MIKC) | SVP | XP_017189345.1 | *Malus domestica* | eudicot |
| Type II (MIKC) | SVP | XP_008379458.1 | *Malus domestica* | eudicot |
| Type II (MIKC) | SVP | XP_017185625.1 | *Malus domestica* | eudicot |
| Type II (MIKC) | SVP | XP_008385673.1 | *Malus domestica* | eudicot |
| Type II (MIKC) | SVP | XP_018507346.1 | *Pyrus x bretschneideri* | eudicot |
| Type II (MIKC) | SVP | XP_018507345.1 | *Pyrus x bretschneideri* | eudicot |
| Type II (MIKC) | SVP | XP_009376227.1 | *Pyrus x bretschneideri* | eudicot |
| Type II (MIKC) | SVP | XP_018500348.1 | *Pyrus x bretschneideri* | eudicot |
| Type II (MIKC) | SVP | XP_018507347.1 | *Pyrus x bretschneideri* | eudicot |
| Type II (MIKC) | SVP | XP_009345716.1 | *Pyrus x bretschneideri* | eudicot |
| Type II (MIKC) | SVP | XP_009364260.1 | *Pyrus x bretschneideri* | eudicot |
| Type II (MIKC) | SVP | GSVIVT01019630001 | *Vitis vinifera* | eudicot |
| Type II (MIKC) | SVP | GSVIVT01001701001 | *Vitis vinifera* | eudicot |
| Type II (MIKC) | SVP | GSVIVT01015641001 | *Vitis vinifera* | eudicot |
| Type II (MIKC) | SVP | GSVIVT01011300001 | *Vitis vinifera* | eudicot |
| Type II (MIKC) | SVP | GSVIVT01002442001 | *Vitis vinifera* | eudicot |
| Type II (MIKC) | SVP | GSVIVT01019167001 | *Vitis vinifera* | eudicot |
| Type II (MIKC) | SVP | GSVIVT01019180001 | *Vitis vinifera* | eudicot |
| Type II (MIKC) | SVP | GSVIVT01009171001 | *Vitis vinifera* | eudicot |
| Type II (MIKC) | SVP | GSVIVT01005934001 | *Vitis vinifera* | eudicot |
| Type II (MIKC) | SVP | Gb_05128 | *Ginkgo biloba* | gymnosperm |
| Type II (MIKC) | SVP | Gb_34103 | *Ginkgo biloba* | gymnosperm |
| Type II (MIKC) | SVP | EFJ21171.1 | *Selaginella moellendorffii* | lycophyte |
| Type II (MIKC) | SVP | EFJ19381.1 | *Selaginella moellendorffii* | lycophyte |
| Type II (MIKC) | SVP | EFJ13151.1 | *Selaginella moellendorffii* | lycophyte |
| Type II (MIKC) | SVP | EFJ14171.1 | *Selaginella moellendorffii* | lycophyte |
| Type II (MIKC) | SVP | Aco004028.1 | *Ananas comosus* | monocot |
| Type II (MIKC) | SVP | Aco027879.1 | *Ananas comosus* | monocot |
| Type II (MIKC) | SVP | Aco002729.1 | *Ananas comosus* | monocot |
| Type II (MIKC) | SVP | Bradi1g45807.1 | *Brachypodium distachyon* | monocot |
| Type II (MIKC) | SVP | Bradi3g58220.1 | *Brachypodium distachyon* | monocot |
| Type II (MIKC) | SVP | Bradi1g72150.1 | *Brachypodium distachyon* | monocot |
| Type II (MIKC) | SVP | Dof012304 ( DoAGL27 ) | *Dendrobium officinale* | monocot |
| Type II (MIKC) | SVP | Dof013716 ( DoAGL28 ) | *Dendrobium officinale* | monocot |
| Type II (MIKC) | SVP | Dof024877 ( DoAGL29 ) | *Dendrobium officinale* | monocot |
| Type II (MIKC) | SVP | Ma10_p19310.1 | *Musa acuminata* | monocot |
| Type II (MIKC) | SVP | Ma03_p06740.1 | *Musa acuminata* | monocot |
| Type II (MIKC) | SVP | Ma09_p14590.1 | *Musa acuminata* | monocot |
| Type II (MIKC) | SVP | LOC_Os06g11330.1 | *Oryza sativa* | monocot |
| Type II (MIKC) | SVP | LOC_Os02g52340.1 | *Oryza sativa* | monocot |
| Type II (MIKC) | SVP | LOC_Os03g08754.1 | *Oryza sativa* | monocot |
| Type II (MIKC) | SVP | LOC110021474 ( PeAGL24 ) | *Phalaenopsis equestris* | monocot |
| Type II (MIKC) | SVP | Sb10g007380.1 | *Sorghum bicolor* | monocot |
| Type II (MIKC) | SVP | Sb04g033930.1 | *Sorghum bicolor* | monocot |
| Type II (MIKC) | SVP | Sb01g044810.1 | *Sorghum bicolor* | monocot |
| Type II (MIKC) | SVP | TRIUR3_03219 | *Triticum aestivum* | monocot |
| Type II (MIKC) | SVP | GRMZM5G814279_P01 | *Zea mays* | monocot |
| Type II (MIKC) | SVP | GRMZM2G370777_P01 | *Zea mays* | monocot |
| Type II (MIKC) | SVP | GRMZM2G046885_P01 | *Zea mays* | monocot |
| Type II (MIKC) | SVP | GRMZM2G059102_P01 | *Zea mays* | monocot |
| Type II (MIKC) | TM3/SOC1 | evm_27.model.AmTr_v1.0_scaffold00001.409 | *Amborella trichopoda* | basal angiosperm |
| Type II (MIKC) | TM3/SOC1 | NNU_26572-RA | *Nelumbo nucifera* | basal angiosperm |
| Type II (MIKC) | TM3/SOC1 | NNU_08843-RA | *Nelumbo nucifera* | basal angiosperm |
| Type II (MIKC) | TM3/SOC1 | Achn354091 | *Actinidia chinensis* | eudicot |
| Type II (MIKC) | TM3/SOC1 | Achn002551 | *Actinidia chinensis* | eudicot |
| Type II (MIKC) | TM3/SOC1 | Achn327221 | *Actinidia chinensis* | eudicot |
| Type II (MIKC) | TM3/SOC1 | Achn196121 | *Actinidia chinensis* | eudicot |
| Type II (MIKC) | TM3/SOC1 | AtAGL42 | *Arabidopsis thaliana* | eudicot |
| Type II (MIKC) | TM3/SOC1 | AtAGL72 | *Arabidopsis thaliana* | eudicot |
| Type II (MIKC) | TM3/SOC1 | AtAGL71 | *Arabidopsis thaliana* | eudicot |
| Type II (MIKC) | TM3/SOC1 | AtAGL19 | *Arabidopsis thaliana* | eudicot |
| Type II (MIKC) | TM3/SOC1 | AtAGL14 | *Arabidopsis thaliana* | eudicot |
| Type II (MIKC) | TM3/SOC1 | AtAGL20 | *Arabidopsis thaliana* | eudicot |
| Type II (MIKC) | TM3/SOC1 | Bra035907 | *Brassica rapa* | eudicot |
| Type II (MIKC) | TM3/SOC1 | Bra029281 | *Brassica rapa* | eudicot |
| Type II (MIKC) | TM3/SOC1 | Bra010465 | *Brassica rapa* | eudicot |
| Type II (MIKC) | TM3/SOC1 | Bra013891 | *Brassica rapa* | eudicot |
| Type II (MIKC) | TM3/SOC1 | Bra012957 | *Brassica rapa* | eudicot |
| Type II (MIKC) | TM3/SOC1 | Bra029314 | *Brassica rapa* | eudicot |
| Type II (MIKC) | TM3/SOC1 | Bra028282 | *Brassica rapa* | eudicot |
| Type II (MIKC) | TM3/SOC1 | Bra029155 | *Brassica rapa* | eudicot |
| Type II (MIKC) | TM3/SOC1 | Bra028283 | *Brassica rapa* | eudicot |
| Type II (MIKC) | TM3/SOC1 | Bra029154 | *Brassica rapa* | eudicot |
| Type II (MIKC) | TM3/SOC1 | Bra019343 | *Brassica rapa* | eudicot |
| Type II (MIKC) | TM3/SOC1 | Bra020826 | *Brassica rapa* | eudicot |
| Type II (MIKC) | TM3/SOC1 | Bra029424 | *Brassica rapa* | eudicot |
| Type II (MIKC) | TM3/SOC1 | Bra004928 | *Brassica rapa* | eudicot |
| Type II (MIKC) | TM3/SOC1 | Bra000393 | *Brassica rapa* | eudicot |
| Type II (MIKC) | TM3/SOC1 | Bra039324 | *Brassica rapa* | eudicot |
| Type II (MIKC) | TM3/SOC1 | CA.PGAv.1.6.scaffold1182.1 | *Capsicum annuum* | eudicot |
| Type II (MIKC) | TM3/SOC1 | CA.PGAv.1.6.scaffold981.57 | *Capsicum annuum* | eudicot |
| Type II (MIKC) | TM3/SOC1 | CA.PGAv.1.6.scaffold26.37 | *Capsicum annuum* | eudicot |
| Type II (MIKC) | TM3/SOC1 | CA.PGAv.1.6.scaffold861.2 | *Capsicum annuum* | eudicot |
| Type II (MIKC) | TM3/SOC1 | CA.PGAv.1.6.scaffold91.15 | *Capsicum annuum* | eudicot |
| Type II (MIKC) | TM3/SOC1 | CA.PGAv.1.6.scaffold170.68 | *Capsicum annuum* | eudicot |
| Type II (MIKC) | TM3/SOC1 | CA.PGAv.1.6.scaffold1561.1 | *Capsicum annuum* | eudicot |
| Type II (MIKC) | TM3/SOC1 | Cg9g026060.1 | *Citrus grandis* | eudicot |
| Type II (MIKC) | TM3/SOC1 | Cg5g009540.1 | *Citrus grandis* | eudicot |
| Type II (MIKC) | TM3/SOC1 | Cg5g042940.1 | *Citrus grandis* | eudicot |
| Type II (MIKC) | TM3/SOC1 | XP_017190419.1 | *Malus domestica* | eudicot |
| Type II (MIKC) | TM3/SOC1 | XP_008376871.1 | *Malus domestica* | eudicot |
| Type II (MIKC) | TM3/SOC1 | XP_008343719.1 | *Malus domestica* | eudicot |
| Type II (MIKC) | TM3/SOC1 | XP_017189909.1 | *Malus domestica* | eudicot |
| Type II (MIKC) | TM3/SOC1 | NP_001280778.1 | *Malus domestica* | eudicot |
| Type II (MIKC) | TM3/SOC1 | XP_008356915.1 | *Malus domestica* | eudicot |
| Type II (MIKC) | TM3/SOC1 | XP_008342912.1 | *Malus domestica* | eudicot |
| Type II (MIKC) | TM3/SOC1 | XP_018499527.1 | *Pyrus x bretschneideri* | eudicot |
| Type II (MIKC) | TM3/SOC1 | XP_009347903.1 | *Pyrus x bretschneideri* | eudicot |
| Type II (MIKC) | TM3/SOC1 | XP_009371259.1 | *Pyrus x bretschneideri* | eudicot |
| Type II (MIKC) | TM3/SOC1 | XP_018501073.1 | *Pyrus x bretschneideri* | eudicot |
| Type II (MIKC) | TM3/SOC1 | XP_009347896.1 | *Pyrus x bretschneideri* | eudicot |
| Type II (MIKC) | TM3/SOC1 | XP_018499516.1 | *Pyrus x bretschneideri* | eudicot |
| Type II (MIKC) | TM3/SOC1 | XP_009362848.1 | *Pyrus x bretschneideri* | eudicot |
| Type II (MIKC) | TM3/SOC1 | XP_009345824.1 | *Pyrus x bretschneideri* | eudicot |
| Type II (MIKC) | TM3/SOC1 | XP_009345827.1 | *Pyrus x bretschneideri* | eudicot |
| Type II (MIKC) | TM3/SOC1 | XP_009375846.1 | *Pyrus x bretschneideri* | eudicot |
| Type II (MIKC) | TM3/SOC1 | GSVIVT01019883001 | *Vitis vinifera* | eudicot |
| Type II (MIKC) | TM3/SOC1 | GSVIVT01018446001 | *Vitis vinifera* | eudicot |
| Type II (MIKC) | TM3/SOC1 | GSVIVT01027579001 | *Vitis vinifera* | eudicot |
| Type II (MIKC) | TM3/SOC1 | Gb_12778 | *Ginkgo biloba* | gymnosperm |
| Type II (MIKC) | TM3/SOC1 | Aco015492.1 | *Ananas comosus* | monocot |
| Type II (MIKC) | TM3/SOC1 | Aco013229.1 | *Ananas comosus* | monocot |
| Type II (MIKC) | TM3/SOC1 | Aco017499.1 | *Ananas comosus* | monocot |
| Type II (MIKC) | TM3/SOC1 | Aco030142.1 | *Ananas comosus* | monocot |
| Type II (MIKC) | TM3/SOC1 | Aco016643.1 | *Ananas comosus* | monocot |
| Type II (MIKC) | TM3/SOC1 | Bradi3g00730.1 | *Brachypodium distachyon* | monocot |
| Type II (MIKC) | TM3/SOC1 | Bradi3g32090.1 | *Brachypodium distachyon* | monocot |
| Type II (MIKC) | TM3/SOC1 | Bradi1g77020.1 | *Brachypodium distachyon* | monocot |
| Type II (MIKC) | TM3/SOC1 | Dof014620 ( DoAGL24 ) | *Dendrobium officinale* | monocot |
| Type II (MIKC) | TM3/SOC1 | Dof019419 ( DoAGL25 ) | *Dendrobium officinale* | monocot |
| Type II (MIKC) | TM3/SOC1 | Ma10_p07670.1 | *Musa acuminata* | monocot |
| Type II (MIKC) | TM3/SOC1 | Ma11_p07440.1 | *Musa acuminata* | monocot |
| Type II (MIKC) | TM3/SOC1 | Ma02_p06310.1 | *Musa acuminata* | monocot |
| Type II (MIKC) | TM3/SOC1 | Ma01_p18380.1 | *Musa acuminata* | monocot |
| Type II (MIKC) | TM3/SOC1 | Ma04_p23400.1 | *Musa acuminata* | monocot |
| Type II (MIKC) | TM3/SOC1 | Ma05_p10000.1 | *Musa acuminata* | monocot |
| Type II (MIKC) | TM3/SOC1 | Ma02_p13660.1 | *Musa acuminata* | monocot |
| Type II (MIKC) | TM3/SOC1 | Ma08_p12030.1 | *Musa acuminata* | monocot |
| Type II (MIKC) | TM3/SOC1 | Ma11_p02670.1 | *Musa acuminata* | monocot |
| Type II (MIKC) | TM3/SOC1 | Ma11_p19840.1 | *Musa acuminata* | monocot |
| Type II (MIKC) | TM3/SOC1 | Ma11_p02680.1 | *Musa acuminata* | monocot |
| Type II (MIKC) | TM3/SOC1 | Ma11_p19830.1 | *Musa acuminata* | monocot |
| Type II (MIKC) | TM3/SOC1 | Ma05_p22690.1 | *Musa acuminata* | monocot |
| Type II (MIKC) | TM3/SOC1 | LOC_Os03g03100.1 | *Oryza sativa* | monocot |
| Type II (MIKC) | TM3/SOC1 | LOC_Os06g01890.1 | *Oryza sativa* | monocot |
| Type II (MIKC) | TM3/SOC1 | LOC_Os02g01365.1 | *Oryza sativa* | monocot |
| Type II (MIKC) | TM3/SOC1 | LOC_Os10g39130.1 | *Oryza sativa* | monocot |
| Type II (MIKC) | TM3/SOC1 | LOC110035055 ( PeAGL22 ) | *Phalaenopsis equestris* | monocot |
| Type II (MIKC) | TM3/SOC1 | LOC110020085 ( PeAGL23 ) | *Phalaenopsis equestris* | monocot |
| Type II (MIKC) | TM3/SOC1 | LOC110033833 ( PeAGL21 ) | *Phalaenopsis equestris* | monocot |
| Type II (MIKC) | TM3/SOC1 | Sb04g000500.1 | *Sorghum bicolor* | monocot |
| Type II (MIKC) | TM3/SOC1 | Sb01g030570.1 | *Sorghum bicolor* | monocot |
| Type II (MIKC) | TM3/SOC1 | Sb01g049020.1 | *Sorghum bicolor* | monocot |
| Type II (MIKC) | TM3/SOC1 | TRIUR3_22964 | *Triticum aestivum* | monocot |
| Type II (MIKC) | TM3/SOC1 | TRIUR3_18962 | *Triticum aestivum* | monocot |
| Type II (MIKC) | TM3/SOC1 | TRIUR3_32699 | *Triticum aestivum* | monocot |
| Type II (MIKC) | TM3/SOC1 | GRMZM2G070034_P01 | *Zea mays* | monocot |
| Type II (MIKC) | TM3/SOC1 | GRMZM2G026223_P01 | *Zea mays* | monocot |
| Type II (MIKC) | TM3/SOC1 | GRMZM2G171365_P01 | *Zea mays* | monocot |
| Type II (MIKC) | TM8 | evm_27.model.AmTr_v1.0_scaffold00013.60 | *Amborella trichopoda* | basal angiosperm |
| Type II (MIKC) | TM8 | NNU_22200-RA | *Nelumbo nucifera* | basal angiosperm |
| Type II (MIKC) | TM8 | Cg7g010270.1 | *Citrus grandis* | eudicot |
| Type II (MIKC) | TM8 | XP_017180836.1 | *Malus domestica* | eudicot |
| Type II (MIKC) | TM8 | XP_017192961.1 | *Malus domestica* | eudicot |
| Type II (MIKC) | TM8 | XP_018507109.1 | *Pyrus x bretschneideri* | eudicot |
| Type II (MIKC) | TM8 | XP_018500262.1 | *Pyrus x bretschneideri* | eudicot |
| Type II (MIKC) | TM8 | XP_009359418.1 | *Pyrus x bretschneideri* | eudicot |
| Type II (MIKC) | TM8 | GSVIVT01008560001 | *Vitis vinifera* | eudicot |
| Type II (MIKC) | TM8 | Gb_30604 | *Ginkgo biloba* | gymnosperm |
| Type II (MIKC) | TM8 | Gb_19178 | *Ginkgo biloba* | gymnosperm |
| Type II (MIKC) | TM8 | Gb_39109 | *Ginkgo biloba* | gymnosperm |
| Type II (MIKC) | TM8 | Gb_01884 | *Ginkgo biloba* | gymnosperm |
| Type II (MIKC) | TM8 | Gb_12581 | *Ginkgo biloba* | gymnosperm |
| Type II (MIKC) | TM8 | Gb_38365 | *Ginkgo biloba* | gymnosperm |
| Type II (MIKC) | TM8 | Gb_38922 | *Ginkgo biloba* | gymnosperm |
| Type II (MIKC) | TM8 | GRMZM2G137289_P01 | *Zea mays* | monocot |
| Type II (MIKC) | NG | Achn318871 | *Actinidia chinensis* | eudicot |
| Type II (MIKC) | NG | Achn379801 | *Actinidia chinensis* | eudicot |
| Type II (MIKC) | NG | Achn261611 | *Actinidia chinensis* | eudicot |
| Type II (MIKC) | NG | evm_27.model.AmTr_v1.0_scaffold00013.57 | *Amborella trichopoda* | basal angiosperm |
| Type II (MIKC) | NG | Cg2g033010.1 | *Citrus grandis* | eudicot |
| Type II (MIKC) | NG | Cg9g026030.1 | *Citrus grandis* | eudicot |
| Type II (MIKC) | NG | Dof011228 ( DoAGL26 ) | *Dendrobium officinale* | monocot |
| Type II (MIKC) | NG | Dof000889 ( DoAGL14 ) | *Dendrobium officinale* | monocot |
| Type II (MIKC) | NG | Gb_28587 | *Ginkgo biloba* | gymnosperm |
| Type II (MIKC) | NG | Gb_41550 | *Ginkgo biloba* | gymnosperm |
| Type II (MIKC) | NG | NNU_08847-RA | *Nelumbo nucifera* | basal angiosperm |
| Type II (MIKC) | NG | NNU_04431-RA | *Nelumbo nucifera* | basal angiosperm |
| Type II (MIKC) | NG | LOC110023273 ( PeAGL33 ) | *Phalaenopsis equestris* | monocot |
| Type II (MIKC) | NG | LOC110029972 ( PeAGL34 ) | *Phalaenopsis equestris* | monocot |
| Type II (MIKC) | NG | LOC110023272 ( PeAGL32 ) | *Phalaenopsis equestris* | monocot |
| Type II (MIKC) | NG | LOC110031217 ( PeAGL31 ) | *Phalaenopsis equestris* | monocot |
| Type II (MIKC) | NG | TRIUR3_19039 | *Triticum aestivum* | monocot |
| Type II (MIKC) | NG | TRIUR3_19009 | *Triticum aestivum* | monocot |
| Type II (MIKC) | MIKC*-P | evm_27.model.AmTr_v1.0_scaffold00010.504 | *Amborella trichopoda* | basal angiosperm |
| Type II (MIKC) | MIKC*-P | NNU_04643-RA | *Nelumbo nucifera* | basal angiosperm |
| Type II (MIKC) | MIKC*-P | NNU_07290-RA | *Nelumbo nucifera* | basal angiosperm |
| Type II (MIKC) | MIKC*-P | Sphfalx0096s0050.1.p | *Sphagnum fallax* | bryophyte |
| Type II (MIKC) | MIKC*-P | Sphfalx0096s0055.1.p | *Sphagnum fallax* | bryophyte |
| Type II (MIKC) | MIKC*-P | Sphfalx0247s0019.1.p | *Sphagnum fallax* | bryophyte |
| Type II (MIKC) | MIKC*-P | Achn077511 | *Actinidia chinensis* | eudicot |
| Type II (MIKC) | MIKC*-P | Achn208161 | *Actinidia chinensis* | eudicot |
| Type II (MIKC) | MIKC*-P | Achn243051 | *Actinidia chinensis* | eudicot |
| Type II (MIKC) | MIKC*-P | Achn389161 | *Actinidia chinensis* | eudicot |
| Type II (MIKC) | MIKC*-P | AtAGL30 | *Arabidopsis thaliana* | eudicot |
| Type II (MIKC) | MIKC*-P | AtAGL33 | *Arabidopsis thaliana* | eudicot |
| Type II (MIKC) | MIKC*-P | AtAGL65 | *Arabidopsis thaliana* | eudicot |
| Type II (MIKC) | MIKC*-P | AtAGL94 | *Arabidopsis thaliana* | eudicot |
| Type II (MIKC) | MIKC*-P | Bra004393 | *Brassica rapa* | eudicot |
| Type II (MIKC) | MIKC*-P | Bra016544 | *Brassica rapa* | eudicot |
| Type II (MIKC) | MIKC*-P | Bra017404 | *Brassica rapa* | eudicot |
| Type II (MIKC) | MIKC*-P | Bra024792 | *Brassica rapa* | eudicot |
| Type II (MIKC) | MIKC*-P | Bra025685 | *Brassica rapa* | eudicot |
| Type II (MIKC) | MIKC*-P | Bra031049 | *Brassica rapa* | eudicot |
| Type II (MIKC) | MIKC*-P | CA.PGAv.1.6.scaffold13.17 | *Capsicum annuum* | eudicot |
| Type II (MIKC) | MIKC*-P | CA.PGAv.1.6.scaffold86.16 | *Capsicum annuum* | eudicot |
| Type II (MIKC) | MIKC*-P | CA.PGAv.1.6.scaffold86.18 | *Capsicum annuum* | eudicot |
| Type II (MIKC) | MIKC*-P | CA.PGAv.1.6.scaffold86.20 | *Capsicum annuum* | eudicot |
| Type II (MIKC) | MIKC*-P | Cg2g017750.1 | *Citrus grandis* | eudicot |
| Type II (MIKC) | MIKC*-P | Cg4g018690.1 | *Citrus grandis* | eudicot |
| Type II (MIKC) | MIKC*-P | Cg5g010340.1 | *Citrus grandis* | eudicot |
| Type II (MIKC) | MIKC*-P | Cg7g021310.1 | *Citrus grandis* | eudicot |
| Type II (MIKC) | MIKC*-P | GSVIVT01007989001 | *Vitis vinifera* | eudicot |
| Type II (MIKC) | MIKC*-P | GSVIVT01035477001 | *Vitis vinifera* | eudicot |
| Type II (MIKC) | MIKC*-P | XP_008393170.1 | *Malus domestica* | eudicot |
| Type II (MIKC) | MIKC*-P | XP_009339081.1 | *Pyrus x bretschneideri* | eudicot |
| Type II (MIKC) | MIKC*-P | XP_009360324.1 | *Pyrus x bretschneideri* | eudicot |
| Type II (MIKC) | MIKC*-P | XP_009369280.1 | *Pyrus x bretschneideri* | eudicot |
| Type II (MIKC) | MIKC*-P | XP_017178947.1 | *Malus domestica* | eudicot |
| Type II (MIKC) | MIKC*-P | XP_017188517.1 | *Malus domestica* | eudicot |
| Type II (MIKC) | MIKC*-P | XP_017192222.1 | *Malus domestica* | eudicot |
| Type II (MIKC) | MIKC*-P | Aco013736.1 | *Ananas comosus* | monocot |
| Type II (MIKC) | MIKC*-P | Bradi4g11097.1 | *Brachypodium distachyon* | monocot |
| Type II (MIKC) | MIKC*-P | Dof000972 ( DoAGL7 ) | *Dendrobium officinale* | monocot |
| Type II (MIKC) | MIKC*-P | Dof002732 ( DoAGL8 ) | *Dendrobium officinale* | monocot |
| Type II (MIKC) | MIKC*-P | GRMZM2G441115_P01 | *Zea mays* | monocot |
| Type II (MIKC) | MIKC*-P | LOC_Os11g43740.1 | *Oryza sativa* | monocot |
| Type II (MIKC) | MIKC*-P | LOC110021454 ( PeAGL3 ) | *Phalaenopsis equestris* | monocot |
| Type II (MIKC) | MIKC*-P | LOC110024229 ( PeAGL2 ) | *Phalaenopsis equestris* | monocot |
| Type II (MIKC) | MIKC*-P | LOC110037644 ( PeAGL4 ) | *Phalaenopsis equestris* | monocot |
| Type II (MIKC) | MIKC*-P | Ma02_p03680.1 | *Musa acuminata* | monocot |
| Type II (MIKC) | MIKC*-P | Ma03_p25430.1 | *Musa acuminata* | monocot |
| Type II (MIKC) | MIKC*-P | Ma11_p12730.1 | *Musa acuminata* | monocot |
| Type II (MIKC) | MIKC*-P | Sb05g025970.1 | *Sorghum bicolor* | monocot |
| Type II (MIKC) | MIKC*-P | TRIUR3_33223 | *Triticum aestivum* | monocot |
| Type II (MIKC) | MIKC*-S | evm_27.model.AmTr_v1.0_scaffold00010.217 | *Amborella trichopoda* | basal angiosperm |
| Type II (MIKC) | MIKC*-S | NNU_13679-RA | *Nelumbo nucifera* | basal angiosperm |
| Type II (MIKC) | MIKC*-S | EDQ53329.1 | *Physcomitrella patens* | bryophyte |
| Type II (MIKC) | MIKC*-S | EDQ55013.1 | *Physcomitrella patens* | bryophyte |
| Type II (MIKC) | MIKC*-S | EDQ61678.1 | *Physcomitrella patens* | bryophyte |
| Type II (MIKC) | MIKC*-S | EDQ61679.1 | *Physcomitrella patens* | bryophyte |
| Type II (MIKC) | MIKC*-S | EDQ65352.1 | *Physcomitrella patens* | bryophyte |
| Type II (MIKC) | MIKC*-S | EDQ68023.1 | *Physcomitrella patens* | bryophyte |
| Type II (MIKC) | MIKC*-S | EDQ72729.1 | *Physcomitrella patens* | bryophyte |
| Type II (MIKC) | MIKC*-S | EDQ72730.1 | *Physcomitrella patens* | bryophyte |
| Type II (MIKC) | MIKC*-S | EDQ76444.1 | *Physcomitrella patens* | bryophyte |
| Type II (MIKC) | MIKC*-S | EDQ81860.1 | *Physcomitrella patens* | bryophyte |
| Type II (MIKC) | MIKC*-S | EDQ81862.1 | *Physcomitrella patens* | bryophyte |
| Type II (MIKC) | MIKC*-S | Mapoly0174s0011.1.p | *Marchantia polymorpha* | bryophyte |
| Type II (MIKC) | MIKC*-S | Sphfalx0001s0415.1.p | *Sphagnum fallax* | bryophyte |
| Type II (MIKC) | MIKC*-S | Sphfalx0022s0017.1.p | *Sphagnum fallax* | bryophyte |
| Type II (MIKC) | MIKC*-S | Sphfalx0028s0045.1.p | *Sphagnum fallax* | bryophyte |
| Type II (MIKC) | MIKC*-S | Sphfalx0088s0070.1.p | *Sphagnum fallax* | bryophyte |
| Type II (MIKC) | MIKC*-S | Sphfalx0284s0012.1.p | *Sphagnum fallax* | bryophyte |
| Type II (MIKC) | MIKC*-S | Achn093911 | *Actinidia chinensis* | eudicot |
| Type II (MIKC) | MIKC*-S | Achn161461 | *Actinidia chinensis* | eudicot |
| Type II (MIKC) | MIKC*-S | Achn266541 | *Actinidia chinensis* | eudicot |
| Type II (MIKC) | MIKC*-S | AtAGL104 | *Arabidopsis thaliana* | eudicot |
| Type II (MIKC) | MIKC*-S | AtAGL66 | *Arabidopsis thaliana* | eudicot |
| Type II (MIKC) | MIKC*-S | AtAGL67 | *Arabidopsis thaliana* | eudicot |
| Type II (MIKC) | MIKC*-S | Bra011763 | *Brassica rapa* | eudicot |
| Type II (MIKC) | MIKC*-S | Bra012308 | *Brassica rapa* | eudicot |
| Type II (MIKC) | MIKC*-S | Bra015643 | *Brassica rapa* | eudicot |
| Type II (MIKC) | MIKC*-S | Bra015645 | *Brassica rapa* | eudicot |
| Type II (MIKC) | MIKC*-S | Bra016386 | *Brassica rapa* | eudicot |
| Type II (MIKC) | MIKC*-S | CA.PGAv.1.6.scaffold421.9 | *Capsicum annuum* | eudicot |
| Type II (MIKC) | MIKC*-S | CA.PGAv.1.6.scaffold517.21 | *Capsicum annuum* | eudicot |
| Type II (MIKC) | MIKC*-S | Cg3g015460.1 | *Citrus grandis* | eudicot |
| Type II (MIKC) | MIKC*-S | GSVIVT01000175001 | *Vitis vinifera* | eudicot |
| Type II (MIKC) | MIKC*-S | GSVIVT01008980001 | *Vitis vinifera* | eudicot |
| Type II (MIKC) | MIKC*-S | GSVIVT01014093001 | *Vitis vinifera* | eudicot |
| Type II (MIKC) | MIKC*-S | GSVIVT01022182001 | *Vitis vinifera* | eudicot |
| Type II (MIKC) | MIKC*-S | XP_008356710.1 | *Malus domestica* | eudicot |
| Type II (MIKC) | MIKC*-S | XP_008378057.1 | *Malus domestica* | eudicot |
| Type II (MIKC) | MIKC*-S | XP_009345537.1 | *Pyrus x bretschneideri* | eudicot |
| Type II (MIKC) | MIKC*-S | XP_009354141.1 | *Pyrus x bretschneideri* | eudicot |
| Type II (MIKC) | MIKC*-S | XP_017189222.1 | *Malus domestica* | eudicot |
| Type II (MIKC) | MIKC*-S | XP_018500899.1 | *Pyrus x bretschneideri* | eudicot |
| Type II (MIKC) | MIKC*-S | Gb_31417 | *Ginkgo biloba* | gymnosperm |
| Type II (MIKC) | MIKC*-S | EFJ16272.1 | *Selaginella moellendorffii* | lycophyte |
| Type II (MIKC) | MIKC*-S | EFJ17745.1 | *Selaginella moellendorffii* | lycophyte |
| Type II (MIKC) | MIKC*-S | EFJ20500.1 | *Selaginella moellendorffii* | lycophyte |
| Type II (MIKC) | MIKC*-S | EFJ24304.1 | *Selaginella moellendorffii* | lycophyte |
| Type II (MIKC) | MIKC*-S | EFJ24305.1 | *Selaginella moellendorffii* | lycophyte |
| Type II (MIKC) | MIKC*-S | EFJ28572.1 | *Selaginella moellendorffii* | lycophyte |
| Type II (MIKC) | MIKC*-S | EFJ35566.1 | *Selaginella moellendorffii* | lycophyte |
| Type II (MIKC) | MIKC*-S | Bradi3g39177.1 | *Brachypodium distachyon* | monocot |
| Type II (MIKC) | MIKC*-S | Dof016198 ( DoAGL3 ) | *Dendrobium officinale* | monocot |
| Type II (MIKC) | MIKC*-S | Dof016199 ( DoAGL6 ) | *Dendrobium officinale* | monocot |
| Type II (MIKC) | MIKC*-S | Dof016201 ( DoAGL5 ) | *Dendrobium officinale* | monocot |
| Type II (MIKC) | MIKC*-S | Dof027465 ( DoAGL1 ) | *Dendrobium officinale* | monocot |
| Type II (MIKC) | MIKC*-S | Dof027466 ( DoAGL2 ) | *Dendrobium officinale* | monocot |
| Type II (MIKC) | MIKC*-S | Dof028043 ( DoAGL4 ) | *Dendrobium officinale* | monocot |
| Type II (MIKC) | MIKC*-S | GRMZM2G152415_P01 | *Zea mays* | monocot |
| Type II (MIKC) | MIKC*-S | GRMZM2G334225_P01 | *Zea mays* | monocot |
| Type II (MIKC) | MIKC*-S | LOC_Os06g11970.1 | *Oryza sativa* | monocot |
| Type II (MIKC) | MIKC*-S | LOC_Os08g38590.1 | *Oryza sativa* | monocot |
| Type II (MIKC) | MIKC*-S | LOC110024981 ( PeAGL1 ) | *Phalaenopsis equestris* | monocot |
| Type II (MIKC) | MIKC*-S | Ma03_p04320.1 | *Musa acuminata* | monocot |
| Type II (MIKC) | MIKC*-S | Ma09_p12630.1 | *Musa acuminata* | monocot |
| Type II (MIKC) | MIKC*-S | Sb10g007810.1 | *Sorghum bicolor* | monocot |
| Type II (MIKC) | MIKC*-S | TRIUR3_20588 | *Triticum aestivum* | monocot |
| Type I |  | Gb_12586 | *Ginkgo biloba* | gymnosperm |
| Type I |  | Gb_21526 | *Ginkgo biloba* | gymnosperm |
| Type I |  | Gb_19258 | *Ginkgo biloba* | gymnosperm |
| Type I |  | Gb_37613 | *Ginkgo biloba* | gymnosperm |
| Type I |  | Gb_40092 | *Ginkgo biloba* | gymnosperm |
| Type I |  | Gb_38883 | *Ginkgo biloba* | gymnosperm |
| Type I |  | Gb_05359 | *Ginkgo biloba* | gymnosperm |
| Type I |  | Gb_33168 | *Ginkgo biloba* | gymnosperm |
| Type I |  | LOC110026675 | *Phalaenopsis equestris* | monocot |
| Type I |  | LOC110027440 | *Phalaenopsis equestris* | monocot |
| Type I |  | LOC110032171 | *Phalaenopsis equestris* | monocot |
| Type I |  | LOC110032184 | *Phalaenopsis equestris* | monocot |
| Type I |  | LOC110039147 | *Phalaenopsis equestris* | monocot |
| Type I |  | LOC110029986 | *Phalaenopsis equestris* | monocot |
| Type I |  | LOC110036449 | *Phalaenopsis equestris* | monocot |
| Type I |  | LOC110036539 | *Phalaenopsis equestris* | monocot |
| Type I |  | LOC110036540 | *Phalaenopsis equestris* | monocot |
| Type I |  | LOC110021912 | *Phalaenopsis equestris* | monocot |
| Type I |  | LOC110034741 | *Phalaenopsis equestris* | monocot |
| Type I |  | LOC110038006 | *Phalaenopsis equestris* | monocot |
| Type I |  | LOC110030987 | *Phalaenopsis equestris* | monocot |
| Type I |  | LOC110020407 | *Phalaenopsis equestris* | monocot |
| Type I |  | Sphfalx0041s0122.1.p | *Sphagnum fallax* | bryophyte |
| Type I |  | Sphfalx0099s0066.1.p | *Sphagnum fallax* | bryophyte |
| Type I |  | Sphfalx0016s0161.1.p | *Sphagnum fallax* | bryophyte |
| Type I |  | Achn147221 | *Actinidia chinensis* | eudicot |
| Type I |  | Achn253421 | *Actinidia chinensis* | eudicot |
| Type I |  | Achn253671 | *Actinidia chinensis* | eudicot |
| Type I |  | Achn147241 | *Actinidia chinensis* | eudicot |
| Type I |  | Achn231041 | *Actinidia chinensis* | eudicot |
| Type I |  | Achn339031 | *Actinidia chinensis* | eudicot |
| Type I |  | Achn253691 | *Actinidia chinensis* | eudicot |
| Type I |  | Achn270051 | *Actinidia chinensis* | eudicot |
| Type I |  | Achn133701 | *Actinidia chinensis* | eudicot |
| Type I |  | Achn196031 | *Actinidia chinensis* | eudicot |
| Type I |  | Achn241031 | *Actinidia chinensis* | eudicot |
| Type I |  | Achn270061 | *Actinidia chinensis* | eudicot |
| Type I |  | Achn177431 | *Actinidia chinensis* | eudicot |
| Type I |  | Achn206911 | *Actinidia chinensis* | eudicot |
| Type I |  | Achn206901 | *Actinidia chinensis* | eudicot |
| Type I |  | Achn206921 | *Actinidia chinensis* | eudicot |
| Type I |  | Achn231961 | *Actinidia chinensis* | eudicot |
| Type I |  | Achn061601 | *Actinidia chinensis* | eudicot |
| Type I |  | Achn184241 | *Actinidia chinensis* | eudicot |
| Type I |  | Achn058181 | *Actinidia chinensis* | eudicot |
| Type I |  | Achn255771 | *Actinidia chinensis* | eudicot |
| Type I |  | Achn339461 | *Actinidia chinensis* | eudicot |
| Type I |  | Achn185551 | *Actinidia chinensis* | eudicot |
| Type I |  | Achn112951 | *Actinidia chinensis* | eudicot |
| Type I |  | Achn275811 | *Actinidia chinensis* | eudicot |
| Type I |  | Achn015591 | *Actinidia chinensis* | eudicot |
| Type I |  | Achn105761 | *Actinidia chinensis* | eudicot |
| Type I |  | Achn141941 | *Actinidia chinensis* | eudicot |
| Type I |  | Achn215281 | *Actinidia chinensis* | eudicot |
| Type I |  | evm_27.model.AmTr_v1.0_scaffold00025.385 | *Amborella trichopoda* | basal angiosperm |
| Type I |  | evm_27.model.AmTr_v1.0_scaffold00140.17 | *Amborella trichopoda* | basal angiosperm |
| Type I |  | evm_27.model.AmTr_v1.0_scaffold00159.17 | *Amborella trichopoda* | basal angiosperm |
| Type I |  | evm_27.model.AmTr_v1.0_scaffold00159.19 | *Amborella trichopoda* | basal angiosperm |
| Type I |  | evm_27.model.AmTr_v1.0_scaffold00159.18 | *Amborella trichopoda* | basal angiosperm |
| Type I |  | evm_27.model.AmTr_v1.0_scaffold00050.92 | *Amborella trichopoda* | basal angiosperm |
| Type I |  | evm_27.model.AmTr_v1.0_scaffold00116.30 | *Amborella trichopoda* | basal angiosperm |
| Type I |  | evm_27.model.AmTr_v1.0_scaffold00022.374 | *Amborella trichopoda* | basal angiosperm |
| Type I |  | evm_27.model.AmTr_v1.0_scaffold00025.268 | *Amborella trichopoda* | basal angiosperm |
| Type I |  | evm_27.model.AmTr_v1.0_scaffold00025.271 | *Amborella trichopoda* | basal angiosperm |
| Type I |  | evm_27.model.AmTr_v1.0_scaffold00176.19 | *Amborella trichopoda* | basal angiosperm |
| Type I |  | evm_27.model.AmTr_v1.0_scaffold00095.150 | *Amborella trichopoda* | basal angiosperm |
| Type I |  | Aco008623.1 | *Ananas comosus* | monocot |
| Type I |  | Aco004987.1 | *Ananas comosus* | monocot |
| Type I |  | Aco004988.1 | *Ananas comosus* | monocot |
| Type I |  | Aco008435.1 | *Ananas comosus* | monocot |
| Type I |  | Aco013324.1 | *Ananas comosus* | monocot |
| Type I |  | Aco011677.1 | *Ananas comosus* | monocot |
| Type I |  | Aco011374.1 | *Ananas comosus* | monocot |
| Type I |  | Aco028086.1 | *Ananas comosus* | monocot |
| Type I |  | Aco022101.1 | *Ananas comosus* | monocot |
| Type I |  | Aco027629.1 | *Ananas comosus* | monocot |
| Type I |  | Aco013644.1 | *Ananas comosus* | monocot |
| Type I |  | AtAGL28 | *Arabidopsis thaliana* | eudicot |
| Type I |  | AtAGL23 | *Arabidopsis thaliana* | eudicot |
| Type I |  | AtAGL40 | *Arabidopsis thaliana* | eudicot |
| Type I |  | AtAGL62 | *Arabidopsis thaliana* | eudicot |
| Type I |  | AtAGL61 | *Arabidopsis thaliana* | eudicot |
| Type I |  | AtAGL60 | *Arabidopsis thaliana* | eudicot |
| Type I |  | AtAGL100 | *Arabidopsis thaliana* | eudicot |
| Type I |  | AtAGL56 | *Arabidopsis thaliana* | eudicot |
| Type I |  | AtAGL55 | *Arabidopsis thaliana* | eudicot |
| Type I |  | AtAGL97 | *Arabidopsis thaliana* | eudicot |
| Type I |  | AtAGL99 | *Arabidopsis thaliana* | eudicot |
| Type I |  | AtAGL83 | *Arabidopsis thaliana* | eudicot |
| Type I |  | AtAGL73 | *Arabidopsis thaliana* | eudicot |
| Type I |  | AtAGL84 | *Arabidopsis thaliana* | eudicot |
| Type I |  | AtAGL58 | *Arabidopsis thaliana* | eudicot |
| Type I |  | AtAGL59 | *Arabidopsis thaliana* | eudicot |
| Type I |  | AtAGL64 | *Arabidopsis thaliana* | eudicot |
| Type I |  | AtAGL85 | *Arabidopsis thaliana* | eudicot |
| Type I |  | AtAGL57 | *Arabidopsis thaliana* | eudicot |
| Type I |  | AtAGL91 | *Arabidopsis thaliana* | eudicot |
| Type I |  | AtAGL29 | *Arabidopsis thaliana* | eudicot |
| Type I |  | ATAGL102 | *Arabidopsis thaliana* | eudicot |
| Type I |  | AtAGL39 | *Arabidopsis thaliana* | eudicot |
| Type I |  | AtAGL74 | *Arabidopsis thaliana* | eudicot |
| Type I |  | AtAGL50 | *Arabidopsis thaliana* | eudicot |
| Type I |  | AtAGL49 | *Arabidopsis thaliana* | eudicot |
| Type I |  | AtAGL43 | *Arabidopsis thaliana* | eudicot |
| Type I |  | AtAGL75 | *Arabidopsis thaliana* | eudicot |
| Type I |  | AtAGL76 | *Arabidopsis thaliana* | eudicot |
| Type I |  | AtAGL77 | *Arabidopsis thaliana* | eudicot |
| Type I |  | AtAGL105 | *Arabidopsis thaliana* | eudicot |
| Type I |  | AtAGL81 | *Arabidopsis thaliana* | eudicot |
| Type I |  | AtAGL98 | *Arabidopsis thaliana* | eudicot |
| Type I |  | AtAGL51 | *Arabidopsis thaliana* | eudicot |
| Type I |  | AtAGL52 | *Arabidopsis thaliana* | eudicot |
| Type I |  | AtAGL78 | *Arabidopsis thaliana* | eudicot |
| Type I |  | AtAGL103 | *Arabidopsis thaliana* | eudicot |
| Type I |  | AtAGL93 | *Arabidopsis thaliana* | eudicot |
| Type I |  | AtAGL53 | *Arabidopsis thaliana* | eudicot |
| Type I |  | AtAGL54 | *Arabidopsis thaliana* | eudicot |
| Type I |  | AtAGL89 | *Arabidopsis thaliana* | eudicot |
| Type I |  | AtAGL107 | *Arabidopsis thaliana* | eudicot |
| Type I |  | AtAGL26 | *Arabidopsis thaliana* | eudicot |
| Type I |  | AtAGL101 | *Arabidopsis thaliana* | eudicot |
| Type I |  | AtAGL82 | *Arabidopsis thaliana* | eudicot |
| Type I |  | AtAGL47 | *Arabidopsis thaliana* | eudicot |
| Type I |  | AtAGL87 | *Arabidopsis thaliana* | eudicot |
| Type I |  | AtAGL48 | *Arabidopsis thaliana* | eudicot |
| Type I |  | AtAGL96 | *Arabidopsis thaliana* | eudicot |
| Type I |  | AtAGL35 | *Arabidopsis thaliana* | eudicot |
| Type I |  | AtAGL36 | *Arabidopsis thaliana* | eudicot |
| Type I |  | AtAGL90 | *Arabidopsis thaliana* | eudicot |
| Type I |  | AtAGL34 | *Arabidopsis thaliana* | eudicot |
| Type I |  | AtAGL38 | *Arabidopsis thaliana* | eudicot |
| Type I |  | AtPHE1 | *Arabidopsis thaliana* | eudicot |
| Type I |  | AtAGL92 | *Arabidopsis thaliana* | eudicot |
| Type I |  | AtAGL86 | *Arabidopsis thaliana* | eudicot |
| Type I |  | AtAGL108 | *Arabidopsis thaliana* | eudicot |
| Type I |  | AtAGL46 | *Arabidopsis thaliana* | eudicot |
| Type I |  | AtAGL45 | *Arabidopsis thaliana* | eudicot |
| Type I |  | AtAGL106 | *Arabidopsis thaliana* | eudicot |
| Type I |  | AtAGL80 | *Arabidopsis thaliana* | eudicot |
| Type I |  | Bradi3g18670.1 | *Brachypodium distachyon* | monocot |
| Type I |  | Bradi4g39420.1 | *Brachypodium distachyon* | monocot |
| Type I |  | Bradi3g04880.1 | *Brachypodium distachyon* | monocot |
| Type I |  | Bradi1g20090.1 | *Brachypodium distachyon* | monocot |
| Type I |  | Bradi2g26320.1 | *Brachypodium distachyon* | monocot |
| Type I |  | Bradi1g58100.1 | *Brachypodium distachyon* | monocot |
| Type I |  | Bradi1g57870.1 | *Brachypodium distachyon* | monocot |
| Type I |  | Bradi1g57410.1 | *Brachypodium distachyon* | monocot |
| Type I |  | Bradi1g57420.1 | *Brachypodium distachyon* | monocot |
| Type I |  | Bradi1g27900.1 | *Brachypodium distachyon* | monocot |
| Type I |  | Bradi2g43290.1 | *Brachypodium distachyon* | monocot |
| Type I |  | Bradi1g39927.1 | *Brachypodium distachyon* | monocot |
| Type I |  | Bradi2g30530.1 | *Brachypodium distachyon* | monocot |
| Type I |  | Bradi1g15480.1 | *Brachypodium distachyon* | monocot |
| Type I |  | Bra011938 | *Brassica rapa* | eudicot |
| Type I |  | Bra035685 | *Brassica rapa* | eudicot |
| Type I |  | Bra002480 | *Brassica rapa* | eudicot |
| Type I |  | Bra020242 | *Brassica rapa* | eudicot |
| Type I |  | Bra020247 | *Brassica rapa* | eudicot |
| Type I |  | Bra022434 | *Brassica rapa* | eudicot |
| Type I |  | Bra038225 | *Brassica rapa* | eudicot |
| Type I |  | Bra026764 | *Brassica rapa* | eudicot |
| Type I |  | Bra032057 | *Brassica rapa* | eudicot |
| Type I |  | Bra007829 | *Brassica rapa* | eudicot |
| Type I |  | Bra003884 | *Brassica rapa* | eudicot |
| Type I |  | Bra026674 | *Brassica rapa* | eudicot |
| Type I |  | Bra027116 | *Brassica rapa* | eudicot |
| Type I |  | Bra040965 | *Brassica rapa* | eudicot |
| Type I |  | Bra009436 | *Brassica rapa* | eudicot |
| Type I |  | Bra014217 | *Brassica rapa* | eudicot |
| Type I |  | Bra035448 (Bra035449) | *Brassica rapa* | eudicot |
| Type I |  | Bra018727 | *Brassica rapa* | eudicot |
| Type I |  | Bra033492 | *Brassica rapa* | eudicot |
| Type I |  | Bra010027 | *Brassica rapa* | eudicot |
| Type I |  | Bra037434 | *Brassica rapa* | eudicot |
| Type I |  | Bra032347 | *Brassica rapa* | eudicot |
| Type I |  | Bra031945 | *Brassica rapa* | eudicot |
| Type I |  | Bra037759 | *Brassica rapa* | eudicot |
| Type I |  | Bra040149 | *Brassica rapa* | eudicot |
| Type I |  | Bra001209 | *Brassica rapa* | eudicot |
| Type I |  | Bra021910 | *Brassica rapa* | eudicot |
| Type I |  | Bra020600 | *Brassica rapa* | eudicot |
| Type I |  | Bra025609 | *Brassica rapa* | eudicot |
| Type I |  | Bra025607 | *Brassica rapa* | eudicot |
| Type I |  | Bra025619 | *Brassica rapa* | eudicot |
| Type I |  | Bra031864 | *Brassica rapa* | eudicot |
| Type I |  | Bra022341 | *Brassica rapa* | eudicot |
| Type I |  | Bra037571 | *Brassica rapa* | eudicot |
| Type I |  | Bra004071 | *Brassica rapa* | eudicot |
| Type I |  | Bra007138 | *Brassica rapa* | eudicot |
| Type I |  | Bra028020 | *Brassica rapa* | eudicot |
| Type I |  | Bra028019 | *Brassica rapa* | eudicot |
| Type I |  | Bra018741 | *Brassica rapa* | eudicot |
| Type I |  | Bra018767 | *Brassica rapa* | eudicot |
| Type I |  | Bra015129 | *Brassica rapa* | eudicot |
| Type I |  | Bra020923 | *Brassica rapa* | eudicot |
| Type I |  | Bra002611 | *Brassica rapa* | eudicot |
| Type I |  | Bra028965 | *Brassica rapa* | eudicot |
| Type I |  | Bra012335 | *Brassica rapa* | eudicot |
| Type I |  | Bra024521 | *Brassica rapa* | eudicot |
| Type I |  | Bra034809 | *Brassica rapa* | eudicot |
| Type I |  | Bra028730 | *Brassica rapa* | eudicot |
| Type I |  | Bra009176 | *Brassica rapa* | eudicot |
| Type I |  | Bra009199 | *Brassica rapa* | eudicot |
| Type I |  | Bra009174 | *Brassica rapa* | eudicot |
| Type I |  | Bra020524 | *Brassica rapa* | eudicot |
| Type I |  | Bra020525 | *Brassica rapa* | eudicot |
| Type I |  | Bra020550 | *Brassica rapa* | eudicot |
| Type I |  | Bra020552 | *Brassica rapa* | eudicot |
| Type I |  | Bra020555 | *Brassica rapa* | eudicot |
| Type I |  | Bra009913 | *Brassica rapa* | eudicot |
| Type I |  | Bra009911 | *Brassica rapa* | eudicot |
| Type I |  | Bra005166 | *Brassica rapa* | eudicot |
| Type I |  | Bra040248 | *Brassica rapa* | eudicot |
| Type I |  | Bra039404 | *Brassica rapa* | eudicot |
| Type I |  | Bra018490 | *Brassica rapa* | eudicot |
| Type I |  | Bra029469 | *Brassica rapa* | eudicot |
| Type I |  | Bra041022 | *Brassica rapa* | eudicot |
| Type I |  | CA.PGAv.1.6.scaffold2165.1 | *Capsicum annuum* | eudicot |
| Type I |  | CA.PGAv.1.6.scaffold2433.1 | *Capsicum annuum* | eudicot |
| Type I |  | CA.PGAv.1.6.scaffold2199.1 | *Capsicum annuum* | eudicot |
| Type I |  | CA.PGAv.1.6.scaffold2165.2 | *Capsicum annuum* | eudicot |
| Type I |  | CA.PGAv.1.6.scaffold4508.1 | *Capsicum annuum* | eudicot |
| Type I |  | CA.PGAv.1.6.scaffold1467.3 | *Capsicum annuum* | eudicot |
| Type I |  | CA.PGAv.1.6.scaffold2471.2 | *Capsicum annuum* | eudicot |
| Type I |  | CA.PGAv.1.6.scaffold2637.1 | *Capsicum annuum* | eudicot |
| Type I |  | CA.PGAv.1.6.scaffold2165.3 | *Capsicum annuum* | eudicot |
| Type I |  | CA.PGAv.1.6.scaffold606.84 | *Capsicum annuum* | eudicot |
| Type I |  | CA.PGAv.1.6.scaffold2433.2 | *Capsicum annuum* | eudicot |
| Type I |  | CA.PGAv.1.6.scaffold2471.1 | *Capsicum annuum* | eudicot |
| Type I |  | CA.PGAv.1.6.scaffold322.8 | *Capsicum annuum* | eudicot |
| Type I |  | CA.PGAv.1.6.scaffold606.85 | *Capsicum annuum* | eudicot |
| Type I |  | CA.PGAv.1.6.scaffold2199.2 | *Capsicum annuum* | eudicot |
| Type I |  | CA.PGAv.1.6.scaffold504.12 | *Capsicum annuum* | eudicot |
| Type I |  | CA.PGAv.1.6.scaffold504.13 | *Capsicum annuum* | eudicot |
| Type I |  | CA.PGAv.1.6.scaffold504.2 | *Capsicum annuum* | eudicot |
| Type I |  | CA.PGAv.1.6.scaffold504.1 | *Capsicum annuum* | eudicot |
| Type I |  | CA.PGAv.1.6.scaffold504.11 | *Capsicum annuum* | eudicot |
| Type I |  | CA.PGAv.1.6.scaffold504.4 | *Capsicum annuum* | eudicot |
| Type I |  | CA.PGAv.1.6.scaffold730.7 | *Capsicum annuum* | eudicot |
| Type I |  | CA.PGAv.1.6.scaffold730.20 | *Capsicum annuum* | eudicot |
| Type I |  | CA.PGAv.1.6.scaffold6731.1 | *Capsicum annuum* | eudicot |
| Type I |  | CA.PGAv.1.6.scaffold504.5 | *Capsicum annuum* | eudicot |
| Type I |  | CA.PGAv.1.6.scaffold730.11 | *Capsicum annuum* | eudicot |
| Type I |  | CA.PGAv.1.6.scaffold730.5 | *Capsicum annuum* | eudicot |
| Type I |  | CA.PGAv.1.6.scaffold504.6 | *Capsicum annuum* | eudicot |
| Type I |  | CA.PGAv.1.6.scaffold730.28 | *Capsicum annuum* | eudicot |
| Type I |  | CA.PGAv.1.6.scaffold730.2 | *Capsicum annuum* | eudicot |
| Type I |  | CA.PGAv.1.6.scaffold730.23 | *Capsicum annuum* | eudicot |
| Type I |  | CA.PGAv.1.6.scaffold730.26 | *Capsicum annuum* | eudicot |
| Type I |  | CA.PGAv.1.6.scaffold1940.3 | *Capsicum annuum* | eudicot |
| Type I |  | CA.PGAv.1.6.scaffold1352.8 | *Capsicum annuum* | eudicot |
| Type I |  | CA.PGAv.1.6.scaffold638.76 | *Capsicum annuum* | eudicot |
| Type I |  | CA.PGAv.1.6.scaffold1352.5 | *Capsicum annuum* | eudicot |
| Type I |  | CA.PGAv.1.6.scaffold111.33 | *Capsicum annuum* | eudicot |
| Type I |  | CA.PGAv.1.6.scaffold58.40 | *Capsicum annuum* | eudicot |
| Type I |  | CA.PGAv.1.6.scaffold58.41 | *Capsicum annuum* | eudicot |
| Type I |  | CA.PGAv.1.6.scaffold252.6 | *Capsicum annuum* | eudicot |
| Type I |  | CA.PGAv.1.6.scaffold763.1 | *Capsicum annuum* | eudicot |
| Type I |  | CA.PGAv.1.6.scaffold1290.6 | *Capsicum annuum* | eudicot |
| Type I |  | CA.PGAv.1.6.scaffold438.121 | *Capsicum annuum* | eudicot |
| Type I |  | CA.PGAv.1.6.scaffold1676.10 | *Capsicum annuum* | eudicot |
| Type I |  | CA.PGAv.1.6.scaffold1676.12 | *Capsicum annuum* | eudicot |
| Type I |  | CA.PGAv.1.6.scaffold1676.13 | *Capsicum annuum* | eudicot |
| Type I |  | CA.PGAv.1.6.scaffold714.15 | *Capsicum annuum* | eudicot |
| Type I |  | CA.PGAv.1.6.scaffold1058.19 | *Capsicum annuum* | eudicot |
| Type I |  | CA.PGAv.1.6.scaffold1058.23 | *Capsicum annuum* | eudicot |
| Type I |  | CA.PGAv.1.6.scaffold1058.21 | *Capsicum annuum* | eudicot |
| Type I |  | CA.PGAv.1.6.scaffold1058.17 | *Capsicum annuum* | eudicot |
| Type I |  | CA.PGAv.1.6.scaffold1058.22 | *Capsicum annuum* | eudicot |
| Type I |  | CA.PGAv.1.6.scaffold1178.11 | *Capsicum annuum* | eudicot |
| Type I |  | CA.PGAv.1.6.scaffold134.188 | *Capsicum annuum* | eudicot |
| Type I |  | CA.PGAv.1.6.scaffold134.192 | *Capsicum annuum* | eudicot |
| Type I |  | CA.PGAv.1.6.scaffold134.193 | *Capsicum annuum* | eudicot |
| Type I |  | CA.PGAv.1.6.scaffold134.190 | *Capsicum annuum* | eudicot |
| Type I |  | CA.PGAv.1.6.scaffold730.27 | *Capsicum annuum* | eudicot |
| Type I |  | CA.PGAv.1.6.scaffold730.8 | *Capsicum annuum* | eudicot |
| Type I |  | CA.PGAv.1.6.scaffold730.15 | *Capsicum annuum* | eudicot |
| Type I |  | CA.PGAv.1.6.scaffold730.19 | *Capsicum annuum* | eudicot |
| Type I |  | CA.PGAv.1.6.scaffold730.22 | *Capsicum annuum* | eudicot |
| Type I |  | CA.PGAv.1.6.scaffold730.24 | *Capsicum annuum* | eudicot |
| Type I |  | CA.PGAv.1.6.scaffold1041.22 | *Capsicum annuum* | eudicot |
| Type I |  | CA.PGAv.1.6.scaffold504.7 | *Capsicum annuum* | eudicot |
| Type I |  | CA.PGAv.1.6.scaffold338.45 | *Capsicum annuum* | eudicot |
| Type I |  | CA.PGAv.1.6.scaffold744.6 | *Capsicum annuum* | eudicot |
| Type I |  | CA.PGAv.1.6.scaffold78.119 | *Capsicum annuum* | eudicot |
| Type I |  | CA.PGAv.1.6.scaffold637.9 | *Capsicum annuum* | eudicot |
| Type I |  | CA.PGAv.1.6.scaffold952.1 | *Capsicum annuum* | eudicot |
| Type I |  | CA.PGAv.1.6.scaffold866.34 | *Capsicum annuum* | eudicot |
| Type I |  | CA.PGAv.1.6.scaffold866.37 | *Capsicum annuum* | eudicot |
| Type I |  | CA.PGAv.1.6.scaffold866.40 | *Capsicum annuum* | eudicot |
| Type I |  | CA.PGAv.1.6.scaffold713.15 | *Capsicum annuum* | eudicot |
| Type I |  | CA.PGAv.1.6.scaffold713.23 | *Capsicum annuum* | eudicot |
| Type I |  | CA.PGAv.1.6.scaffold638.39 | *Capsicum annuum* | eudicot |
| Type I |  | CA.PGAv.1.6.scaffold713.14 | *Capsicum annuum* | eudicot |
| Type I |  | CA.PGAv.1.6.scaffold2092.1 | *Capsicum annuum* | eudicot |
| Type I |  | CA.PGAv.1.6.scaffold377.22 | *Capsicum annuum* | eudicot |
| Type I |  | CA.PGAv.1.6.scaffold478.14 | *Capsicum annuum* | eudicot |
| Type I |  | CA.PGAv.1.6.scaffold478.11 | *Capsicum annuum* | eudicot |
| Type I |  | CA.PGAv.1.6.scaffold478.9 | *Capsicum annuum* | eudicot |
| Type I |  | CA.PGAv.1.6.scaffold1038.23 | *Capsicum annuum* | eudicot |
| Type I |  | CA.PGAv.1.6.scaffold900.1 | *Capsicum annuum* | eudicot |
| Type I |  | CA.PGAv.1.6.scaffold1038.25 | *Capsicum annuum* | eudicot |
| Type I |  | CA.PGAv.1.6.scaffold1038.26 | *Capsicum annuum* | eudicot |
| Type I |  | CA.PGAv.1.6.scaffold1038.24 | *Capsicum annuum* | eudicot |
| Type I |  | CA.PGAv.1.6.scaffold1038.27 | *Capsicum annuum* | eudicot |
| Type I |  | CA.PGAv.1.6.scaffold700.2 | *Capsicum annuum* | eudicot |
| Type I |  | CA.PGAv.1.6.scaffold765.41 | *Capsicum annuum* | eudicot |
| Type I |  | CA.PGAv.1.6.scaffold765.44 | *Capsicum annuum* | eudicot |
| Type I |  | CA.PGAv.1.6.scaffold782.17 | *Capsicum annuum* | eudicot |
| Type I |  | CA.PGAv.1.6.scaffold111.13 | *Capsicum annuum* | eudicot |
| Type I |  | CA.PGAv.1.6.scaffold1184.14 | *Capsicum annuum* | eudicot |
| Type I |  | CA.PGAv.1.6.scaffold1392.11 | *Capsicum annuum* | eudicot |
| Type I |  | CA.PGAv.1.6.scaffold843.3 | *Capsicum annuum* | eudicot |
| Type I |  | CA.PGAv.1.6.scaffold843.2 | *Capsicum annuum* | eudicot |
| Type I |  | CA.PGAv.1.6.scaffold843.1 | *Capsicum annuum* | eudicot |
| Type I |  | CA.PGAv.1.6.scaffold248.24 | *Capsicum annuum* | eudicot |
| Type I |  | CA.PGAv.1.6.scaffold337.111 | *Capsicum annuum* | eudicot |
| Type I |  | CA.PGAv.1.6.scaffold337.169 | *Capsicum annuum* | eudicot |
| Type I |  | CA.PGAv.1.6.scaffold798.34 | *Capsicum annuum* | eudicot |
| Type I |  | CA.PGAv.1.6.scaffold651.10 | *Capsicum annuum* | eudicot |
| Type I |  | CA.PGAv.1.6.scaffold651.8 | *Capsicum annuum* | eudicot |
| Type I |  | CA.PGAv.1.6.scaffold226.6 | *Capsicum annuum* | eudicot |
| Type I |  | CA.PGAv.1.6.scaffold651.9 | *Capsicum annuum* | eudicot |
| Type I |  | CA.PGAv.1.6.scaffold855.4 | *Capsicum annuum* | eudicot |
| Type I |  | CA.PGAv.1.6.scaffold1248.14 | *Capsicum annuum* | eudicot |
| Type I |  | CA.PGAv.1.6.scaffold911.28 | *Capsicum annuum* | eudicot |
| Type I |  | Cg8g002500.1 | *Citrus grandis* | eudicot |
| Type I |  | Cg6g004380.1 | *Citrus grandis* | eudicot |
| Type I |  | Cg8g002490.1 | *Citrus grandis* | eudicot |
| Type I |  | Cg1g007150.1 | *Citrus grandis* | eudicot |
| Type I |  | Cg5g022640.1 | *Citrus grandis* | eudicot |
| Type I |  | Cg7g008860.1 | *Citrus grandis* | eudicot |
| Type I |  | Cg4g011590.1 | *Citrus grandis* | eudicot |
| Type I |  | Cg1g013150.1 | *Citrus grandis* | eudicot |
| Type I |  | Cg8g002480.1 | *Citrus grandis* | eudicot |
| Type I |  | Cg2g045270.1 | *Citrus grandis* | eudicot |
| Type I |  | Cg7g004990.1 | *Citrus grandis* | eudicot |
| Type I |  | Cg5g015680.1 | *Citrus grandis* | eudicot |
| Type I |  | Cg9g016630.1 | *Citrus grandis* | eudicot |
| Type I |  | Cg3g006070.1 | *Citrus grandis* | eudicot |
| Type I |  | Cg9g020780.1 | *Citrus grandis* | eudicot |
| Type I |  | Cg2g044600.1 | *Citrus grandis* | eudicot |
| Type I |  | Cg9g016620.1 | *Citrus grandis* | eudicot |
| Type I |  | Cg9g016670.1 | *Citrus grandis* | eudicot |
| Type I |  | Cg9g020700.1 | *Citrus grandis* | eudicot |
| Type I |  | Cg9g020640.1 | *Citrus grandis* | eudicot |
| Type I |  | Cg3g006050.1 | *Citrus grandis* | eudicot |
| Type I |  | Cg9g021170.1 | *Citrus grandis* | eudicot |
| Type I |  | Cg9g020420.1 | *Citrus grandis* | eudicot |
| Type I |  | Cg9g020480.1 | *Citrus grandis* | eudicot |
| Type I |  | Cg9g020450.1 | *Citrus grandis* | eudicot |
| Type I |  | Cg9g020560.1 | *Citrus grandis* | eudicot |
| Type I |  | Cg9g020550.1 | *Citrus grandis* | eudicot |
| Type I |  | Cg9g020430.1 | *Citrus grandis* | eudicot |
| Type I |  | Cg9g020460.1 | *Citrus grandis* | eudicot |
| Type I |  | Cg9g020500.1 | *Citrus grandis* | eudicot |
| Type I |  | Cg9g020610.1 | *Citrus grandis* | eudicot |
| Type I |  | CgUng015450.1 | *Citrus grandis* | eudicot |
| Type I |  | Cg9g020660.1 | *Citrus grandis* | eudicot |
| Type I |  | Cg3g006090.1 | *Citrus grandis* | eudicot |
| Type I |  | Cg9g021200.1 | *Citrus grandis* | eudicot |
| Type I |  | Cg9g016640.1 | *Citrus grandis* | eudicot |
| Type I |  | CgUng015060.1 | *Citrus grandis* | eudicot |
| Type I |  | Cg9g016680.1 | *Citrus grandis* | eudicot |
| Type I |  | Cg9g020650.1 | *Citrus grandis* | eudicot |
| Type I |  | Cg9g020710.1 | *Citrus grandis* | eudicot |
| Type I |  | CgUng017510.1 | *Citrus grandis* | eudicot |
| Type I |  | CgUng017060.1 | *Citrus grandis* | eudicot |
| Type I |  | CgUng017030.1 | *Citrus grandis* | eudicot |
| Type I |  | Cg9g021160.1 | *Citrus grandis* | eudicot |
| Type I |  | Cg3g006060.1 | *Citrus grandis* | eudicot |
| Type I |  | Cg9g021190.1 | *Citrus grandis* | eudicot |
| Type I |  | Cg3g006030.1 | *Citrus grandis* | eudicot |
| Type I |  | CgUng015050.1 | *Citrus grandis* | eudicot |
| Type I |  | Cg9g020620.1 | *Citrus grandis* | eudicot |
| Type I |  | Cg9g020670.1 | *Citrus grandis* | eudicot |
| Type I |  | Cg9g016650.1 | *Citrus grandis* | eudicot |
| Type I |  | Cg9g020690.1 | *Citrus grandis* | eudicot |
| Type I |  | Cg9g016660.1 | *Citrus grandis* | eudicot |
| Type I |  | Cg9g016610.1 | *Citrus grandis* | eudicot |
| Type I |  | CgUng017020.1 | *Citrus grandis* | eudicot |
| Type I |  | Cg9g021180.1 | *Citrus grandis* | eudicot |
| Type I |  | Cg3g006040.1 | *Citrus grandis* | eudicot |
| Type I |  | CgUng017520.1 | *Citrus grandis* | eudicot |
| Type I |  | CgUng017070.1 | *Citrus grandis* | eudicot |
| Type I |  | Cg9g020630.1 | *Citrus grandis* | eudicot |
| Type I |  | Cg2g044610.1 | *Citrus grandis* | eudicot |
| Type I |  | Cg2g029430.1 | *Citrus grandis* | eudicot |
| Type I |  | Cg2g024680.1 | *Citrus grandis* | eudicot |
| Type I |  | Cg6g019190.1 | *Citrus grandis* | eudicot |
| Type I |  | Cg1g026230.1 | *Citrus grandis* | eudicot |
| Type I |  | Cg8g022520.1 | *Citrus grandis* | eudicot |
| Type I |  | Cg9g017620.1 | *Citrus grandis* | eudicot |
| Type I |  | Cg4g003150.1 | *Citrus grandis* | eudicot |
| Type I |  | Cg3g020740.1 | *Citrus grandis* | eudicot |
| Type I |  | Cg6g013970.1 | *Citrus grandis* | eudicot |
| Type I |  | Cg5g020850.1 | *Citrus grandis* | eudicot |
| Type I |  | Cg9g022090.1 | *Citrus grandis* | eudicot |
| Type I |  | Dof007552 | *Dendrobium officinale* | monocot |
| Type I |  | Dof016433 | *Dendrobium officinale* | monocot |
| Type I |  | Dof022131 | *Dendrobium officinale* | monocot |
| Type I |  | Dof019205 | *Dendrobium officinale* | monocot |
| Type I |  | Dof019204 | *Dendrobium officinale* | monocot |
| Type I |  | Dof006778 | *Dendrobium officinale* | monocot |
| Type I |  | Dof014289 | *Dendrobium officinale* | monocot |
| Type I |  | Dof014095 | *Dendrobium officinale* | monocot |
| Type I |  | Dof027941 | *Dendrobium officinale* | monocot |
| Type I |  | Dof006211 | *Dendrobium officinale* | monocot |
| Type I |  | Dof006775 | *Dendrobium officinale* | monocot |
| Type I |  | Dof021833 | *Dendrobium officinale* | monocot |
| Type I |  | Dof021835 | *Dendrobium officinale* | monocot |
| Type I |  | Dof021834 | *Dendrobium officinale* | monocot |
| Type I |  | Dof021832 | *Dendrobium officinale* | monocot |
| Type I |  | Dof027568 | *Dendrobium officinale* | monocot |
| Type I |  | Dof000568 | *Dendrobium officinale* | monocot |
| Type I |  | Dof022549 | *Dendrobium officinale* | monocot |
| Type I |  | Dof025806 | *Dendrobium officinale* | monocot |
| Type I |  | Dof025329 | *Dendrobium officinale* | monocot |
| Type I |  | Dof019717 | *Dendrobium officinale* | monocot |
| Type I |  | Dof028556 | *Dendrobium officinale* | monocot |
| Type I |  | Dof022563 | *Dendrobium officinale* | monocot |
| Type I |  | Dof018025 | *Dendrobium officinale* | monocot |
| Type I |  | Dof005316 | *Dendrobium officinale* | monocot |
| Type I |  | Dof003978 | *Dendrobium officinale* | monocot |
| Type I |  | Dof007676 | *Dendrobium officinale* | monocot |
| Type I |  | XP_008360611.1 | *Malus domestica* | eudicot |
| Type I |  | XP_008364432.1 | *Malus domestica* | eudicot |
| Type I |  | XP_008372978.1 | *Malus domestica* | eudicot |
| Type I |  | XP_008380343.1 | *Malus domestica* | eudicot |
| Type I |  | XP_008380804.1 | *Malus domestica* | eudicot |
| Type I |  | XP_008365694.1 | *Malus domestica* | eudicot |
| Type I |  | XP_008380815.1 | *Malus domestica* | eudicot |
| Type I |  | XP_008342514.1 | *Malus domestica* | eudicot |
| Type I |  | XP_008377434.1 | *Malus domestica* | eudicot |
| Type I |  | XP_008344202.1 | *Malus domestica* | eudicot |
| Type I |  | XP_008385539.1 | *Malus domestica* | eudicot |
| Type I |  | XP_017184430.1 | *Malus domestica* | eudicot |
| Type I |  | XP_017191678.1 | *Malus domestica* | eudicot |
| Type I |  | XP_008350236.1 | *Malus domestica* | eudicot |
| Type I |  | XP_008347434.2 | *Malus domestica* | eudicot |
| Type I |  | XP_008383563.1 | *Malus domestica* | eudicot |
| Type I |  | XP_008371739.2 | *Malus domestica* | eudicot |
| Type I |  | XP_017192865.1 | *Malus domestica* | eudicot |
| Type I |  | XP_008383564.1 | *Malus domestica* | eudicot |
| Type I |  | XP_008371586.1 | *Malus domestica* | eudicot |
| Type I |  | XP_017188224.1 | *Malus domestica* | eudicot |
| Type I |  | XP_017186080.1 | *Malus domestica* | eudicot |
| Type I |  | XP_008374388.1 | *Malus domestica* | eudicot |
| Type I |  | XP_008354940.1 | *Malus domestica* | eudicot |
| Type I |  | XP_017182559.1 | *Malus domestica* | eudicot |
| Type I |  | XP_008380977.1 | *Malus domestica* | eudicot |
| Type I |  | XP_008360624.1 | *Malus domestica* | eudicot |
| Type I |  | XP_008343298.1 | *Malus domestica* | eudicot |
| Type I |  | XP_008362583.1 | *Malus domestica* | eudicot |
| Type I |  | XP_017185485.1 | *Malus domestica* | eudicot |
| Type I |  | XP_008388916.1 | *Malus domestica* | eudicot |
| Type I |  | XP_008355789.1 | *Malus domestica* | eudicot |
| Type I |  | XP_008364016.1 | *Malus domestica* | eudicot |
| Type I |  | XP_008388915.1 | *Malus domestica* | eudicot |
| Type I |  | XP_008373482.1 | *Malus domestica* | eudicot |
| Type I |  | XP_008373484.1 | *Malus domestica* | eudicot |
| Type I |  | XP_008373059.1 | *Malus domestica* | eudicot |
| Type I |  | XP_017188390.1 | *Malus domestica* | eudicot |
| Type I |  | XP_008339033.1 | *Malus domestica* | eudicot |
| Type I |  | XP_008378643.1 | *Malus domestica* | eudicot |
| Type I |  | Ma05_p01890.1 | *Musa acuminata* | monocot |
| Type I |  | Ma04_p34810.1 | *Musa acuminata* | monocot |
| Type I |  | Ma05_p01930.1 | *Musa acuminata* | monocot |
| Type I |  | Ma05_p01940.1 | *Musa acuminata* | monocot |
| Type I |  | Ma05_p28910.1 | *Musa acuminata* | monocot |
| Type I |  | Ma08_p07050.1 | *Musa acuminata* | monocot |
| Type I |  | Ma11_p24130.1 | *Musa acuminata* | monocot |
| Type I |  | Ma03_p26910.1 | *Musa acuminata* | monocot |
| Type I |  | Ma08_p04740.1 | *Musa acuminata* | monocot |
| Type I |  | Ma01_p20740.1 | *Musa acuminata* | monocot |
| Type I |  | Ma09_p06890.1 | *Musa acuminata* | monocot |
| Type I |  | Ma04_p05410.1 | *Musa acuminata* | monocot |
| Type I |  | Ma04_p11870.1 | *Musa acuminata* | monocot |
| Type I |  | Ma06_p26090.1 | *Musa acuminata* | monocot |
| Type I |  | Ma05_p27740.1 | *Musa acuminata* | monocot |
| Type I |  | Ma08_p11420.1 | *Musa acuminata* | monocot |
| Type I |  | Ma03_p08420.1 | *Musa acuminata* | monocot |
| Type I |  | Ma10_p05280.1 | *Musa acuminata* | monocot |
| Type I |  | Ma02_p20230.1 | *Musa acuminata* | monocot |
| Type I |  | Ma11_p16180.1 | *Musa acuminata* | monocot |
| Type I |  | Ma03_p05320.1 | *Musa acuminata* | monocot |
| Type I |  | Ma03_p25760.1 | *Musa acuminata* | monocot |
| Type I |  | Ma08_p06010.1 | *Musa acuminata* | monocot |
| Type I |  | Ma02_p20240.1 | *Musa acuminata* | monocot |
| Type I |  | NNU 05854-RA | *Nelumbo nucifera* | basal angiosperm |
| Type I |  | NNU 25601-RA | *Nelumbo nucifera* | basal angiosperm |
| Type I |  | NNU 25602-RA | *Nelumbo nucifera* | basal angiosperm |
| Type I |  | NNU 25750-RA | *Nelumbo nucifera* | basal angiosperm |
| Type I |  | NNU 04236-RA | *Nelumbo nucifera* | basal angiosperm |
| Type I |  | NNU 03215-RA | *Nelumbo nucifera* | basal angiosperm |
| Type I |  | NNU 09660-RA | *Nelumbo nucifera* | basal angiosperm |
| Type I |  | NNU 17993-RA | *Nelumbo nucifera* | basal angiosperm |
| Type I |  | NNU 24745-RA | *Nelumbo nucifera* | basal angiosperm |
| Type I |  | NNU 09213-RA | *Nelumbo nucifera* | basal angiosperm |
| Type I |  | NNU 08424-RA | *Nelumbo nucifera* | basal angiosperm |
| Type I |  | NNU 08261-RA | *Nelumbo nucifera* | basal angiosperm |
| Type I |  | NNU 09219-RA | *Nelumbo nucifera* | basal angiosperm |
| Type I |  | NNU 13210-RA | *Nelumbo nucifera* | basal angiosperm |
| Type I |  | NNU 08466-RA | *Nelumbo nucifera* | basal angiosperm |
| Type I |  | LOC_Os06g22760.1 | *Oryza sativa* | monocot |
| Type I |  | LOC_Os03g14850.1 | *Oryza sativa* | monocot |
| Type I |  | LOC_Os01g74440.1 | *Oryza sativa* | monocot |
| Type I |  | LOC_Os09g02830.1 | *Oryza sativa* | monocot |
| Type I |  | LOC_Os09g02780.1 | *Oryza sativa* | monocot |
| Type I |  | LOC_Os02g06860.1 | *Oryza sativa* | monocot |
| Type I |  | LOC_Os05g23780.1 | *Oryza sativa* | monocot |
| Type I |  | LOC_Os01g11510.1 | *Oryza sativa* | monocot |
| Type I |  | LOC_Os06g30810.1 | *Oryza sativa* | monocot |
| Type I |  | LOC_Os06g30830.1 | *Oryza sativa* | monocot |
| Type I |  | LOC_Os12g21850.1 | *Oryza sativa* | monocot |
| Type I |  | LOC_Os12g21880.1 | *Oryza sativa* | monocot |
| Type I |  | LOC_Os07g04170.1 | *Oryza sativa* | monocot |
| Type I |  | LOC_Os04g24790.1 | *Oryza sativa* | monocot |
| Type I |  | LOC_Os04g24800.1 | *Oryza sativa* | monocot |
| Type I |  | LOC_Os04g25920.1 | *Oryza sativa* | monocot |
| Type I |  | LOC_Os04g24810.1 | *Oryza sativa* | monocot |
| Type I |  | LOC_Os01g23760.1 | *Oryza sativa* | monocot |
| Type I |  | LOC_Os01g23750.1 | *Oryza sativa* | monocot |
| Type I |  | LOC_Os01g23770.1 | *Oryza sativa* | monocot |
| Type I |  | LOC_Os01g68560.1 | *Oryza sativa* | monocot |
| Type I |  | LOC_Os01g67890.1 | *Oryza sativa* | monocot |
| Type I |  | LOC_Os01g23780.1 | *Oryza sativa* | monocot |
| Type I |  | LOC_Os11g12360.1 | *Oryza sativa* | monocot |
| Type I |  | LOC_Os01g18440.1 | *Oryza sativa* | monocot |
| Type I |  | LOC_Os03g37670.1 | *Oryza sativa* | monocot |
| Type I |  | LOC_Os03g38610.1 | *Oryza sativa* | monocot |
| Type I |  | LOC_Os04g25870.1 | *Oryza sativa* | monocot |
| Type I |  | LOC_Os01g18420.1 | *Oryza sativa* | monocot |
| Type I |  | EDQ68285.1 | *Physcomitrella patens* | bryophyte |
| Type I |  | EDQ69179.1 | *Physcomitrella patens* | bryophyte |
| Type I |  | EDQ81860.1 | *Physcomitrella patens* | bryophyte |
| Type I |  | EDQ59271.1 | *Physcomitrella patens* | bryophyte |
| Type I |  | EDQ59272.1 | *Physcomitrella patens* | bryophyte |
| Type I |  | EDQ59312.1 | *Physcomitrella patens* | bryophyte |
| Type I |  | EDQ59313.1 | *Physcomitrella patens* | bryophyte |
| Type I |  | EDQ70729.1 | *Physcomitrella patens* | bryophyte |
| Type I |  | EDQ65034.1 | *Physcomitrella patens* | bryophyte |
| Type I |  | EDQ75755.1 | *Physcomitrella patens* | bryophyte |
| Type I |  | EDQ80264.1 | *Physcomitrella patens* | bryophyte |
| Type I |  | XP_009358240.1 | *Pyrus x bretschneideri* | eudicot |
| Type I |  | XP_009335377.1 | *Pyrus x bretschneideri* | eudicot |
| Type I |  | XP_009335378.1 | *Pyrus x bretschneideri* | eudicot |
| Type I |  | XP_009375893.1 | *Pyrus x bretschneideri* | eudicot |
| Type I |  | XP_009357941.1 | *Pyrus x bretschneideri* | eudicot |
| Type I |  | XP_009357940.1 | *Pyrus x bretschneideri* | eudicot |
| Type I |  | XP_009335460.1 | *Pyrus x bretschneideri* | eudicot |
| Type I |  | XP_009335453.1 | *Pyrus x bretschneideri* | eudicot |
| Type I |  | XP_009349899.1 | *Pyrus x bretschneideri* | eudicot |
| Type I |  | XP_009362796.1 | *Pyrus x bretschneideri* | eudicot |
| Type I |  | XP_009340228.1 | *Pyrus x bretschneideri* | eudicot |
| Type I |  | XP_009360965.1 | *Pyrus x bretschneideri* | eudicot |
| Type I |  | XP_009360966.1 | *Pyrus x bretschneideri* | eudicot |
| Type I |  | XP_009365775.1 | *Pyrus x bretschneideri* | eudicot |
| Type I |  | XP_009348874.1 | *Pyrus x bretschneideri* | eudicot |
| Type I |  | XP_018500589.1 | *Pyrus x bretschneideri* | eudicot |
| Type I |  | XP_009345855.1 | *Pyrus x bretschneideri* | eudicot |
| Type I |  | XP_009355438.1 | *Pyrus x bretschneideri* | eudicot |
| Type I |  | XP_009354489.1 | *Pyrus x bretschneideri* | eudicot |
| Type I |  | XP_009344134.2 | *Pyrus x bretschneideri* | eudicot |
| Type I |  | XP_009341327.1 | *Pyrus x bretschneideri* | eudicot |
| Type I |  | XP_009343826.1 | *Pyrus x bretschneideri* | eudicot |
| Type I |  | XP_009349916.1 | *Pyrus x bretschneideri* | eudicot |
| Type I |  | XP_009351026.2 | *Pyrus x bretschneideri* | eudicot |
| Type I |  | XP_009365497.1 | *Pyrus x bretschneideri* | eudicot |
| Type I |  | XP_009335561.1 | *Pyrus x bretschneideri* | eudicot |
| Type I |  | XP_018505084.1 | *Pyrus x bretschneideri* | eudicot |
| Type I |  | XP_009365496.1 | *Pyrus x bretschneideri* | eudicot |
| Type I |  | XP_009335562.1 | *Pyrus x bretschneideri* | eudicot |
| Type I |  | XP_009365768.1 | *Pyrus x bretschneideri* | eudicot |
| Type I |  | XP_009365773.1 | *Pyrus x bretschneideri* | eudicot |
| Type I |  | XP_009365769.2 | *Pyrus x bretschneideri* | eudicot |
| Type I |  | XP_009365771.1 | *Pyrus x bretschneideri* | eudicot |
| Type I |  | XP_009376202.1 | *Pyrus x bretschneideri* | eudicot |
| Type I |  | EFJ31163.1 | *Selaginella moellendorffii* | lycophyte |
| Type I |  | EFJ17182.1 | *Selaginella moellendorffii* | lycophyte |
| Type I |  | EFJ13243.1 | *Selaginella moellendorffii* | lycophyte |
| Type I |  | EFJ04345.1 | *Selaginella moellendorffii* | lycophyte |
| Type I |  | EFJ20589.1 | *Selaginella moellendorffii* | lycophyte |
| Type I |  | EFJ28649.1 | *Selaginella moellendorffii* | lycophyte |
| Type I |  | EFJ36791.1 | *Selaginella moellendorffii* | lycophyte |
| Type I |  | EFJ27879.1 | *Selaginella moellendorffii* | lycophyte |
| Type I |  | EFJ19168.1 | *Selaginella moellendorffii* | lycophyte |
| Type I |  | EFJ13561.1 | *Selaginella moellendorffii* | lycophyte |
| Type I |  | EFJ28155.1 | *Selaginella moellendorffii* | lycophyte |
| Type I |  | EFJ29649.1 | *Selaginella moellendorffii* | lycophyte |
| Type I |  | EFJ07769.1 | *Selaginella moellendorffii* | lycophyte |
| Type I |  | EFJ28095.1 | *Selaginella moellendorffii* | lycophyte |
| Type I |  | EFJ28430.1 | *Selaginella moellendorffii* | lycophyte |
| Type I |  | EFJ20449.1 | *Selaginella moellendorffii* | lycophyte |
| Type I |  | EFJ28644.1 | *Selaginella moellendorffii* | lycophyte |
| Type I |  | EFJ20583.1 | *Selaginella moellendorffii* | lycophyte |
| Type I |  | EFJ15402.1 | *Selaginella moellendorffii* | lycophyte |
| Type I |  | EFJ24949.1 | *Selaginella moellendorffii* | lycophyte |
| Type I |  | EFJ26274.1 | *Selaginella moellendorffii* | lycophyte |
| Type I |  | EFJ14572.1 | *Selaginella moellendorffii* | lycophyte |
| Type I |  | EFJ22206.1 | *Selaginella moellendorffii* | lycophyte |
| Type I |  | EFJ25738.1 | *Selaginella moellendorffii* | lycophyte |
| Type I |  | EFJ25544.1 | *Selaginella moellendorffii* | lycophyte |
| Type I |  | EFJ32357.1 | *Selaginella moellendorffii* | lycophyte |
| Type I |  | EFJ20888.1 | *Selaginella moellendorffii* | lycophyte |
| Type I |  | Sb06g015640.1 | *Sorghum bicolor* | monocot |
| Type I |  | Sb02g006730.1 | *Sorghum bicolor* | monocot |
| Type I |  | Sb02g006700.1 | *Sorghum bicolor* | monocot |
| Type I |  | Sb02g006750.1 | *Sorghum bicolor* | monocot |
| Type I |  | Sb02g006710.1 | *Sorghum bicolor* | monocot |
| Type I |  | Sb09g029120.1 | *Sorghum bicolor* | monocot |
| Type I |  | Sb04g004410.1 | *Sorghum bicolor* | monocot |
| Type I |  | Sb02g002400.1 | *Sorghum bicolor* | monocot |
| Type I |  | Sb02g002410.1 | *Sorghum bicolor* | monocot |
| Type I |  | Sb02g002430.1 | *Sorghum bicolor* | monocot |
| Type I |  | Sb03g027580.1 | *Sorghum bicolor* | monocot |
| Type I |  | Sb10g019770.1 | *Sorghum bicolor* | monocot |
| Type I |  | Sb10g002680.1 | *Sorghum bicolor* | monocot |
| Type I |  | Sb09g016220.1 | *Sorghum bicolor* | monocot |
| Type I |  | Sb10g021663.1 | *Sorghum bicolor* | monocot |
| Type I |  | Sb10g019790.1 | *Sorghum bicolor* | monocot |
| Type I |  | Sb02g037430.1 | *Sorghum bicolor* | monocot |
| Type I |  | Sb03g013650.1 | *Sorghum bicolor* | monocot |
| Type I |  | Sb03g043580.1 | *Sorghum bicolor* | monocot |
| Type I |  | Sb09g003030.1 | *Sorghum bicolor* | monocot |
| Type I |  | Sb03g043520.1 | *Sorghum bicolor* | monocot |
| Type I |  | Sb05g023890.1 | *Sorghum bicolor* | monocot |
| Type I |  | Sb05g023910.1 | *Sorghum bicolor* | monocot |
| Type I |  | Sb05g023895.1 | *Sorghum bicolor* | monocot |
| Type I |  | Sb03g011705.1 | *Sorghum bicolor* | monocot |
| Type I |  | TRIUR3_29223 | *Triticum aestivum* | monocot |
| Type I |  | TRIUR3_33847 | *Triticum aestivum* | monocot |
| Type I |  | TRIUR3_18019 | *Triticum aestivum* | monocot |
| Type I |  | TRIUR3_22433 | *Triticum aestivum* | monocot |
| Type I |  | TRIUR3_24172 | *Triticum aestivum* | monocot |
| Type I |  | TRIUR3_08031 | *Triticum aestivum* | monocot |
| Type I |  | TRIUR3_23045 | *Triticum aestivum* | monocot |
| Type I |  | TRIUR3_23044 | *Triticum aestivum* | monocot |
| Type I |  | TRIUR3_30893 | *Triticum aestivum* | monocot |
| Type I |  | TRIUR3_04104 | *Triticum aestivum* | monocot |
| Type I |  | TRIUR3_27828 | *Triticum aestivum* | monocot |
| Type I |  | TRIUR3_06267 | *Triticum aestivum* | monocot |
| Type I |  | TRIUR3_10620 | *Triticum aestivum* | monocot |
| Type I |  | TRIUR3_30689 | *Triticum aestivum* | monocot |
| Type I |  | TRIUR3_26805 | *Triticum aestivum* | monocot |
| Type I |  | TRIUR3_03114 | *Triticum aestivum* | monocot |
| Type I |  | TRIUR3_08220 | *Triticum aestivum* | monocot |
| Type I |  | TRIUR3_33615 | *Triticum aestivum* | monocot |
| Type I |  | TRIUR3_28009 | *Triticum aestivum* | monocot |
| Type I |  | TRIUR3_27063 | *Triticum aestivum* | monocot |
| Type I |  | TRIUR3_34958 | *Triticum aestivum* | monocot |
| Type I |  | TRIUR3_01526 | *Triticum aestivum* | monocot |
| Type I |  | TRIUR3_11075 | *Triticum aestivum* | monocot |
| Type I |  | TRIUR3_26031 | *Triticum aestivum* | monocot |
| Type I |  | TRIUR3_34466 | *Triticum aestivum* | monocot |
| Type I |  | TRIUR3_11471 | *Triticum aestivum* | monocot |
| Type I |  | GSVIVT01037024001 | *Vitis vinifera* | eudicot |
| Type I |  | GSVIVT01037031001 | *Vitis vinifera* | eudicot |
| Type I |  | GSVIVT01037034001 | *Vitis vinifera* | eudicot |
| Type I |  | GSVIVT01010221001 | *Vitis vinifera* | eudicot |
| Type I |  | GSVIVT01037022001 | *Vitis vinifera* | eudicot |
| Type I |  | GSVIVT01037026001 | *Vitis vinifera* | eudicot |
| Type I |  | GSVIVT01006592001 | *Vitis vinifera* | eudicot |
| Type I |  | GSVIVT01021534001 | *Vitis vinifera* | eudicot |
| Type I |  | GSVIVT01010218001 | *Vitis vinifera* | eudicot |
| Type I |  | GSVIVT01031333001 | *Vitis vinifera* | eudicot |
| Type I |  | GRMZM2G459864_P01 | *Zea mays* | monocot |
| Type I |  | GRMZM2G472096_P01 | *Zea mays* | monocot |
| Type I |  | GRMZM2G472100_P01 | *Zea mays* | monocot |
| Type I |  | AC234185.1_FGP006 | *Zea mays* | monocot |
| Type I |  | GRMZM5G891280_P01 | *Zea mays* | monocot |
| Type I |  | GRMZM2G073357_P01 | *Zea mays* | monocot |
| Type I |  | GRMZM2G375707_P01 | *Zea mays* | monocot |
| Type I |  | GRMZM2G099577_P01 | *Zea mays* | monocot |
| Type I |  | GRMZM2G110582_P03 | *Zea mays* | monocot |
| Type I |  | GRMZM2G035092_P01 | *Zea mays* | monocot |
| Type I |  | GRMZM5G878490_P01 | *Zea mays* | monocot |
| Type I |  | GRMZM2G337892_P01 | *Zea mays* | monocot |
| Type I |  | GRMZM2G306610_P01 | *Zea mays* | monocot |
| Type I |  | GRMZM2G453555_P01 | *Zea mays* | monocot |
| Type I |  | GRMZM2G470857_P01 | *Zea mays* | monocot |
| Type I |  | GRMZM2G099408_P01 | *Zea mays* | monocot |
| Type I |  | GRMZM5G839969_P01 | *Zea mays* | monocot |
| Type I |  | GRMZM5G853066_P01 | *Zea mays* | monocot |
| MADS-box |  | Vocar.0002s0667.1.p | *Volvox carteri* | chlorophyte |
| MADS-box |  | Vocar.0014s0224.1.p | *Volvox carteri* | chlorophyte |
| MADS-box |  | 21861 | *Micromonas pusilla* CCMP1545 | chlorophyte |
| MADS-box |  | Cre18.g749550.t1.1 | *Chlamydomonas reinhardtii* | chlorophyte |
