# Supplementary material for: Genome-wide identification and classification of MIKC-type MADS-box genes in Streptophyte lineages and expression analyses to reveal their role in seed germination of orchid
Source: BMC Plant Biol. 2019 May 28;19:223. doi: 10.1186/s12870-019-1836-5 (PMC6540398; doi:10.1186/s12870-019-1836-5)
Supplement: Supplementary file 2 — Figure S1. BLASTP graphic overview of four MADS-box proteins from chlorophytes based on the National Center for Biotechnology Information (NCBI) database. (A) MADS-box protein (Cre18.g749550.t1.1) from Chlamydomonas reinhardtii. (B) and (C) MADS-box proteins (Vocar.0002 s0667.1.p and Vocar.0014 s0224.1.p) from Volvox carteri. (D) MADS-box protein (21861) from Micromonas pusilla CCMP1545. (DOCX 155 kb) [file 12870_2019_1836_MOESM2_ESM.docx]

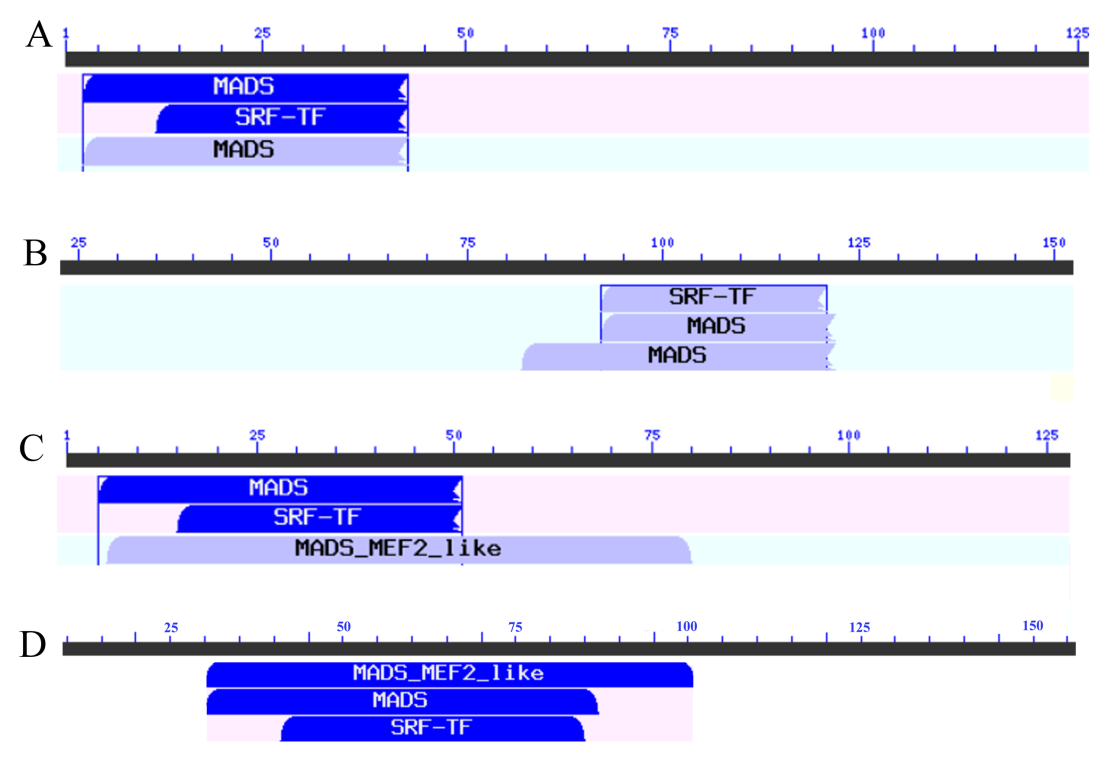


Figure S1 BLASTP graphic overview of four MADS-box proteins from chlorophytes based on the National Center for Biotechnology Information (NCBI) database. (A) MADS-box protein (Cre18.g749550.t1.1) from *Chlamydomonas reinhardtii*. (B) and (C) MADS-box proteins (Vocar.0002s0667.1.p and Vocar.0014s0224.1.p) from *Volvox carteri*. (D) MADS-box protein (21861) from *Micromonas pusilla* CCMP1545.
