# Supplementary material for: Genome-wide identification and classification of MIKC-type MADS-box genes in Streptophyte lineages and expression analyses to reveal their role in seed germination of orchid
Source: BMC Plant Biol. 2019 May 28;19:223. doi: 10.1186/s12870-019-1836-5 (PMC6540398; doi:10.1186/s12870-019-1836-5)
Supplement: Supplementary file 3 — Figure S2. Alignment of the amino acid sequences of only one MADS-box gene (GAQ89767.1) from Klebsormidium nitens and AtSVP (AT2G22540.1) and AtAGL24 (AT4G24540.1) of the SVP subfamily, as well as AtAGL65 (AT1G18750.1) and AtAGL66 (AT1G77980.1) of MIKC* genes from Arabidopsis thaliana. The GAQ89767.1 protein contains M-, I, K- and C domains, which are indicated by red, yellow, green and blue boxes, respectively. (DOCX 1228 kb) [file 12870_2019_1836_MOESM3_ESM.docx]

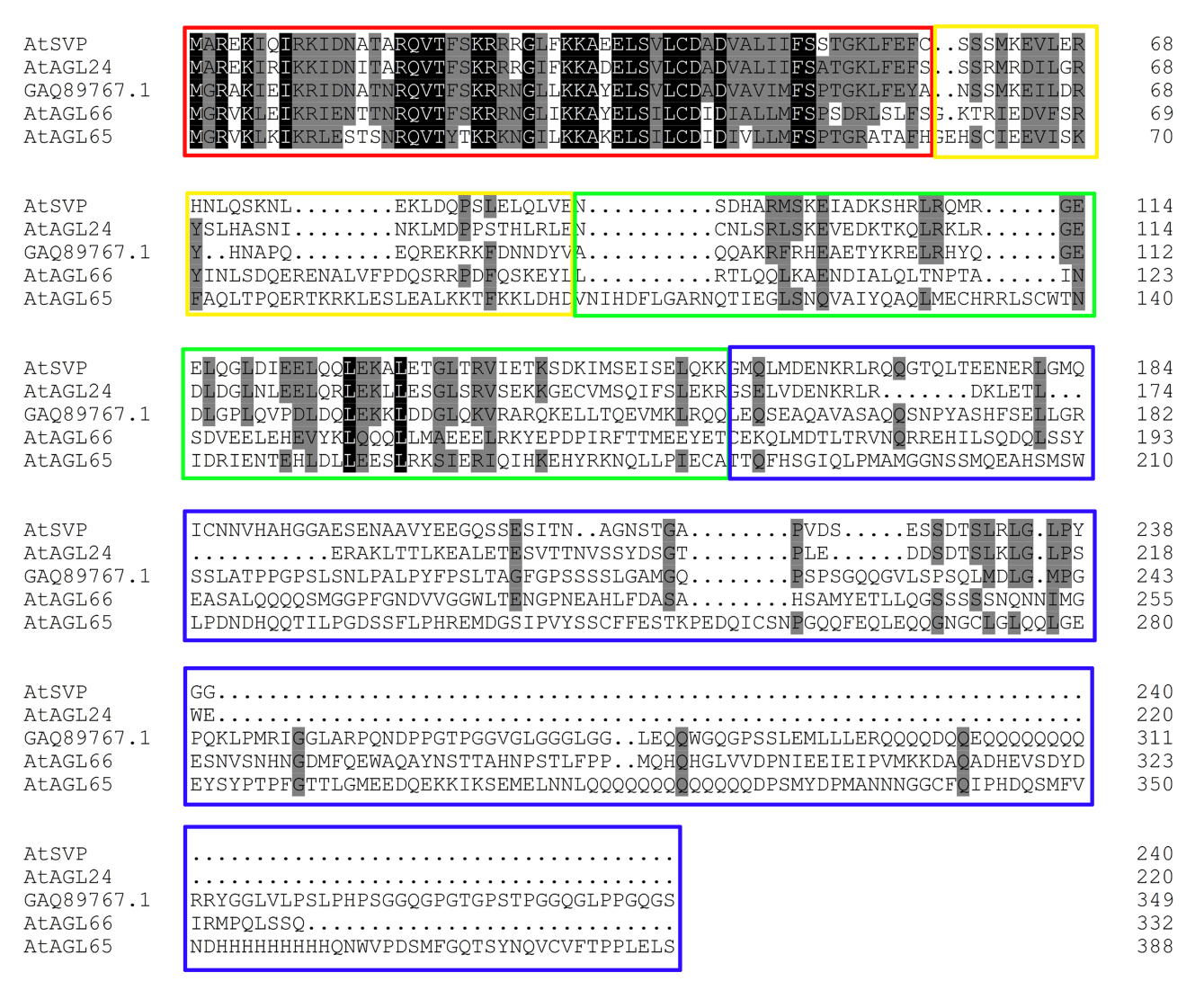


Figure S2 Alignment of the amino acid sequences of only one MADS-box gene (GAQ89767.1) from *Klebsormidium nitens* and AtSVP (AT2G22540.1) and AtAGL24 (AT4G24540.1) of the SVP subfamily, as well as AtAGL65 (AT1G18750.1) and AtAGL66 (AT1G77980.1) of MIKC* genes from *Arabidopsis thaliana*. The GAQ89767.1 protein contains M-, I, K- and C domains, which are indicated by red, yellow, green and blue boxes, respectively.
