# Supplementary material for: Genome-wide identification and classification of MIKC-type MADS-box genes in Streptophyte lineages and expression analyses to reveal their role in seed germination of orchid
Source: BMC Plant Biol. 2019 May 28;19:223. doi: 10.1186/s12870-019-1836-5 (PMC6540398; doi:10.1186/s12870-019-1836-5)
Supplement: Supplementary file 4 — Table S2. Information of MADS-box genes in Arabidopsis thaliana, Oryza sativa, Brassica rapa, Brachypodium distachyon, Zea mays, Sorghum bicolor, Phalaenopsis equestris, Dendrobium officinale, Physcomitrella patens, and Selaginella moellendorffii. (DOCX 17 kb) [file 12870_2019_1836_MOESM4_ESM.docx]

**Table S2**: **Information of MADS-box genes in *Arabidopsis thaliana*, *Oryza sativa*, *Brassica rapa*, *Brachypodium distachyon*, *Zea mays*, *Sorghum bicolor*, *Phalaenopsis equestris*, *Dendrobium officinale*, *Physcomitrella patens*, and *Selaginella* *moellendorffii.***

| **Species** | **Type I** | **Type II** | | **Total** | **Reference** |  |
| --- | --- | --- | --- | --- | --- | --- |
|  |  | **MIKC*** | **MIKCC** |  |  |  |
| *Arabidopsis thaliana* | 62 (62) | 7 (7) | 39 (39) | 108 (108) | Pařenicová et al. 2003 | |
| *Oryza sativa* | 32 (29) | 5 (3) | 38 (41) | 75 (73) | Arora et al. 2007 | |
| *Brassica rapa* | 65 (64) | 11 (11) | 84 (85) | 160 (160) | Duan et al. 2015 | |
| *Brachypodium distachyon* | 18 (17) | 7 (2) | 32 (39) | 57 (58) | Wei et al. 2014 | |
| *Zea mays* | 32 (18） | 4 (4) | 39 (73) | 75 (95) | Zhao et al. 2011 | |
| *Sorghum bicolor* | 30 (25) | 2 (2) | 33 (38) | 65 (65) | Zhao et al. 2012 | |
| *Phalaenopsis equestris* | 22 (14) | 1 (4) | 28 (30) | 51 (48) | Cai et al. 2015 | |
| *Dendrobium officinale* | 28 (27) | 3 (8) | 32 (33) | 63 (68) | Zhang et al. 2016 | |
| *Physcomitrella patens* | 7 (8) | 11 (11) | 6 (6) | 24 (25) | Barker and Ashton 2013 | |
| *Selaginella moellendorffii* | 13 (27) | 3 (7) | 3 (6) | 19 (40) | Gramzow et al. 2012 | |

Note: Numbers indicate gene number in a clade or group in previous studies. Numbers in brackets indicate gene numbers in a clade or group in this study.

| **References** |
| --- |
| Arora, R., Agarwal, P., Ray, S., Singh, A.K., Singh, V.P., Tyagi, A.K., et al. (2007) MADS-box gene family in rice: genome-wide identification, organization and expression profiling during reproductive development and stress. BMC Genomics 8: 242. |
| Barker, E.I. and Ashton, N.W. (2013) A parsimonious model of lineage-specific expansion of MADS-box genes in *Physcomitrella patens*. Plant Cell Reports 32: 1161-1177. |
| Cai, J., Liu, X., Vanneste, K., Proost, S., Tsai, W.C., Liu, K.W., et al. (2015) The genome sequence of the orchid *Phalaenopsis equestris*. Nature Genetics 47: 65-72. |
| Duan, W., Song, X., Liu, T., Huang, Z., Ren, J., Hou, X., et al. (2015) Genome-wide analysis of the MADS-box gene family in *Brassica rapa* (Chinese cabbage). Molecular Genetics and Genomics 290: 239-255. |
| Gramzow, L., Barker, E., Schulz, C., Ambrose, B., Ashton, N., Theissen, G., et al. (2012) *Selaginella* genome analysis – entering the “homoplasy heaven” of the MADS world. Frontiers in Plant Science 3: 214. |
| Pařenicová, L., De, S.F., Kieffer, M., Horner, D.S., Favalli, C., Busscher, J., et al. (2003) Molecular and phylogenetic analyses of the complete MADS-box transcription factor family in Arabidopsis: new openings to the MADS world. Plant Cell 15: 1538-1551. |
| Wei, B., Zhang, R.Z., Guo, J.J., Liu, D.M., Li, A.L., Fan, R.C., et al. (2014) Genome-wide analysis of the MADS-box gene family in *Brachypodium distachyon*. Genome 9: e84781. |
| Zhang, G.-Q., Xu, Q., Bian, C., Tsai, W.-C., Yeh, C.-M., Liu, K.-W., et al. (2016) The *Dendrobium catenatum* Lindl. genome sequence provides insights into polysaccharide synthase, floral development and adaptive evolution. Scientific reports 6: 19029.  Zhao, Y., Li, X., Chen, W., Peng, X., Cheng, X., Zhu, S., et al. (2011) Whole-genome survey and characterization of MADS-box gene family in maize and sorghum. Plant Cell Tissue & Organ Culture 105: 159-173. |
