# Supplementary material for: Genome-wide identification and classification of MIKC-type MADS-box genes in Streptophyte lineages and expression analyses to reveal their role in seed germination of orchid
Source: BMC Plant Biol. 2019 May 28;19:223. doi: 10.1186/s12870-019-1836-5 (PMC6540398; doi:10.1186/s12870-019-1836-5)
Supplement: Supplementary file 5 — Figure S3. Classification of the total of 983 MIKC-type proteins based on a phylogenetic analysis. MIKC-type proteins can be divided into two main groups MIKC* and MIKCC. The phylogenetic tree was constructed by MEGA 7 with the NJ method based on MAFFT 7-based alignments. (DOCX 767 kb) [file 12870_2019_1836_MOESM5_ESM.docx]

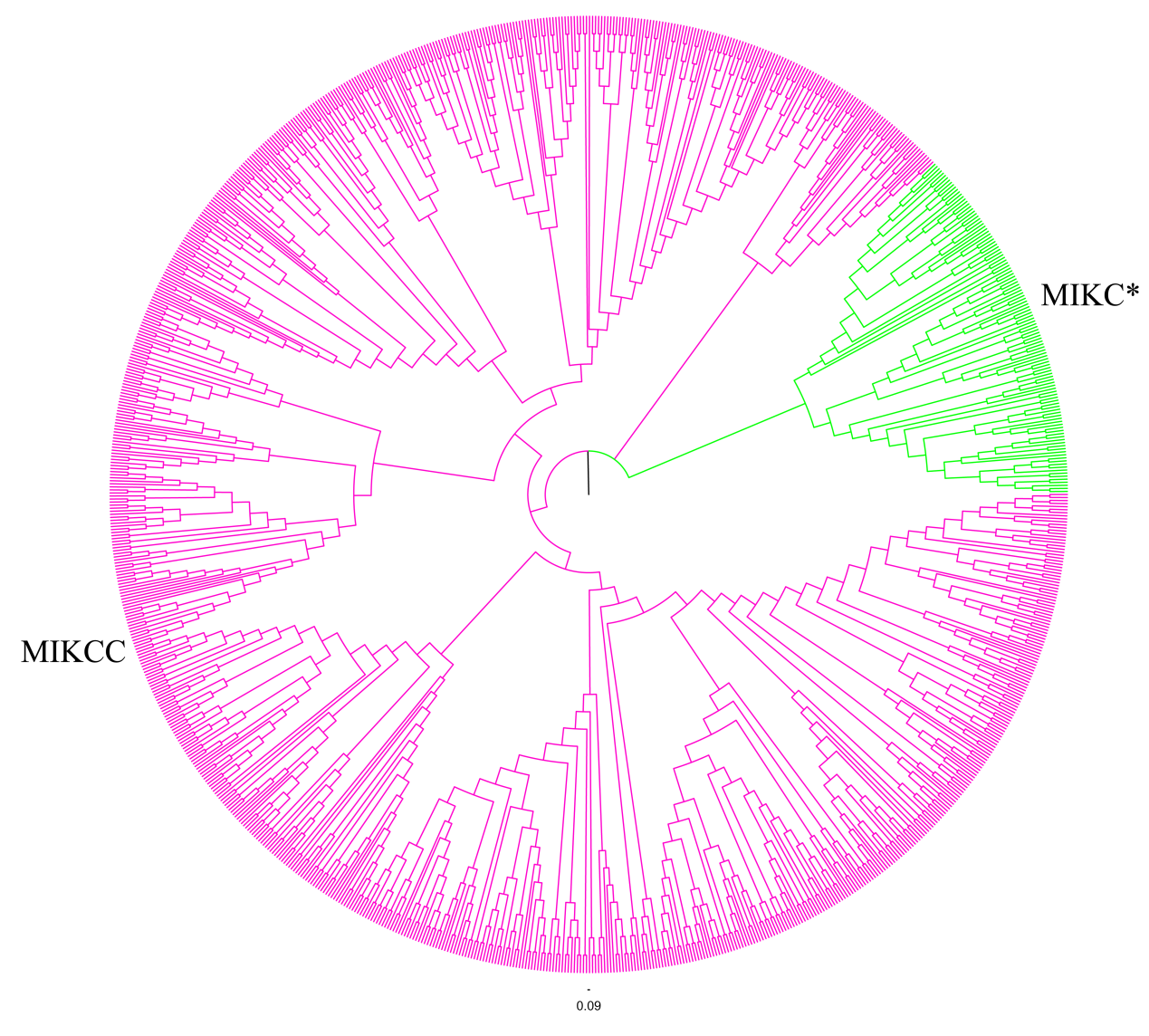


Figure S3 Classification of the total of 983 MIKC-type proteins based on a phylogenetic analysis. MIKC-type proteins can be divided into two main groups MIKC* and MIKCC. The phylogenetic tree was constructed by MEGA 7 with the Neighbor–Joining method based on MAFFT 7-based alignments.
