# Supplementary material for: Genome-wide identification and classification of MIKC-type MADS-box genes in Streptophyte lineages and expression analyses to reveal their role in seed germination of orchid
Source: BMC Plant Biol. 2019 May 28;19:223. doi: 10.1186/s12870-019-1836-5 (PMC6540398; doi:10.1186/s12870-019-1836-5)
Supplement: Supplementary file 6 — Figure S4. Classification of the total 865 MIKCC proteins based on phylogenetic analysis. The phylogenetic tree was conducted using MEGA 7 based on the alignment of MADS-box proteins by MAFFT 7 with the Neighbor-Joining method. Numbers besides branches represent bootstrap support values from 1000 replications. Values lower than 40% are hidden. (DOCX 2621 kb) [file 12870_2019_1836_MOESM6_ESM.docx]

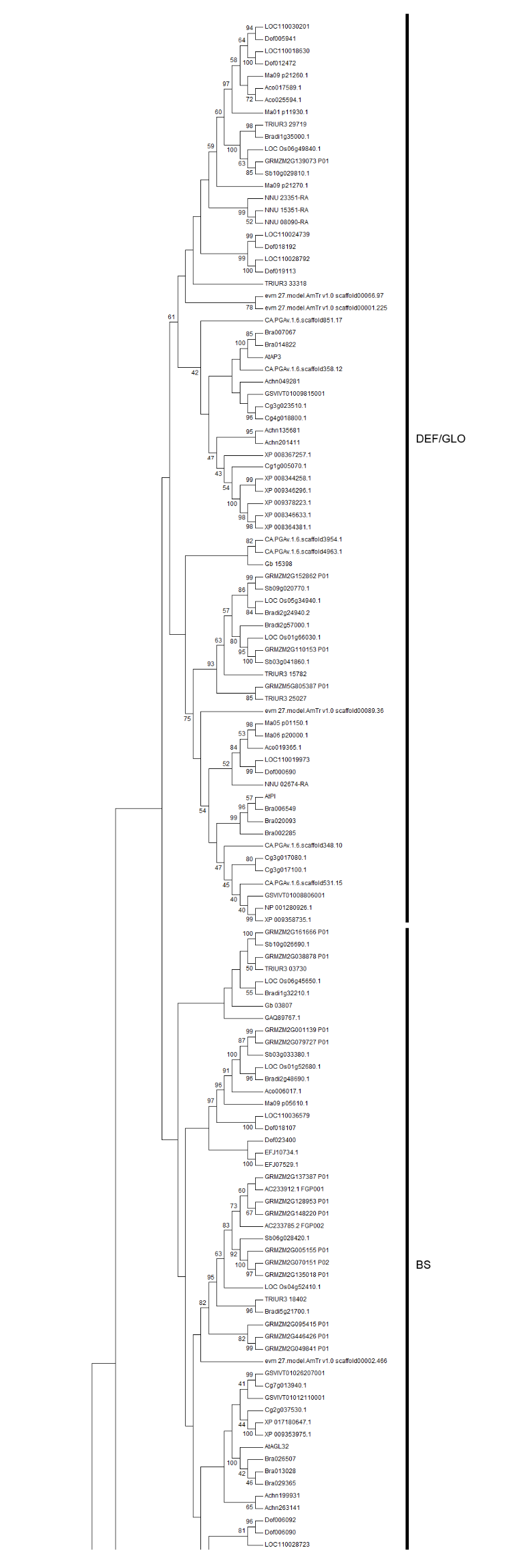


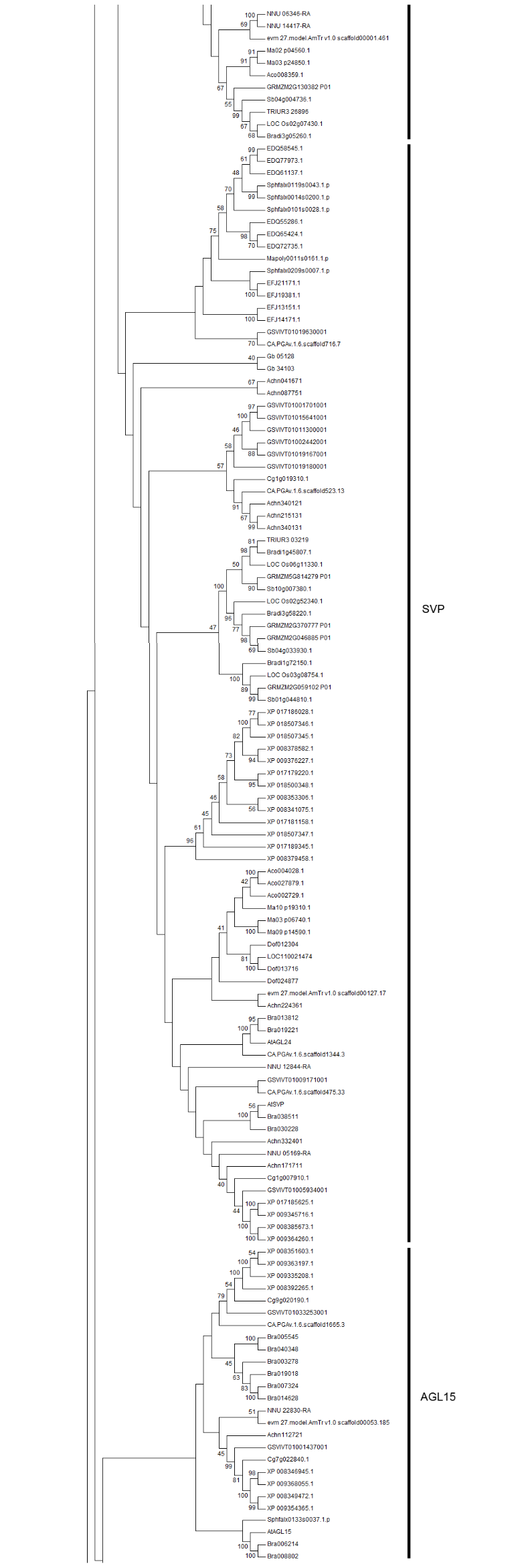


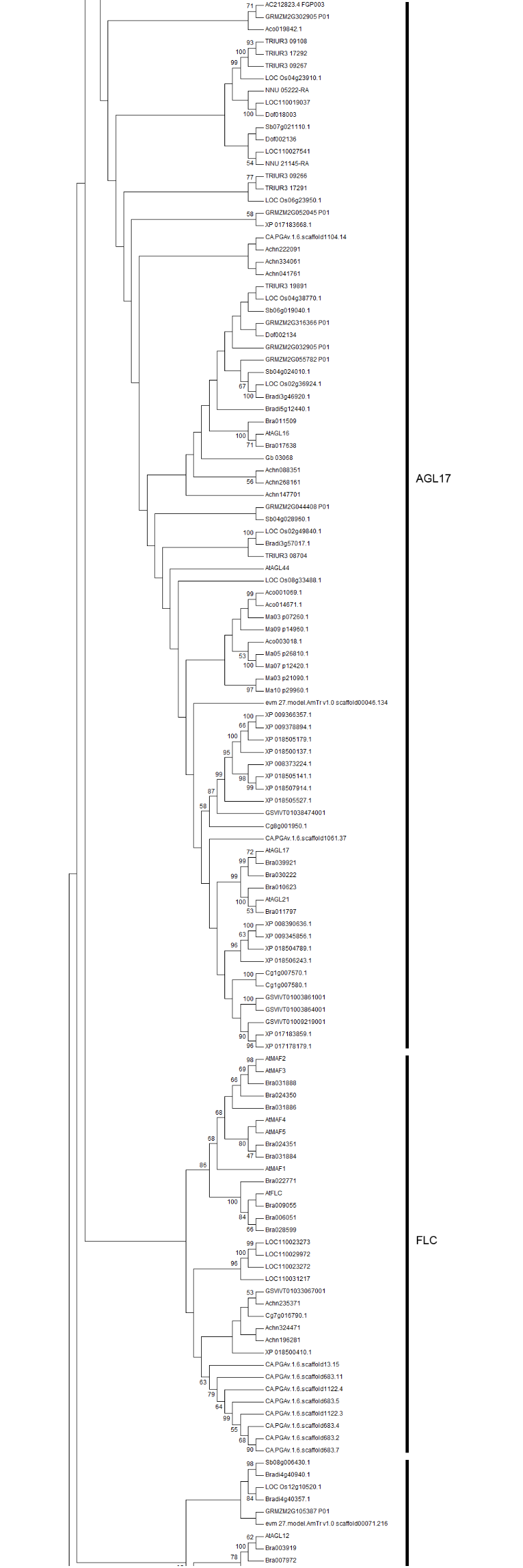


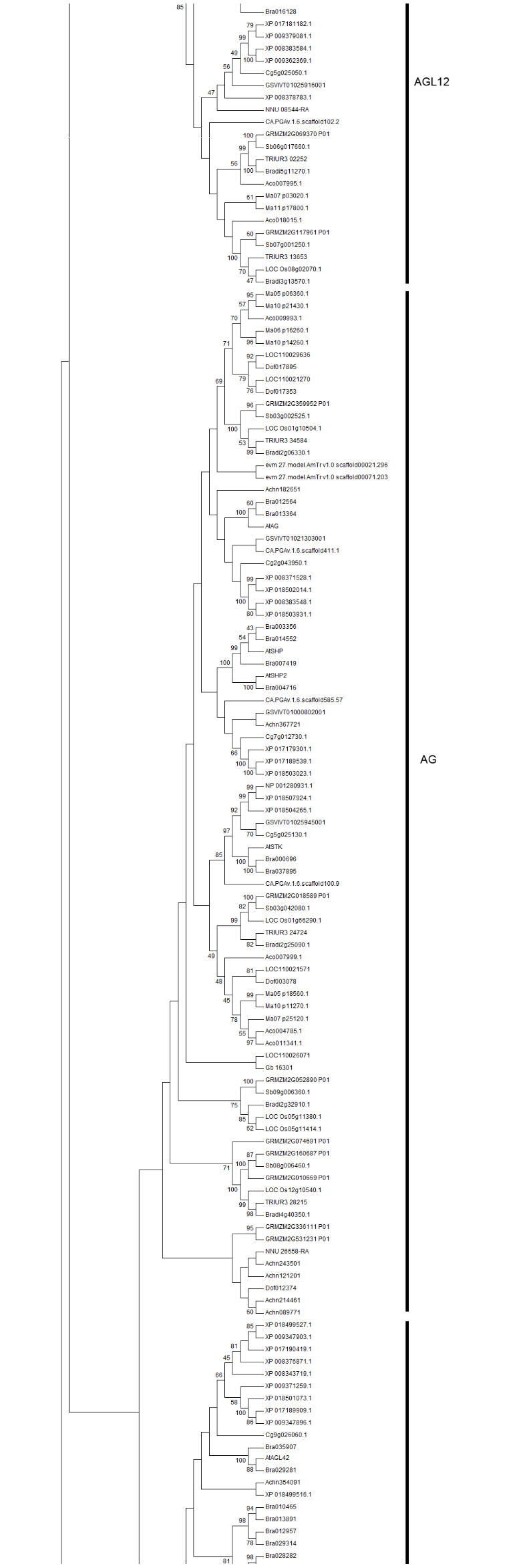


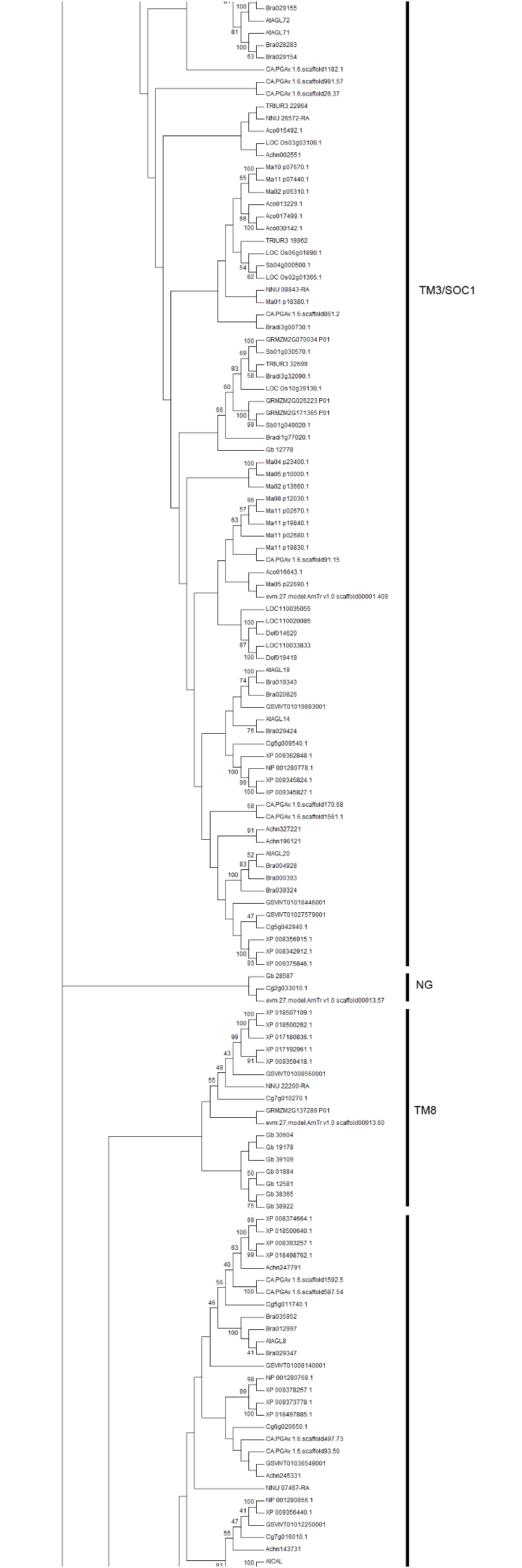


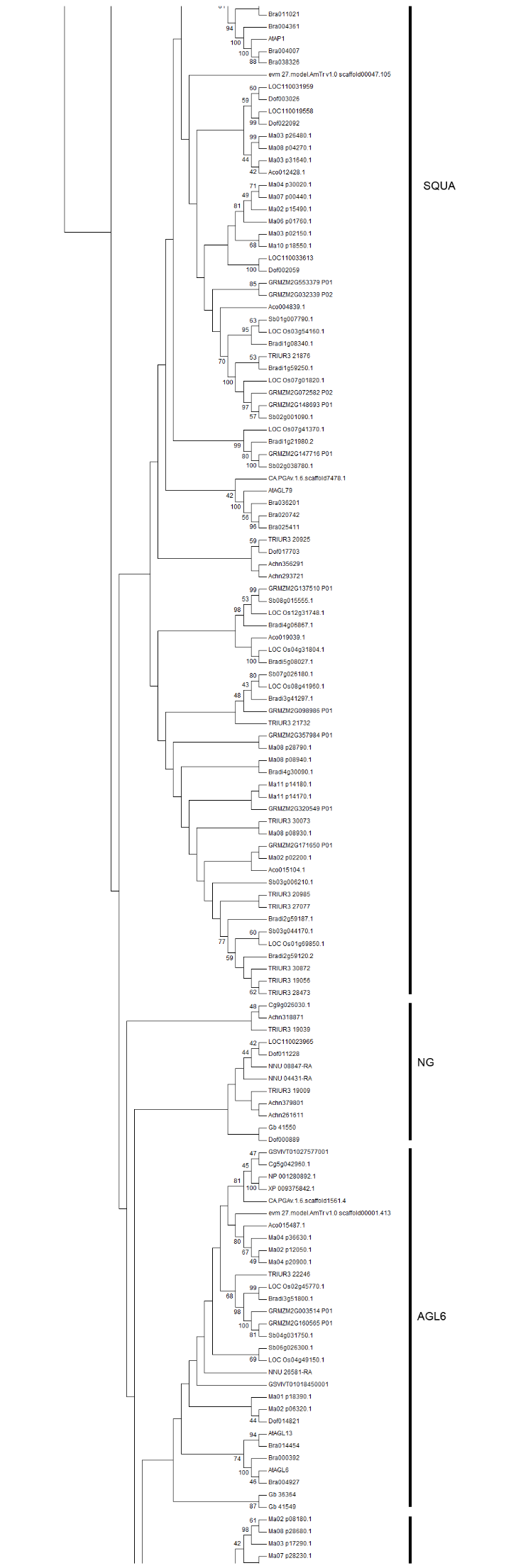


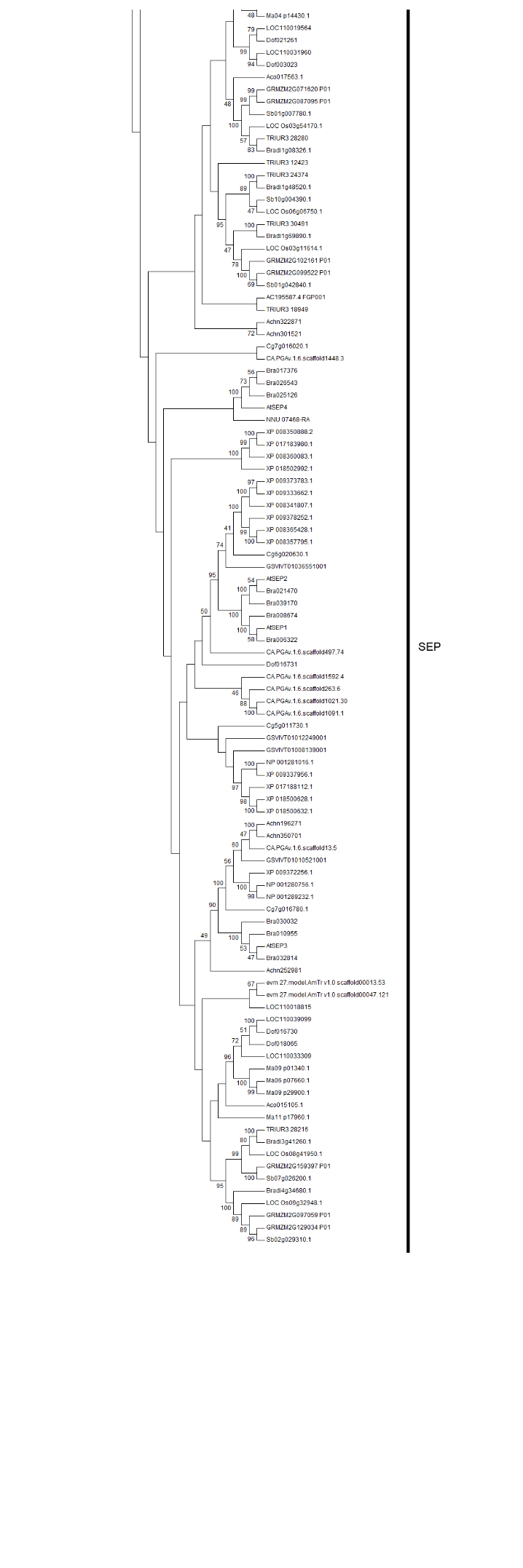


Figure S4 Classification of the total 865 MIKCC proteins based on phylogenetic analysis. The phylogenetic tree was conducted using MEGA 7 based on the alignment of MADS-box proteins by MAFFT 7 with the Neighbor-Joining method. Numbers besides branches represent bootstrap support values from 1000 replications. Values lower than 40% are hidden.
