# Supplementary material for: Genome-wide identification and classification of MIKC-type MADS-box genes in Streptophyte lineages and expression analyses to reveal their role in seed germination of orchid
Source: BMC Plant Biol. 2019 May 28;19:223. doi: 10.1186/s12870-019-1836-5 (PMC6540398; doi:10.1186/s12870-019-1836-5)
Supplement: Supplementary file 7 — Figure S5. Phylogenetic analyses of GAQ89767.1 from Klebsormidium nitens and MADS-box proteins from Arabidopsis thaliana (At). The phylogenetic tree was conducted using MEGA 7 based on the alignment of MADS-box proteins by MAFFT 7 with the NJ method. Numbers besides branches represent bootstrap support values from 1000 replications. (DOCX 428 kb) [file 12870_2019_1836_MOESM7_ESM.docx]

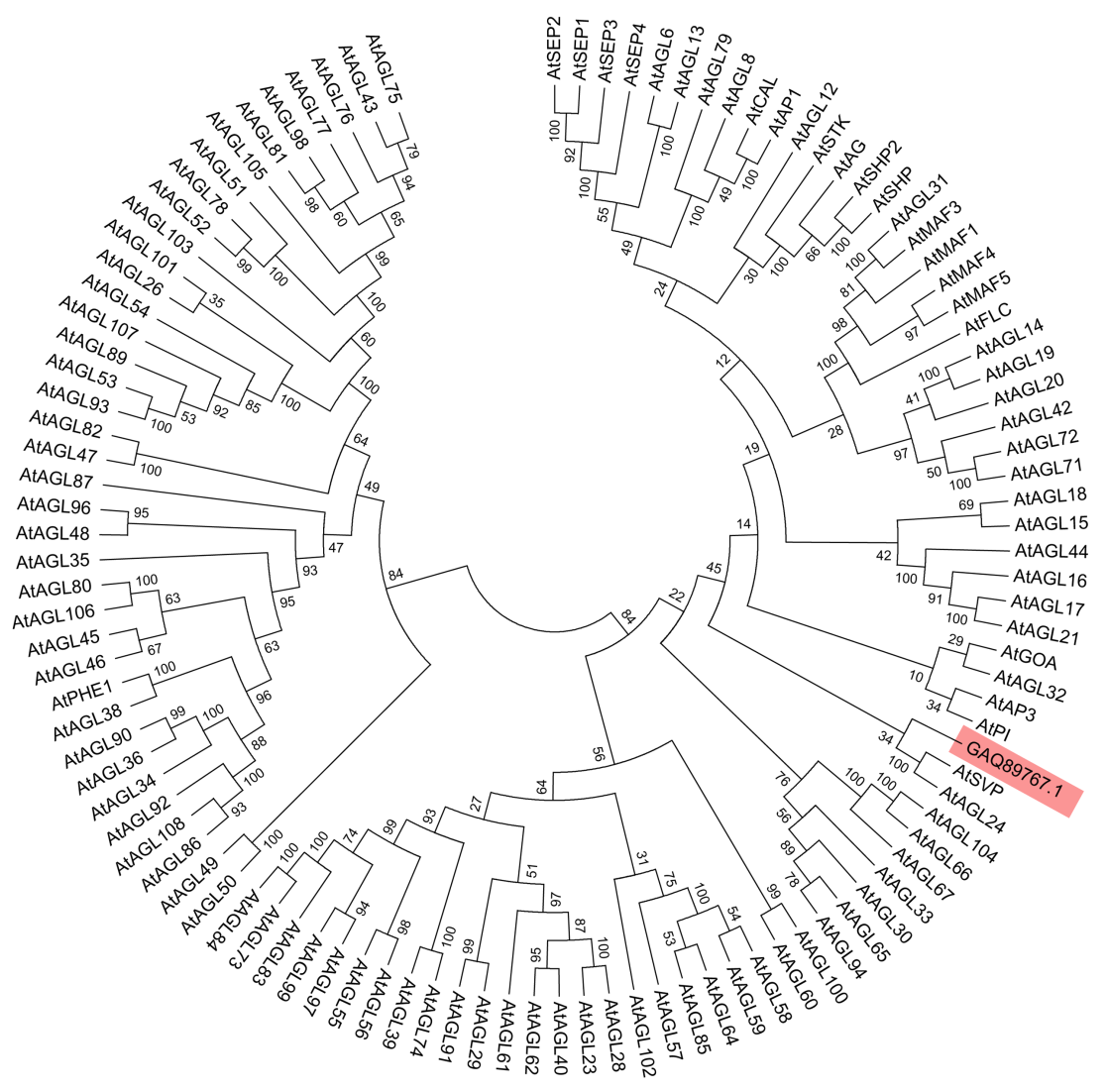


Figure S5 Phylogenetic analyses of GAQ89767.1 from *Klebsormidium nitens* and MADS-box proteins from *Arabidopsis thaliana* (At). The phylogenetic tree was conducted using MEGA 7 based on the alignment of MADS-box proteins by MAFFT 7 with the Neighbor-Joining method. Numbers besides branches represent bootstrap support values from 1000 replications.
