# Supplementary material for: Genome-wide identification and classification of MIKC-type MADS-box genes in Streptophyte lineages and expression analyses to reveal their role in seed germination of orchid
Source: BMC Plant Biol. 2019 May 28;19:223. doi: 10.1186/s12870-019-1836-5 (PMC6540398; doi:10.1186/s12870-019-1836-5)
Supplement: Supplementary file 8 — Figure S6. Phylogenetic analyses of the only two MADS-box proteins (Mapoly0011s0161.1.p and Mapoly0174s0011.1.p) from Marchantia polymorpha and MADS-box proteins from Arabidopsis thaliana (At). Mapoly0011s0161.1.p falls into the SVP subfamily (29% similarity). Mapoly0174s0011.1.p falls into the MIKC* group. The phylogenetic tree was conducted using MEGA 7 based on the alignment of MADS-box proteins by MAFFT 7 with the NJ method. Numbers besides branches represent bootstrap support values from 1000 replications. (DOCX 413 kb) [file 12870_2019_1836_MOESM8_ESM.docx]

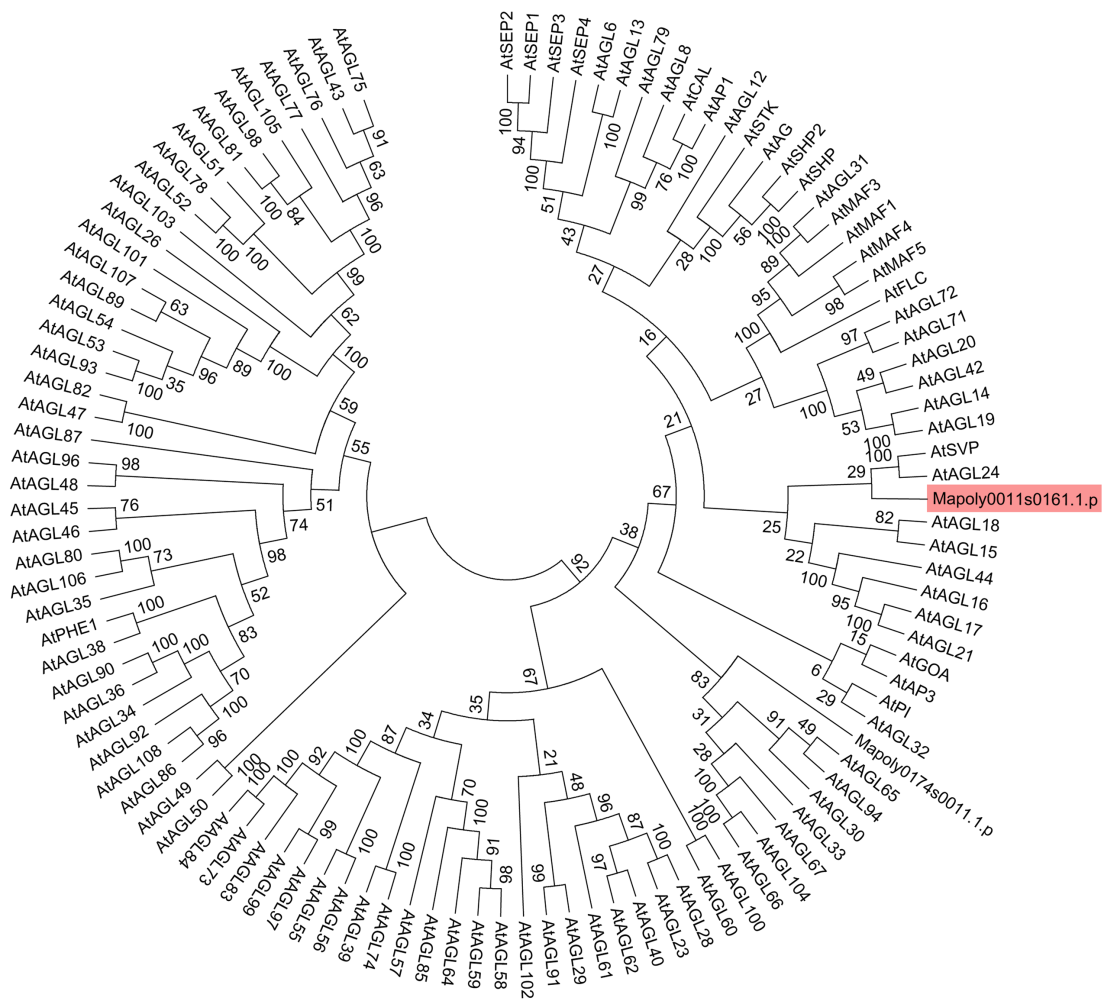


Figure S6 Phylogenetic analyses of the only two MADS-box proteins (Mapoly0011s0161.1.p and Mapoly0174s0011.1.p) from *Marchantia polymorpha* and MADS-box proteins from *Arabidopsis thaliana* (At). Mapoly0011s0161.1.p falls into the SVP subfamily (29% similarity). Mapoly0174s0011.1.p falls into the MIKC* group. The phylogenetic tree was conducted using MEGA 7 based on the alignment of MADS-box proteins by MAFFT 7 with the Neighbor-Joining method. Numbers besides branches represent bootstrap support values from 1000 replications.
