# Supplementary material for: Genome-wide identification and classification of MIKC-type MADS-box genes in Streptophyte lineages and expression analyses to reveal their role in seed germination of orchid
Source: BMC Plant Biol. 2019 May 28;19:223. doi: 10.1186/s12870-019-1836-5 (PMC6540398; doi:10.1186/s12870-019-1836-5)
Supplement: Supplementary file 9 — Table S3. The mean of FPKM value and fold change of MIKC gene under control and cold stress in D. officinale leaf. (DOCX 17 kb) [file 12870_2019_1836_MOESM9_ESM.docx]

**Table S3 The mean of FPKM value and fold change of MIKC gene under control and cold stress in *D*. *officinale* leaves.**

| Gene name | control (FPKM mean) | cold treatment (FPKM mean) | fold change | regulation |
| --- | --- | --- | --- | --- |
| DoAGL1 | 0 | 0 |  |  |
| DoAGL2 | 0.096224 | 0.067076 |  |  |
| DoAGL3 | 0 | 0 |  |  |
| DoAGL4 | 0 | 0 |  |  |
| DoAGL5 | 0 | 0 |  |  |
| DoAGL6 | 0 | 0 |  |  |
| DoAGL7 | 8.365922 | 6.014673 | 0.718949 |  |
| DoAGL8 | 2.289608 | 0.49015 |  |  |
| DoAGL9 | 0.406502 | 0.195353 |  |  |
| DoAGL10 | 1.757854 | 0.516508 |  |  |
| DoAGL11 | 0.135796 | 0.038845 |  |  |
| DoAGL12 | 2.555189 | 1.798869 |  |  |
| DoAGL13 | 2.907893 | 1.385438 |  |  |
| DoAGL14 | 0.062554 | 0.095705 |  |  |
| DoAGL15 | 0 | 0 |  |  |
| DoAGL16 | 22.16169 | 15.29727 | 0.690258 |  |
| DoAGL17 | 20.29814 | 28.42042 | 1.400149 |  |
| DoAGL18 | 9.294773 | 3.327556 | 0.358003 | down |
| DoAGL19 | 77.80525 | 51.54853 | 0.662533 |  |
| DoAGL20 | 1.751287 | 1.67083 |  |  |
| DoAGL21 | 0 | 0 |  |  |
| DoAGL22 | 0.023313 | 0 |  |  |
| DoAGL23 | 0.276598 | 0 |  |  |
| DoAGL24 | 12.69374 | 19.20036 | 1.512585 | up |
| DoAGL25 | 21.95858 | 17.54852 | 0.799164 |  |
| DoAGL26 | 0.119866 | 0.042717 |  |  |
| DoAGL27 | 14.6329 | 23.52354 | 1.607579 | up |
| DoAGL28 | 25.34711 | 26.43874 | 1.043067 |  |
| DoAGL29 | 111.8417 | 142.6601 | 1.275554 |  |
| DoAGL30 | 0 | 3.468958 |  |  |
| DoAGL31 | 0.334172 | 0.093284 |  |  |
| DoAGL32 | 0.092821 | 0.484964 |  |  |
| DoAGL33 | 0 | 0.025264 |  |  |
| DoAGL34 | 16.68273 | 12.59022 | 0.754686 |  |
| DoAGL35 | 0.273996 | 0.373012 |  |  |
| DoAGL36 | 0 | 0 |  |  |
| DoAGL37 | 0 | 0 |  |  |
| DoAGL38 | 0 | 0 |  |  |
| DoAGL39 | 0.028107 | 0.083085 |  |  |
| DoAGL40 | 0.791054 | 0.382377 |  |  |
| DoAGL41 | 0.892717 | 0.685036 |  |  |
