# Supplementary material for: Genome-wide identification and classification of MIKC-type MADS-box genes in Streptophyte lineages and expression analyses to reveal their role in seed germination of orchid
Source: BMC Plant Biol. 2019 May 28;19:223. doi: 10.1186/s12870-019-1836-5 (PMC6540398; doi:10.1186/s12870-019-1836-5)
Supplement: Supplementary file 10 — Table S4. FPKM value of the MIKC gene in Dendrobium officinale during four seed germination stages. (DOCX 16 kb) [file 12870_2019_1836_MOESM10_ESM.docx]

| **Table S4 FPKM value of the MIKC gene in *Dendrobium officinale* during four seed germination stages.** |
| --- |
| \| Gene name \| S1 (FPKM) \| S2 (FPKM) \| S3 (FPKM) \| S4 (FPKM) \| \| --- \| --- \| --- \| --- \| --- \| \| DoAGL1 \| 0 \| 0 \| 0 \| 0 \| \| DoAGL2 \| 0 \| 0 \| 0 \| 0 \| \| DoAGL3 \| 0 \| 0 \| 0 \| 0 \| \| DoAGL4 \| 0 \| 0 \| 0 \| 0 \| \| DoAGL5 \| 0 \| 0 \| 0 \| 0 \| \| DoAGL6 \| 0 \| 0 \| 0 \| 0 \| \| DoAGL7 \| 177.53 \| 232.99 \| 389 \| 312.98 \| \| DoAGL8 \| 4.47 \| 2.01 \| 0 \| 4.02 \| \| DoAGL9 \| 0 \| 0 \| 0 \| 0 \| \| DoAGL10 \| 9 \| 0 \| 0 \| 0 \| \| DoAGL11 \| 0 \| 0 \| 0 \| 0 \| \| DoAGL12 \| 0 \| 0 \| 0 \| 0 \| \| DoAGL13 \| 58 \| 11 \| 3 \| 3 \| \| DoAGL14 \| 0 \| 0 \| 0 \| 0 \| \| DoAGL15 \| 0 \| 0 \| 0 \| 0 \| \| DoAGL16 \| 0 \| 0 \| 0 \| 0 \| \| DoAGL17 \| 0 \| 5 \| 7 \| 3 \| \| DoAGL18 \| 2 \| 18 \| 5 \| 33 \| \| DoAGL19 \| 12 \| 2 \| 4 \| 3 \| \| DoAGL20 \| 73 \| 47 \| 56 \| 26 \| \| DoAGL21 \| 14 \| 0 \| 0 \| 0 \| \| DoAGL22 \| 2 \| 0 \| 6 \| 13 \| \| DoAGL23 \| 0 \| 0 \| 0 \| 0 \| \| DoAGL24 \| 2 \| 0 \| 0 \| 0 \| \| DoAGL25 \| 6 \| 1 \| 0 \| 0 \| \| DoAGL26 \| 3 \| 0 \| 2 \| 10 \| \| DoAGL27 \| 0 \| 2 \| 9 \| 4 \| \| DoAGL28 \| 15 \| 3 \| 1 \| 9 \| \| DoAGL29 \| 3 \| 7 \| 6 \| 7 \| \| DoAGL30 \| 18 \| 8 \| 7 \| 3 \| \| DoAGL31 \| 1 \| 0 \| 0 \| 0 \| \| DoAGL32 \| 1 \| 154 \| 168 \| 325 \| \| DoAGL33 \| 1 \| 1 \| 0 \| 4 \| \| DoAGL34 \| 184 \| 154 \| 196 \| 357 \| \| DoAGL35 \| 0 \| 0 \| 9 \| 6 \| \| DoAGL36 \| 2 \| 0 \| 0 \| 0 \| \| DoAGL37 \| 5 \| 0 \| 0 \| 0 \| \| DoAGL38 \| 0 \| 0 \| 0 \| 0 \| \| DoAGL39 \| 0 \| 0 \| 0 \| 1 \| \| DoAGL40 \| 0 \| 1 \| 0 \| 2 \| \| DoAGL41 \| 0 \| 0 \| 0 \| 1 \| |
