# Supplementary material for: Genome-wide identification and classification of MIKC-type MADS-box genes in Streptophyte lineages and expression analyses to reveal their role in seed germination of orchid
Source: BMC Plant Biol. 2019 May 28;19:223. doi: 10.1186/s12870-019-1836-5 (PMC6540398; doi:10.1186/s12870-019-1836-5)
Supplement: Supplementary file 11 — Table S5. The versin of genomic data and download websites. (DOCX 16 kb) [file 12870_2019_1836_MOESM11_ESM.docx]

| **Table S5 The version of genomic data and download websites.** | | |
| --- | --- | --- |
| **Species** | **Version** | **Download URL (website)** |
| *Arabidopsis thaliana* | TAIR10 | https://www.arabidopsis.org/ |
| *Brassica rapa* | Brapa_197 | http://www.plantgdb.org/ |
| *Actinidia chinensis* | v1.0 | http://bioinfo.bti.cornell.edu/cgi-bin/kiwi/download.cgi |
| *Citrus grandis* | version1 | http://citrus.hzau.edu.cn/orange/index.php |
| *Pyrus x bretschneideri* | v1.0 | tp://ftp.ncbi.nlm.nih.gov/genomes/all/GCA/000/315/295/GCA_000315295.1_Pbr_v1.0/ |
| *Malus domestica* | Version 1.0 | http://www.applegene.org/ |
| *Capsicum annuum* | v.2.0 | http://peppergenome.snu.ac.kr/ |
| *Vitis vinifera* | Version 1.0 | http://www.genoscope.cns.fr/externe/GenomeBrowser/Vitis/ |
| *Zea mays* | ZmGDB181 | http://www.plantgdb.org/ |
| *Sorghum bicolor* | Sbicolor_79 | http://www.plantgdb.org/ |
| *Triticum aestivum* | NM | http://www.filewatcher.com/ |
| *Brachypodium distachyon* | Bdistachyon_192 | http://www.plantgdb.org/ |
| *Oryza sativa* | Osativa_193 | http://www.plantgdb.org/ |
| *Ananas comosus* | Acomosus_321_v3 | https://phytozome.jgi.doe.gov/pz/portal.html |
| *Musa acuminata* | Macuminata_304_v1 | https://phytozome.jgi.doe.gov/pz/portal.html |
| *Phalaenopsis equestris* | NM | https://www.ncbi.nlm.nih.gov/assembly/GCF_001263595.1/ |
| *Dendrobium officinale* | NM | http://www.ncbi.nlm.nih.gov/bioproject/262478 |
| *Nelumbo nucifera* | NM | http://lotus-db.wbgcas.cn/ |
| *Amborella trichopoda* | version 1.0 | http://amborella.huck.psu.edu/data |
| *Ginkgo biloba* | NM | http://tgwebprod.cam.uchc.edu/Drupal/ |
| *Selaginella moellendorffii* | NM | https://www.ncbi.nlm.nih.gov/assembly/GCA_000143415.2/ |
| *Physcomitrella patens* | NM | https://www.ncbi.nlm.nih.gov/assembly/GCA_000002425.1/ |
| *Sphagnum fallax* | Sfallax_310_v0.5 | https://phytozome.jgi.doe.gov/pz/portal.html |
| *Marchantia polymorpha* | Mpolymorpha_320_v3.1 | https://phytozome.jgi.doe.gov/pz/portal.html |
| *Klebsormidium nitens* | V1.0 | http://www.plantmorphogenesis.bio.titech.ac.jp/~algae_genome_project/klebsormidium/index.html |
| *Chlamydomonas reinhardtii* | Creinhardtii_169 | http://www.plantgdb.org/ |
| *Volvox carteri* | Vcarteri_317_v2.1 | https://phytozome.jgi.doe.gov/pz/portal.html |
| *Dunaliella salina* | Dsalina_325_v1.0 | https://phytozome.jgi.doe.gov/pz/portal.html |
| *Micromonas pusilla* CCMP1545 | MpusillaCCMP1545_228_v3.0 | https://phytozome.jgi.doe.gov/pz/portal.html |
|  |  |  |
| Note: NM, not mentioned. |  |  |
