# Supplementary material for: Genome-wide identification and classification of MIKC-type MADS-box genes in Streptophyte lineages and expression analyses to reveal their role in seed germination of orchid
Source: BMC Plant Biol. 2019 May 28;19:223. doi: 10.1186/s12870-019-1836-5 (PMC6540398; doi:10.1186/s12870-019-1836-5)
Supplement: Supplementary file 12 — Table S6. The primers used for Semi-quantitative RT-PCR. (DOCX 14 kb) [file 12870_2019_1836_MOESM12_ESM.docx]

| **Table S6 The primers used for Semi-quantitative RT-PCR.** | |
| --- | --- |
| **Primer name** | **Primer sequence** |
| DoAGL9F | 5′-GGAAACACAGAGTAGTCAGCAGGA-3′ |
| DoAGL9R | 5′-CAGGTGGCATGTAATTATTGACAT-3′ |
| DoAGL10R | 5′-GGTGCCATTGTTATATCAGAATGA-3′ |
| DoAGL12F | 5′-ACAATTATCAGGAGTATTTGAAGCT-3′ |
| DoAGL12R | 5′-CCTGTTCAACTGTTCATCTGTACAG-3′ |
| DoAGL13F | 5′-TGAATATCTACAACGGTCACAGAGG-3′ |
| DoAGL13R | 5′-GGACTGTATCCAATTTGCAGAGAAG-3′ |
| DoAGL16F | 5′-GGGAGGGGAAGGGTGCAGTTGAGG-3′ |
| DoAGL16R | 5′-CCCCCACCACCCAAAACTCGCTT-3′ |
| DoAGL17F | 5′-ACAGAAGAGCCAAAGGCACCTTATG-3′ |
| DoAGL17R | 5′-ATGACTGAGCATCCATGGTGGTAGT-3′ |
| DoAGL18F | 5′-CGAAGCCATCTCATGGGTGAGAAG-3′ |
| DoAGL18R | 5′-GGTGGGAAGGGAGTTTGTTGCCTCC-3′ |
| DoAGL19F | 5′-GTTTGAAAGAGCTTCAACACTTGGA-3′ |
| DoAGL19R | 5′-TATTCACCCGAGTAAGAGGAGGCT-3′ |
| DoAGL21F | 5′-GTGCTGATAATTCCAATTCAGGATC-3′ |
| DoAGL21R | 5′-CAGTCTGCTGTTGTAAAGAATAGTG-3′ |
| DoAGL22F | 5′-AATTTGCAGAACTCAAACAGGAAT-3′ |
| DoAGL22R | 5′-AAAGCTTCGAGAATCAAATGGAGG-3′ |
| DoAGL30F | 5′-AAGATGCAGAATACGCTGAACCA-3′ |
| DoAGL30R | 5′-TTCATGAAGGTTTGGATGGCTG-3′ |
| DoAGL31F | 5′-CCGTCCACTGACTCGAAGAGTAT-3′ |
| DoAGL31R | 5′-GGTTGGGTACGGAATGAGTACAAG-3′ |
| DoAGL32F | 5′-AGGCCGAATACCAGAGGATGCAGA-3′ |
| DoAGL32R | 5′-TAAACTATTTTCTGCTTATTTGGC-3′ |
| DoAGL33F | 5′-ATGGAATGCTCAATATGAGAGGAT-3′ |
| DoAGL33R | 5′-AGATCATGTGATTCGTATCCTATA-3′ |
| DoAGL34F | 5′-CAGGCATTTGAAAGGGGAGGATCT-3′ |
| DoAGL34R | 5′-GTTTGGCTGGATGGGCTGCACACG-3′ |
| ActinF | 5′-GCGGACGTTGATGATATTCAGCCTC-3′ |
| ActinR | 5′-GAATGTGCTGAGGGAGGCAAGGATAG-3′ |
